# Distinct Pigmentary and Melanocortin 1 Receptor–Dependent Components of Cutaneous Defense against Ultraviolet Radiation

**DOI:** 10.1371/journal.pgen.0030009

**Published:** 2007-01-12

**Authors:** Craig S April, Gregory S Barsh

**Affiliations:** 1 Department of Genetics, Stanford University School of Medicine, Stanford, California, United States of America; 2 Department of Pediatrics, Stanford University School of Medicine, Stanford, California, United States of America; The Jackson Laboratory, United States of America

## Abstract

Genetic variation at the melanocortin 1 receptor *(MC1R)* is an important risk factor for developing ultraviolet (UV) radiation–induced skin cancer, the most common form of cancer in humans. The underlying mechanisms by which the MC1R defends against UV-induced skin cancer are not known. We used neonatal mouse skin (which, like human skin, contains a mixture of melanocytes and keratinocytes) to study how pigment cells and *Mc1r* genotype affect the genome-level response to UV radiation. Animals without viable melanocytes *(Kit^W-v^/Kit^W-v^)* or animals lacking a functional Mc1r *(Mc1r^e^/Mc1r^e^)* were exposed to sunburn-level doses of UVB radiation, and the patterns of large-scale gene expression in the basal epidermis were compared to each other and to nonmutant animals. Our analysis revealed discrete Kit- and Mc1r-dependent UVB transcriptional responses in the basal epidermis. The Kit-dependent UVB response was characterized largely by an enrichment of oxidative and endoplasmic reticulum stress genes, highlighting a distinctive role for pigmented melanocytes in mediating antioxidant defenses against genotoxic stresses within the basal epidermal environment. By contrast, the Mc1r-dependent UVB response contained an abundance of genes associated with regulating the cell cycle and oncogenesis. To test the clinical relevance of these observations, we analyzed publicly available data sets for primary melanoma and melanoma metastases and found that the set of genes specific for the Mc1r-dependent UVB response was able to differentiate between different clinical subtypes. Our analysis also revealed that the classes of genes induced by UVB differ from those repressed by UVB with regard to their biological functions, their overall number, and their size. The findings described here offer new insights into the transcriptional nature of the UV response in the skin and provide a molecular framework for the underlying mechanisms by which melanocytes and the Mc1r independently mediate and afford protection against UV radiation.

## Author Summary

Skin cancer is the most common type of cancer in humans and annually accounts for approximately 60,000 deaths worldwide. The most important factors causally linked to skin cancer susceptibility are inadequate protection against ultraviolet (UV) B radiation, fair skin color, and variation of the melanocortin 1 receptor *(MC1R)* gene. We used cDNA microarrays to measure the genome-wide transcriptional responses to UVB irradiation in the epidermis of neonatal mice (which approximates the human basal epidermis in its cellular composition and general physiology). To investigate how pigment cells (melanocytes) and MC1R afford protection against UVB radiation, we compared results from normal mice to those from mutant mice that lacked either melanocytes *(Kit^W-v^/Kit^W-v^)* or a functional Mc1r *(Mc1r^e^/Mc1r^e^).* We identified melanocyte- and Mc1r-dependent UVB gene expression profiles in the basal epidermis. Surprisingly, the melanocyte- and Mc1r-dependent UVB responses highlighted distinct functions, with the former largely mediating antioxidant defenses and the latter regulating the cell cycle and susceptibility to oncogenesis. We also demonstrated that a subset of Mc1r-dependent UVB-responsive genes could discriminate among human melanoma subtypes, thereby suggesting a mechanism by which *MC1R* gene variants may predispose toward skin cancer.

## Introduction

One of the most important functions of cutaneous pigmentation in humans is protection against the damaging effects of ultraviolet (UV) radiation. Depending on wavelength and intensity, UV radiation can have broad-ranging effects on DNA damage, cell cycle arrest, and apoptosis [1] in virtually every one of the more than 25 differentiated cell types in the skin, as well as more specialized responses such as immunosuppression, vitamin D synthesis, and sunburn/tanning [2]. Inadequate protection against UV radiation is a major contributor to melanoma and nonmelanoma skin cancer and a significant public health concern in many populations [3].

Pigmentary defenses against UV radiation depend on both quantitative variation in the number, size, and arrangement of melanosomes—pigment granules transferred from melanocytes to surrounding keratinocytes—and qualitative variation in the type of pigment, eumelanin or pheomelanin, made within those granules. In general, increasing skin darkness correlates closely with an increased number and size and greater dispersal of melanosomes [4,5] and with increasing amounts of both eumelanin and pheomelanin (although eumelanin predominates) [6,7]; this quantitative variation is controlled by polygenic inheritance. By contrast, a single major gene, melanocortin 1 receptor *(MC1R),* can regulate the ratio of eumelanin to pheomelanin. Complete loss-of-function for *MC1R,* which encodes a seven-transmembrane receptor coupled to adenylate cyclase, produces a so-called red hair color phenotype in individuals of northern European ancestry—bright red hair, fair skin, freckling, inability to tan, and increased susceptibility to sunburn—and is associated with nearly exclusive production of pheomelanin [8–10]. Pedigree studies are generally consistent with Mendelian expectations for recessive inheritance of the red hair color phenotype [11,12], but in many populations there is considerable *MC1R* diversity with a range of hypomorphic alleles [13–16], and several association studies suggest a more complex relationship between *MC1R* genotype and pigmentary phenotype that depends on both gene dosage [12,17,18] and biochemical activity [19–21] of specific alleles.

In addition to effects on pigmentation, *MC1R* loss of function is associated with an increased incidence of melanoma and nonmelanoma skin cancer in Australian [22,23], Mediterranean [24–26], and northern European [27,28] populations. Although fair skin is a well-known risk factor for skin cancer, most of the aforementioned studies conclude that increased skin cancer susceptibility due to *MC1R* variation cannot be explained solely by pigmentary phenotype. Potential explanations for this remarkable finding include differential effects of melanin subtypes on cancer susceptibility that have not or cannot be captured by skin spectrophotometry or categorical classification of skin color and/or a protective effect of MC1R signaling that occurs independently of pigmentation. Supporting the former idea are in vitro and/or cell culture studies demonstrating that pheomelanin is phototoxic [29,30], generating reactive oxygen species [31–33] and promoting cell death [34,35] in response to UV radiation. However, differences in melanin subtype seem unlikely to explain all of the protective effects against skin cancer afforded by normal *MC1R* genotype, since both pheomelanin and eumelanin are positively correlated with skin color darkness and the content of both increases following UV radiation [6]. Indeed, several investigators have suggested that UV and MC1R signaling have synergistic actions on both pigmentation and susceptibility to skin cancer [27,36–38].

Much of what we know about how UV radiation affects skin physiology is based on cell culture studies in which particular behavioral aspects, like the extent of survival, melanogenesis, proliferation, dendricity, or DNA repair, were measured in a specific skin cell type, like melanocytes, upon exposure to UV radiation [39–41]. More recently, large-scale gene expression studies have been carried out on a variety of primary or established cell lines exposed to different doses and wavelengths of UV. These approaches have begun to show how the response to DNA damage elicits changes in cell biology that ultimately translate into whole animal phenotypes. To better understand how the skin responds to UV radiation as an integrated tissue, we have examined the whole genome transcriptional response to UVB radiation in neonatal mouse skin using a combination of genomic, genetic, and bioinformatic approaches. Although adult mouse skin lacks epidermal melanocytes in hairy areas (and therefore differs substantially from the histologic architecture of human skin), neonatal mouse skin has an abundance of active epidermal melanocytes [42,43] and a histologic architecture that shares many features with human skin [44]. Most important, a mouse model provides genetic tools to directly assess the role of melanocytes and *MC1R* genotype. Notably, our results identify a specific signature for Mc1r-dependent UVB transcriptional responses, characterized by a predominance of genes implicated in the cell cycle and oncogenesis. The Mc1r-dependent UVB transcriptional response is clinically relevant, since the genes identified in our study can partition two human melanoma data sets into clinical subtypes. This work provides new insight into the cutaneous response to UV radiation and reveals mechanisms whereby interactions between UV radiation and MC1R signaling may predispose toward the pathogenesis of skin cancer.

## Results

### A UVB-Responsive Transcriptional Profile in the Basal Epidermis

Between postnatal day 1.5 (P1.5) and P3.5 when hair follicles are still developing, melanocytes are relatively abundant in the basal epidermis, which provides a useful model to study melanocyte–keratinocyte interactions. In control experiments to examine how the transcriptional response to UV radiation changes over time, we determined that 24 h captures both early and intermediate response genes and provides substantial information in terms of the underlying gene expression profiles (unpublished data). For the experiments described below, P1.5 mice were exposed to a single dose of 100 mJ/cm^2^ UVB radiation, which corresponds to mild sunburn, approximately one minimal erythemal dose; control animals underwent the identical procedure but were shielded from UV radiation with a black Perspex box. At 24 h after exposure, we isolated basal epidermis and compared the patterns of gene expression among different experimental groups.

Our preparation of basal epidermis is based on a combination of enzymatic and physical dissociation steps that separates whole epidermal sheets, basal epidermal cells, and whole skins; in previous studies we have shown that this approach yields skin layer–specific gene expression signatures that correspond to the known biological functions of these compartments and in which the basal epidermal compartment is composed of mostly keratinocytes and melanocytes. All experiments were carried out as triplicate biological replicates (each replicate consisted of a separate pool containing two to four animals) with a common reference as described previously.

**Figure 1:**
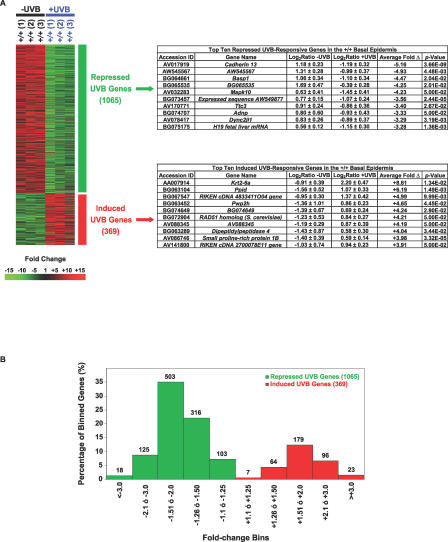
UVB Transcriptional Profile in the Basal Epidermis of Normal (*+/+*) P2.5 Mice (A) Heatmap of UVB-responsive genes. The ordered columns represent replicate samples of sham- (black) and UVB- (blue) irradiated mice, and the rows represent log_2_ ratios for 1,434 UVB-responsive genes. Data are displayed as a pseudocolor heatmap where red, green, and black represent induction, repression, and no change, respectively, relative to the median. Gray represents missing data. The ratio fold-change is indicated by a scale bar. Genes are ranked by their average fold change (Δ) value. The average fold Δ value was obtained by dividing the average expression level of each gene in the experimental (UVB-irradiated) group by that in the control (sham-irradiated) group. Average expression levels for the experimental and control groups are represented as log_2_ ratios ±SEM. The top ten ranked genes for the repressed and induced responses are listed along with their corresponding average fold change (Δ) and Student's *t*-test *p*-values. In some cases, official mouse gene symbols are used, instead of the lengthier gene names. (B) Distribution of fold-change differences for UVB-responsive genes. Fold-change differences were obtained by comparing sham- and UVB-irradiated mice. Data were binned into one of five fold-change classes for each of the repressed and induced responses. The total number of genes sorted into each bin is shown above each class.

To establish a baseline, we first determined how UVB affects gene expression in C57BL/6J animals. From spotted cDNA arrays containing 31,756 features, we obtained a data set of 13,508 cDNAs that passed quality control filters for signal intensity and replication. At a false discovery rate (FDR) of 0.05 [45], 1,434 of the 13,508 cDNAs were differentially regulated by UVB (Figure 1A and Table S1). The distribution of quantitative changes in expression was similar for induction compared to repression (Figure 1B); however, there was a striking difference in the number of cDNAs affected, with many more repressed than induced by UVB: 1,065 versus 369. Large-scale transcriptional repression has also been observed in other microarray studies of UV radiation. Indeed, the cDNA whose UVB-induced repression exhibited the most significant *p*-value in our data set was *Polr2e,* one of a dozen subunits of RNA polymerase II.

Annotation using UniGene (http://www.ncbi.nlm.nih.gov/entrez/query.fcgi?db=unigene) Cluster IDs (assigned for the majority of the UVB-responsive features on the array) revealed that 770 of 1,065 of the UVB-repressed (UVBr) and 332 of 369 of the UVB-induced (UVBi) cDNAs were unique. Readily apparent are several representatives of gene classes that are well known to participate in the response to UV radiation. Examples include those involved in cell cycle arrest (*Cdk2* and *Cyclin E1*), DNA repair (*Ercc1*, *Fanc1,* and *Rad51*), and antiapoptosis (*Aatf* and *Dad1*), all of which were induced by UVB. The Tp53 pathway is known to play an important role in mediating many of these stress responses, and indeed, we found several Tp53-dependent genes in our UVB-responsive gene list, including *Mcm2, Hif1a,* and *Bcl3,* a finding that, in general, is further supported by a recent microarray study reporting a set of p53-dependent UVB-responsive genes in primary human melanocyte cultures [46].

**Figure 2:**
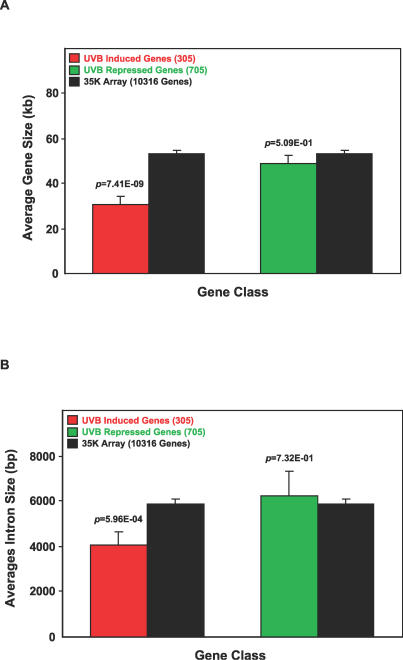
UVB Response and Gene Size Gene (A) and intron (B) size data were retrieved for 305 of 332 UVB-induced, 705 of 770 UVB-repressed, and 10,316 of 14,605 35,000 unique mouse genes (by UniGene Cluster ID criteria) using the Ensembl (National Center for Biotechnology Information m34 Assembly) and National Institute on Aging Gene Index 5.0 databases. The size data were averaged for each UVB response class and were then compared, using a Student's *t*-test, to the gene and intron size averages for 10,316 unique genes on the 35,000 array. Significance values are shown above each UVB response class, and error bars are ±SEM. Gene and intron sizes are represented here on a linear scale, but were log_2_-transformed prior to *t-*test analyses, due to the non-normal distribution of the data. Nonparametric Mann-Whitney *U* tests on the untransformed data yielded similar results.

In a recent study of UV-sensitive and p53-dependent gene expression profiles in a colorectal carcinoma cell line, McKay et al. [47] noted that genes induced by UV tended to be more compact and to have smaller introns and proposed that a gene size constraint on UV-induced mRNA expression played a key role in the evolution of UV-response pathways. Remarkably, we found a similar bias in our system, with the mean gene size for UVBi genes (approximately 30 kb) approximately 1.8 times smaller than that of the UVBr genes (approximately 54 kb) and a mean intron size for the UVBi genes (approximately 4,000 bp) approximately 1.5 times smaller than that of the UVBr genes (approximately 5,900 bp) (Figure 2). The mean number of introns per gene was also significantly smaller in the UVBi compared to the UVBr gene class (unpublished data). There were no differences in gene size, intron size, and the number of introns per gene between the set of UVBr genes and the entire set of approximately 35,000 genes on the array.

### Functional Annotation of UV-Responsive Genes by Over-Representation Analysis

**Figure 3:**
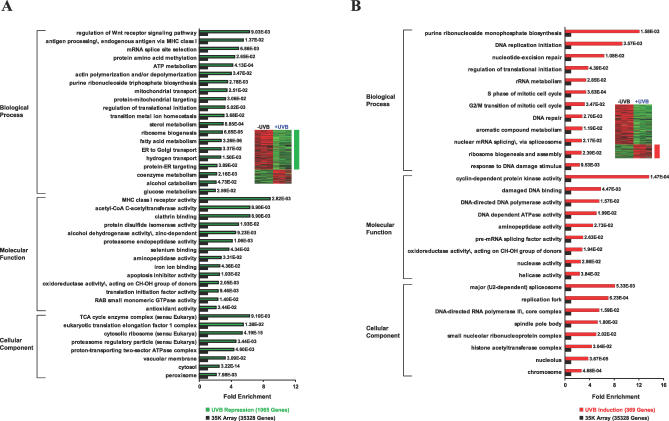
Over-Representation Analysis of Functional GO Terms for UVB-Responsive Genes in the Basal Epidermis of Normal (*+/+*) P2.5 Mice EASE software [48] was used to map GO terms to (A) 1,065 UVB-repressed (green bars) and (B) 369 UVB-induced (red bars) genes. GO terms that were significantly enriched (*p* = 0.05), by 2-fold, relative to all of the assayed genes on the 35,000 array (35,328 genes) (black bars) and that contained at least three gene hits, are displayed. GO terms are ranked, within high-level GO branches, by fold-enrichment relative to the 35,000 array. A thumbnail image provides a reference for each of the analyzed UVB response classes.

To better characterize the UVB transcriptional skin response in normal C57BL/6J mice, we extracted Gene Ontology (GO) terms from all UVBr or UVBi genes and compared the relative frequencies of these GO terms to those for all of the genes on the 35,000 array. Using EASE software [48], we observed 220 and 199 GO categories that were significantly enriched (*p* = 0.05) for the UVBr and UVBi genes, respectively. Of these, 119 UVBr and 64 UVBi categories were supported by at least three gene hits and were enriched by at least 2-fold over the 35,000 array. We focused on nonoverlapping biological process, molecular function, or cellular component terms for further study; results are presented in Figure 3.

For the 1,065 UVBr genes (Figure 3A), over-represented GO terms were largely consistent with those which we had observed in a previous study using basal epidermis [49]. Biological processes over-represented by UVBr genes in the basal epidermis included many metabolic and signaling functions: sterol and fatty acid metabolism, ATP metabolism, purine ribonucleoside triphosphate biosynthesis, regulation of Wnt receptor signaling pathway, antigen processing, endogenous antigen via MHC class I, and ribosome biogenesis. Molecular functions over-represented by UVBr genes in the basal epidermis largely corresponded to the aforementioned processes: MHC class I receptor activity, acetyl-coenzyme A C-acetyltransferase activity, clathrin binding, protein disulfide isomerase activity, selenium binding, and iron binding.

The pattern of GO terms over-represented for the 369 UVBi genes in the basal epidermis was very different from that of the UVBr genes (Figure 3B versus Figure 3A). Biological processes over-represented by UVBi genes included DNA repair (nucleotide-excision repair) and response to DNA damage stimulus, S-phase and G2/M transition of mitotic cell cycle, DNA replication initiation, rRNA metabolism, and nuclear mRNA splicing. Molecular functions over-represented by UVBi genes included nuclease and helicase activities, damaged DNA binding, pre-mRNA splicing factor activity, DNA-directed DNA polymerase activity, and cyclin-dependent protein kinase activity.

As described further below, UVB-responsive genes identified in our study and others exhibit only a partial overlap when considered from the perspective of individual genes, in part due to differences in array platforms and in part due to different patterns of gene expression between skin in vivo and cells in culture. However, large-scale transcriptional repression, downregulation of basal metabolic functions, upregulation of DNA repair functions, and bias in gene and intron size between these functional classes are general themes emerging from our analysis of the UVB response in vivo and are concordant with earlier work carried out on a variety of cultured cells and UV exposures.

### Genetic Deconvolution of Gene Expression in the Basal Epidermis: Melanocytes (Kit-Dependent) and Mc1r Signaling

To investigate how melanocytes and Mc1r signaling contribute to epidermal gene expression, we made use of two classic pigmentation mutants, *viable dominant spotting (Kit^W-v^/Kit^W-v^)* and *recessive yellow (Mc1r^e^/Mc1r^e^).* The *Kit^W-v^* allele is a missense mutation that severely impairs tyrosine kinase activity [50], yielding animals that, post embryonic day 12.5, are almost completely bereft of skin and hair melanoblasts [51]; the *Mc1r^e^* allele is a complete loss-of-function that causes almost exclusive production of pheomelanin instead of eumelanin [52]. Using *Mc1r^e^* and *Kit^W-v^* alleles on a congenic C57BL/6 background, we measured gene expression profiles in basal epidermis of P2.5 mutant animals and compared the results to those obtained from nonmutant C57BL/6 animals. Kit signaling is also required for development of germ cells, erythrocytes, and mast cells. However, pigment cell development is, in general, more sensitive to *Kit* gene dosage than mast cell development (*Kit^W-v^/Kit^W-v^* mutant mice retain approximately 40% of the normal number of mast cells), and mast cells are normally found in the dermis rather than the epidermis; therefore, we expected that epidermal gene expression in *Kit^W-v^/Kit^W-v^* mutant mice would be influenced mostly by deficiency of pigment cells.

**Figure 4:**
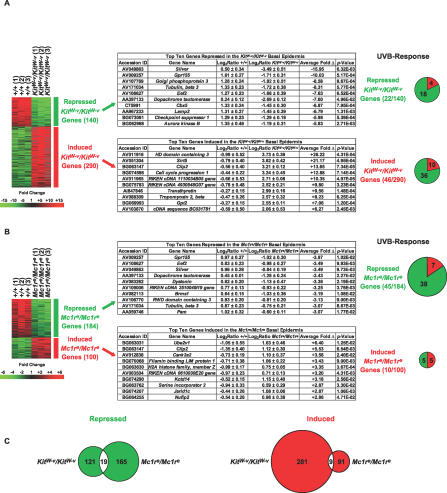
Proportion of Kit- and Mc1r-Dependent Genes Present in the General UVB Transcriptional Response in the Basal Epidermis of P2.5 Mice The ordered columns represent replicate samples derived from (A) *Kit^W-v^/Kit^W-v^* and (B) *Mc1r^e^/Mc1r^e^* mice compared to normal (*Mc1r^+^/Mc1r^+^*; *Kit^+^/Kit^+^*) mice, and the rows represent log_2_ ratios for differentially expressed genes. Data are displayed as a pseudocolor heatmap where red, green, and black represent induction, repression, and no change, respectively, relative to the median. Gray represents missing data. The ratio fold-change is indicated by a scale bar. Genes are ranked by their average fold change (Δ) value. The average fold Δ value was obtained by dividing the average expression level of each gene in replicate mutant (*Kit^W-v^/Kit^W-v^* or *Mc1r^e^/Mc1r^e^*) samples by that in the corresponding normal control (*Mc1r^+^/Mc1r^+^*; *Kit^+^/Kit^+^*) samples. Average expression levels for the mutant and control groups are represented as log_2_ ratios ±SEM. The top ten ranked repressed and induced genes are listed along with their corresponding average fold Δ and Student's *t*-test *p*-values. In some cases, official mouse gene symbols are used, instead of the lengthier gene names. The fraction of differentially expressed genes in each *Kit^W-v^/Kit^W-v^* or *Mc1r^e^/Mc1r^e^* class that is also UVB responsive is represented by a colored pie chart (green, repressed; red, induced), with the size of the pie chart proportional to the percentage of Kit- or Mc1r-dependent genes that are UVB responsive. The overlap between the total number of Kit- and Mc1r-dependent genes (containing all 430 *Kit^W-v^/Kit^W-v^* and 284 *Mc1r^e^/Mc1r^e^* differentially expressed genes that were initially detected, regardless of UVB responsiveness) is shown in (C) for each of the repressed (green) and induced (red) classes.

We first characterized patterns of gene expression in *Kit^W-v^/Kit^W-v^* and *Mc1r^e^/Mc1r^e^* animals in the absence of UV radiation. We identified 430 genes whose expression in the basal epidermis was altered in *Kit^W-v^/Kit^W-v^* animals: 140 downregulated and 290 upregulated (Figure 4A). Among the downregulated group, over-represented GO terms included melanin biosynthesis (48.7-fold enriched; *p* = 6.51E−04), protein and electron transport, adenyl and guanyl nucleotide binding, organelle organization, and biogenesis, as well as small GTPase-mediated signal transduction (Table S2). Among the upregulated group, over-represented GO terms included embryogenesis and morphogenesis, development, extracellular space, cell adhesion molecule, and signal transducer activity (Table S3). While some of the downregulated genes simply reflect the loss of melanocytes (and melanocyte-specific genes), most genes whose expression was altered in *Kit^W-v^/Kit^W-v^* animals are not melanocyte-specific. These results indicate that the gene expression profile in *Kit* mutant mice represents a transcriptional response rather than a simple change in cellular composition and suggest that, in addition to pigmentation, melanocytes influence the development and maintenance of the extracellular environment in the basal epidermis.

We also identified 284 genes whose expression was altered in *Mc1r^e^/Mc1r^e^* compared to nonmutant animals (in the absence of UV radiation): 184 downregulated and 100 upregulated (Figure 4B). Approximately 10% of the genes downregulated in *Mc1r^e^/Mc1r^e^* animals were also downregulated in *Kit^W-v^/Kit^W-v^* animals (Figure 4C), including two genes required for eumelanogenesis, Dopachrome tautomerase and *Silver*. However, there was virtually no overlap between Mc1r- and Kit-dependent genes when conditioning on UVB exposure (described further below); thus, Mc1r signaling is an important component in how the epidermis senses and responds to pigment cells in the basal state, but Mc1r signaling plays a specific and largely independent role from Kit (and the presence of melanocytes) in the transcriptional response to UV radiation.

We compared the profiles of Mc1r- and Kit-dependent genes in the basal epidermis with the 1,102 unique genes that were either induced or repressed by UVB in nonmutant skin as depicted in Figure 1. The proportion of UVB-sensitive genes in each of the four groups (induced or repressed in *Kit^W-v^/Kit^W-v^* or *Mc1r^e^/Mc1r^e^* animals) varied from 10% (induced in *Mc1r^e^/Mc1r^e^* animals) to 24% (repressed in *Mc1r^e^/Mc1r^e^* animals), with the same general theme described earlier—large-scale transcriptional repression of basal metabolic functions—apparent in each of the four groups (right side, Figure 4A and 4B).

### Distinct Signatures for Mc1r- and Melanocyte (Kit)-Dependent Gene Expression in the Response to UV Radiation

To investigate if melanocytes and Mc1r signaling are required specifically for the cutaneous response to UV radiation, we used the same UVB exposure paradigm described earlier and compared gene expression profiles in the basal epidermis of P2.5 *Kit^W-v^/Kit^W-v^* and *Mc1r^e^/Mc1r^e^* to that obtained from nonmutant animals. The total data set consisted of 18 arrays with three biological replicates per sample (three genotypes, either exposed or not exposed to UV radiation).

**Figure 5:**
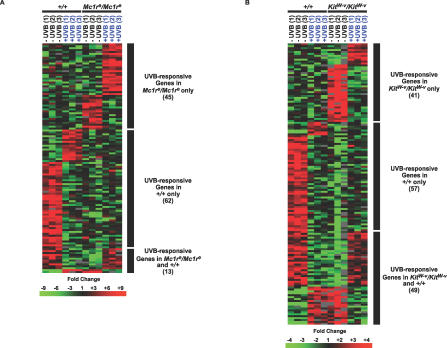
Heatmap of UVB × Pigmentation Genotype Interaction Genes in the Basal Epidermis of P2.5 Mice Two-way ANOVA tests, using 13,508 genes, were performed on *Mc1r^+^/Mc1r^+^* and *Mc1r^e^/Mc1r^e^* samples (−UVB and +UVB) and *Kit^+^/Kit^+^* and *Kit^W-v^/Kit^W-v^* (−UVB and +UVB) samples, yielding 120 Mc1r-dependent and 147 Kit-dependent UVB interacting genes. Post hoc analyses, using pairwise Student's *t*-tests, were used to reveal interactions between specific genotypes and UVB treatment. The ordered columns of the heatmap represent replicates of sham- (black) and UVB-irradiated (blue) basal epidermal samples derived from *+/+*, *Mc1r^e^/Mc1r^e^,* and *Kit^W-v^/Kit^W-v^* P2.5 mice. The genes are represented by rows and are arranged to illustrate interactions in the basal epidermis between UVB and *Mc1r* (A) and UVB and *Kit* (B). Red, green, and black indicate induction, repression, and no change, respectively, relative to the centered median. Gray represents missing data. The ratio fold-change is indicated by a scale bar.

We used two-way analysis of variance (ANOVA) to characterize the interaction between genotype and UVB exposure and identified 120 and 147 genes that showed a significant interaction (*p* = 0.05) between UVB and *Mc1r* and between UVB and *Kit,* respectively. Post hoc analyses performed separately on each of the 120 and 147 gene sets yielded three patterns, depending on whether a gene responded to UVB only in the mutant, only in the nonmutant, or in both mutant and nonmutant genotypes (Figure 5). Collectively, we refer to the 120 UVB × *Mc1r* genes as the Mc1r-dependent UVB-responsive genes (Table S4) and the 147 UVB × *Kit* genes as the Kit-dependent UVB-responsive genes (Table S5).

**Figure 6:**
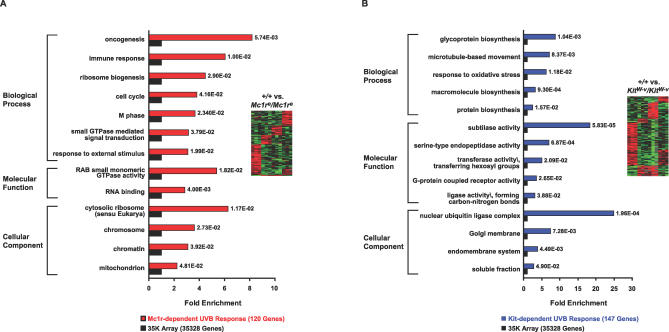
Over-Representation Analysis of Functional GO Terms for Mc1r- and Kit-Dependent UVB-Responsive Genes in the Basal Epidermis of P2.5 Mice GO terms, using EASE software [48], were mapped to (A) 120 Mc1r-dependent UVB-responsive genes (red bars) and (B) 147 Kit-dependent UVB-responsive genes (blue bars). GO terms that were significantly enriched (*p* = 0.05), by 2-fold, relative to all of the assayed genes on the 35,000 array (35,328 genes) (black bars) and that contained at least three gene hits, are displayed. GO terms are ranked, within high level GO branches, by fold-enrichment relative to the 35,000 array. A thumbnail image provides a reference for each of the analyzed UVB response classes.

Surprisingly, we found very little overlap between the Mc1r- and the Kit-dependent UVB-responsive genes (only five in common of a total of 267), suggesting that the biological processes engaged by Mc1r signaling in the response to UVB are distinct from the ability of pigment cells to protect against UV radiation by providing a simple physical barrier. To further explore this idea, we extracted and compared GO terms derived from the Mc1r- and the Kit-dependent UVB-responsive gene sets using the same approach described earlier (Figure 3). Significant (*p* = 0.05) GO terms that contained at least three hits and that were enriched by at least 2-fold over the 35,000 array are depicted in Figure 6 for both sets of genes. The Mc1r-dependent UVB response was characterized largely by genes involved in the cell cycle *(Cdc2l1, Fos, Hspa8, Mtcp1, Top3a,* and *2810406C15Rik)* and oncogenesis *(Cdc2l1*, *Fgfr1,* and *Mtcp1)* and included functions and components relating to GTPase-mediated signal transduction *(Grb2, Rab7, Rab12, Rab35,* and *Ralbp1),* chromatin *(H2faz*, *Scmh1,* and *5430405G24Rik),* ribosomes *(Rpl8*, *Rpl13,* and *Rps16),* and mitochondria *(Atpif1*, *C1qbp*, *Cndp2*, *Mtcp1*, *Pdha1,* and *Phb)*.

By contrast, the Kit-dependent UVB response was characterized largely by genes involved in glycoprotein biosynthesis *(C1galt1c1*, *Itm1*, *Man1a2,* and *Prkcsh)* and the response to oxidative stress *(Prdx5*, *Sepp1,* and *Stk25)* and included functions and components relating to protease activity *(Adcy7*, *Col6a1*, *Klk5*, *Hspb8,* and *Mbtps1)* and the Golgi transport system *(Inpp5e*, *Man1a2, Rer1*, *Ext1*, *Grb2*, *Inpp5e*, *Man1a2, Rer1,* and *Surf4).*

To better understand how the set of Mc1r-dependent UVB-responsive genes in mice might be related to *MC1R* genotype as a risk factor for human skin cancer, we considered Mc1r-dependent UVB-responsive genes known to have GO-annotated roles in the cell cycle (Table 1). In nearly every case, the direction in which expression of these genes was altered suggested that Mc1r signaling might facilitate UV-induced cell cycle arrest. For example, *Phb,* a negative regulator of the cell cycle, was induced by UVB in *Mc1r^+^/Mc1r^+^* animals but not in *Mc1r^e^/Mc1r^e^* animals. Similarly, *Bop1,* another negative regulator of the cell cycle, was repressed by UVB in *Mc1r^e^/Mc1r^e^* animals but not in *Mc1r^+^/Mc1r^+^* animals, while *Mark3* and *Cdc2l1,* both positive cell cycle regulators, were induced by UVB in *Mc1r^e^/Mc1r^e^* animals but not in *Mc1r^+^/Mc1r^+^* animals.

**Table 1:**
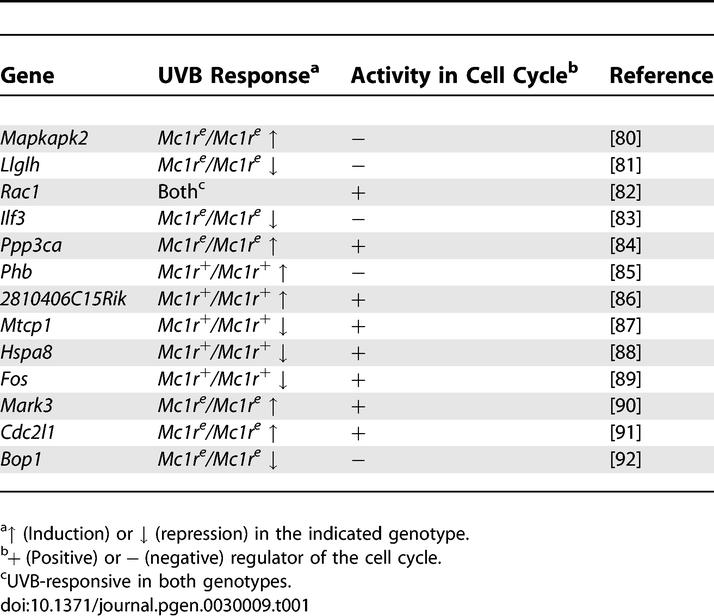
Mc1r-Dependent UVB-Responsive Genes Previously Reported to Regulate the Cell Cycle

We also considered whether any of the Mc1r-dependent UVB-responsive genes had previously been implicated in cancer biology by virtue of their aberrant regulation. Of 20 genes for which roles in cancer susceptibility or progression had previously been suggested (Table 2), 75% exhibited the direction of change expected for a protective Mc1r-dependent UVB response in the basal epidermis. For example, whereas *Arpc1b* is normally induced in the *Mc1r^+^/Mc1r^+^* basal epidermis, it remained unresponsive in *Mc1r^e^/Mc1r^e^* animals and is frequently silenced in gastric cancers [53]. In contrast, while *Mapkapk2* was normally unresponsive to UVB in the basal epidermis of *Mc1r^+^/Mc1r^+^* animals, it was induced in *Mc1r^e^/Mc1r^e^* animals and has been reported to be activated in some breast cancers [54].

**Table 2:**
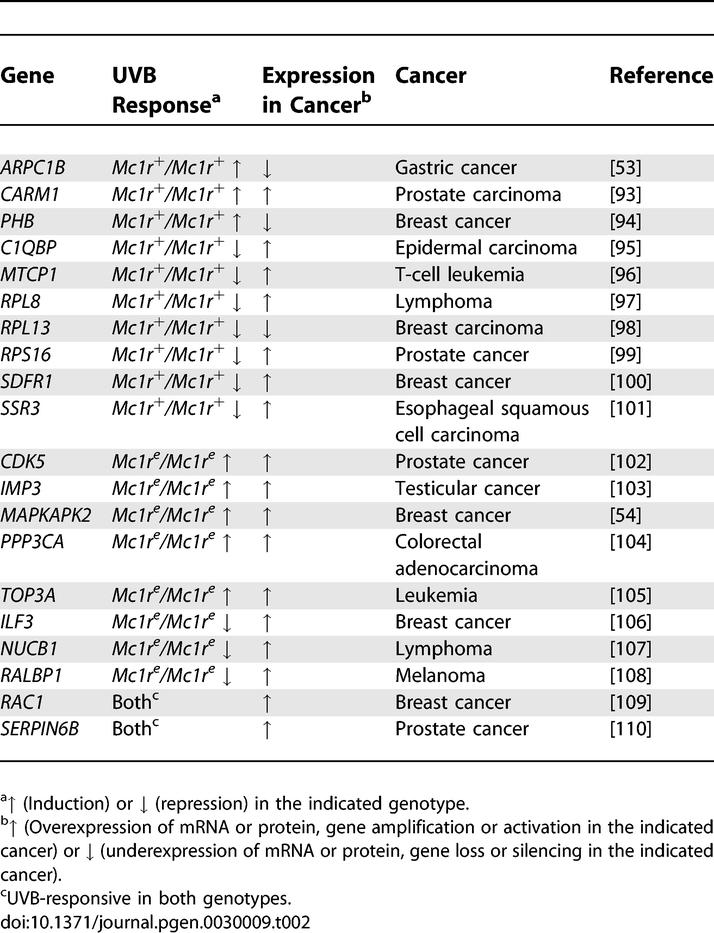
Mc1r-Dependent UVB-Responsive Genes Previously Reported to Be Aberrantly Regulated in Cancer Studies

### MC1R-Dependent UVB-Responsive Genes, Melanoma Profiling, and Cancer Biology

Given the over-representation of cell cycle and oncogenesis genes in our Mc1r-dependent UVB response, we sought to determine whether the expression patterns of these genes might be informative in a clinical setting. Among a number of previous microarray studies of human melanoma or melanoma cell lines, we identified two large data sets based on tissue samples from either primary melanoma [55] or melanoma metastases [56].

**Figure 7:**
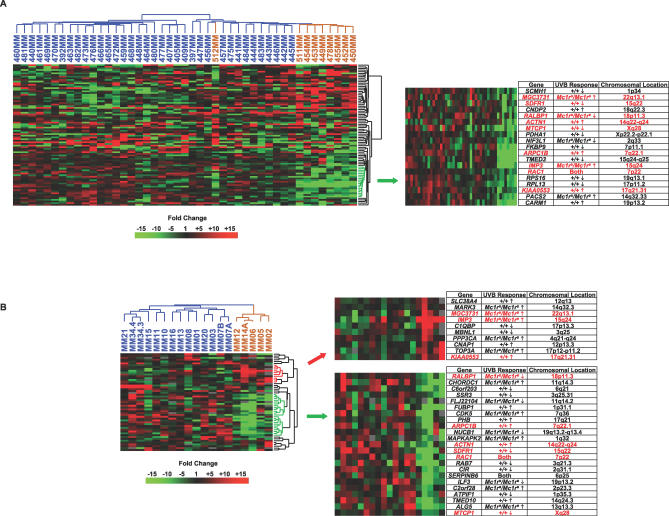
Unsupervised Hierarchical Clustering of Mc1r-Dependent UVB-Responsive Genes in Human Melanoma Samples Orthologous human gene expression data, derived from 120 mouse genes that were identified as Mc1r-dependent and UVB-responsive in the basal epidermis of P2.5 mice, were clustered in (A) a primary melanoma data set [55], here consisting of 86 genes and 45 samples, and (B) a melanoma metastases data set [56], here consisting of 53 genes and 19 samples. Melanoma subclusters correspond to the original subtypes, TNM stage I-II (blue) versus III-IV (orange) in (A), first identified by Talantov et al. [55], and growth phase type I radial (orange) versus type II vertical (blue) in (B), first identified by Haqq et al. [56]. Genes driving the observed melanoma clustering patterns are displayed to the right of each large cluster, along with their corresponding UVB response (↑, induction; ↓, repression; and Both, regulation in both *Mc1r^+^/Mc1r^+^* and *Mc1r^e^/Mc1r^e^*) in the *Mc1r^+^/Mc1r^+^* or *Mc1r^e^/Mc1r^e^* basal epidermis, as well as their chromosomal location. Genes highlighted in red are common to both primary and metastatic melanoma gene clusters. Red, green, and black indicate induction, repression, and no change, respectively, relative to the centered median. Gray represents missing data. The ratio fold-change is indicated by a scale bar.

Starting with our 120 Mc1r-dependent UVB-responsive genes, we were able to retrieve gene expression data for 86 and 53 orthologous human genes from the primary melanoma and the melanoma metastases data sets, respectively (see Materials and Methods for further details), of which approximately one third (47) were shared between the two data sets. Hierarchical clustering revealed that our Mc1r-dependent UVB-responsive genes were able to partition the primary melanoma (Figure 7A) and the melanoma metastases (Figure 7B) samples into two subtypes each. Both sets of melanoma subclusters obtained here were nearly identical to those originally identified, with the primary melanoma subtypes [55] corresponding to TNM stage (I-II versus III-IV), and the melanoma metastases subtypes [56] corresponding to growth phase (type I radial, in situ and minimally invasive tumors, versus type II vertical, aggressive metastatic tumors). Of the 86 genes tested on the primary melanoma data set, expression levels for 19 distinguished the different stages, with all of the 19 underexpressed in the stage III-IV samples relative to the stage I-II samples (Figure 7A). Of the 53 genes tested on the melanoma metastases data set, expression levels for 33 distinguished the different growth phases, with 10 and 23 genes overexpressed and underexpressed in the type I radial growth relative to the type II vertical growth samples, respectively (Figure 7B). Closer inspection of the overlap between the 19 and 33 genes used to distinguish the primary melanoma and melanoma metastases data sets revealed nine genes in common: *ACTN1, ARPC1B, IMP3, KIAA0553, MGC3731, MTCP1, RAC1, RALBP1,* and *SDFR1*.

### Validation, Comparison, and Context

The microarray platform and design used here are identical to those of a previous study in which we compared gene expression profiles in different skin layers of nonmutant and *Mc1r^e^/Mc1r^e^* mice and carried out selective validation by semiquantitative reverse-transcription polymerase chain reaction [49]; nearly all of the genes that we previously identified as Mc1r-dependent in the basal epidermis were also detected in the current set of experiments. To examine these findings in a broader context, we compared our UVB-responsive gene list to those reported in other large-scale UV expression experiments. From 28 published studies using a variety of different experimental designs and platforms (species, cell type, tissues, wavelength, dose, and time) (Table S6), we retrieved the UV-responsive gene lists and then combined and sorted these lists to yield a nonredundant set of 5,178 UniGene IDs. From the cDNAs shown to be UVB responsive in the basal epidermis (Figure 1 and Table S1), we identified a nonredundant set of 1,201 UniGene IDs. There are 502 genes in common between the two sets (5,178 from 28 earlier studies and the 1,201 reported here); this degree of overlap is highly significant (*p* < 0.0001), yet indicates that our approach yielded a large number of genes (699) (Table S7) not previously known to be UV responsive.

## Discussion

Of 28 previous studies designed to study the large-scale transcriptional response to UV radiation, only three examined tissues or tissue compartments; the remainder focused on cultured cells. In two studies from Hochberg and colleagues [57,58] where the UV response of human suprabasal epidermis (obtained from suction blisters) was compared directly to that of cultured human keratinocytes using the same platform, only 207 of 1,931 genes found to be UV responsive in either the suprabasal epidermis or cultured keratinocytes were UV responsive in both samples.

Our findings provide additional support for the notion that substantial differences exist between the identities of UV-responsive genes in tissues and cultured cells, yet reinforce several common themes. First, transcriptional repression accounts for a large proportion (75%) of the UV response, a finding consistent with that seen, although not always reported, in other large-scale UV gene expression studies. Second, downregulation of basal metabolic genes together with upregulation of DNA damage and/or repair genes characterizes the UV transcriptional response across a broad set of cell types and several tissues. Finally, the quantitative characteristics of the UV response are characterized by small changes in expression that affect a large number of genes. All of these features may be linked to the observation made here and by McKay et al. [47] that UV-induced and UV-repressed genes differ not only in their biological function but also in their size, suggesting evolutionary pressure to ensure that the very genes required for a critical response to UV radiation are themselves less likely to sustain UV-induced DNA lesions.

**Figure 8:**
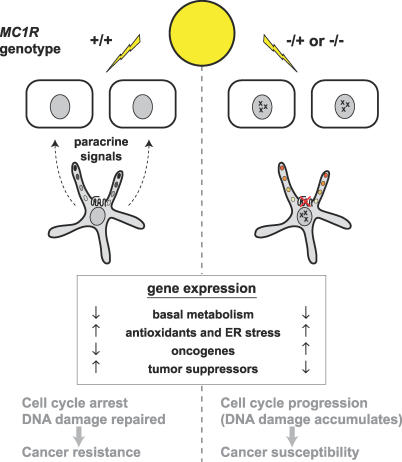
Model of MC1R-Dependent Protective Mechanisms against UVB Radiation in the Basal Epidermis Upon UVB-induced stress, the basal epidermis mounts a general protective response which is characterized by large-scale transcriptional repression of basal metabolic genes and induction of cell cycle and DNA-damage repair genes. Kit-dependent signals mediate the upregulation of antioxidant and ER stress-response genes. In homozygous wild-type MC1R individuals, paracrine factors released by melanocytes mediate cell cycle arrest in order to repair UVB-induced DNA damage (suppression and induction of oncogene and tumor suppressor gene expression, respectively). In contrast, partial or complete loss of MC1R function in the basal epidermis may predispose individuals to accumulating UVB-induced photolesions (x), through a diminished capacity of the basal epidermis to elicit an appropriate cell cycle arrest or DNA-damage repair [67] response (induction and suppression of oncogene and tumor suppressor gene expression, respectively), thereby rendering them susceptible to skin cancer.

In addition to providing a broader perspective on the transcriptional response to UV radiation, our work helps to explain the epidemiologic relationship between *MC1R* genotype and skin cancer susceptibility. The set of Mc1r-dependent UVB-responsive genes we identified is greatly enriched for processes and functions involved in oncogenesis and the cell cycle; moreover, the pattern in which specific genes change suggests that a functional Mc1r contributes to the ability of UVB to induce cell cycle arrest in the basal epidermis (Figure 8). Our work on the genome-level response to UV radiation was necessarily carried out in laboratory mice, where the availability of specific mutations on a defined genetic background allows a substantial degree of experimental control. However, our conclusions about the general relationship between *MC1R* genotype and cancer susceptibility in the mouse are supported by previous studies of two specific melanoma genes in humans: *CDKN2A* (a tumor suppressor) and *BRAF* (a proto-oncogene). In families carrying a germline *CDKN2A* mutation, the risk of melanoma was substantially increased by the presence of an *MC1R* variant [59–63]. In recent work from Landi et al. [64], germline *MC1R* variation was found to be a risk factor for developing melanomas with *BRAF* mutations. Because the association between *MC1R* variation and *BRAF* was most apparent in melanomas without evidence of chronic sun damage, Landi et al. hypothesized that UV radiation acted indirectly to promote BRAF-mutant melanoma, which is also consistent with our findings.

The nature of our experimental system—an integrated tissue collected from whole animals—makes it difficult to assess whether Mc1r signaling might affect UVB-induced cell cycle arrest in keratinocytes, melanocytes, or both. However, in recent work from Yamaguchi et al. [65] where the effects of UV radiation on cutaneous DNA damage and cell death were examined in individuals with different pigmentary phenotypes, light skin was found to exhibit a surprising lack of UV-induced apoptosis in many cells of the basal epidermis. Although *MC1R* genotypes were not determined in the subjects studied by Yamaguchi et al. [65], our work suggests that some of the effects they observed might be attributed to *MC1R* heterozygosity.

What sort of molecular mechanisms might link MC1R signaling to UV-induced apoptosis [39–41]? From a cell-autonomous perspective, phosphorylation of p53 Ser389 is a critical and major step in triggering apoptosis in response to UV-induced DNA damage [66], and it is possible that MC1R activation (either constitutive or in response to UV radiation) plays a permissive role. However, recent studies by Abdel-Malek and colleagues [67] indicate that *MC1R* deficiency causes increased UV-induced apoptosis in cultured melanocytes. Thus, a more likely scenario is that decreased MC1R signaling in melanocytes exposed to UV radiation in vivo impairs the ability of surrounding cells to undergo p53-mediated cell cycle arrest and apoptosis. Indeed, previous work from our group and others indicates that while *Mc1r* expression is confined mainly to melanocytes, *Mc1r* deficiency leads to substantial changes in gene expression throughout the skin and suggests that melanocytes influence the behavior of surrounding cells via paracrine mechanisms. For example, L-type prostaglandin D2 synthase *(Ptgds)* is expressed at high levels in pigment cells [68] and is repressed in *Mc1r^e^/Mc1r^e^* skin (current data, [49]); prostaglandin D2 and its derivatives could be one of several mediators released from Mc1r-competent melanocytes that facilitate or potentiate p53 phosphorylation in surrounding cells [69]. Regardless of the molecular mechanisms that link Mc1r signaling to changes in the expression of cell cycle genes, our analysis of human melanoma samples indicates that those genes are relevant to melanoma biology, since our set of Mc1r-dependent UVB-responsive genes discriminates between the severity of primary melanoma subtypes and between the growth phases of melanoma metastasis subtypes.

Several known antioxidant genes, including *Glo1, Prdx5, Sepp1,* and *Stk25,* were all repressed in basal epidermis of *Kit^+^/Kit^+^* animals but not *Kit^W-v^/Kit^W-v^* animals. These results are consistent with reports in which UVB, in inducing oxidative stress, was reported to deplete the antioxidant defense system in the epidermis of mice [70,71] and humans [72]. We also identified additional genes, within the Kit-dependent UVB response, that were not annotated in the GO database as oxidative or endoplasmic reticulum (ER) stress response genes, including *Cdk5, Eif1, Egln1, Ext1, Gpr175, Gpt2, Itm1, Nars, Pabpc1, Pecam1, Prdx4, Prkrip1, Pten, Rheb, Sh3glb1, Tbc1d20,* and *Tuba3,* for which roles in oxidative stress/hypoxia and the ER stress/unfolded protein response have been reported [73–76]. Several other genes involved in the ER/Golgi trafficking or folding of proteins, but not previously reported to be regulated during oxidative stress/hypoxia or the unfolded protein response, were also identified, including *Ext1, Ergic3, Grb2, Inpp5e, Man1a2, Prkcsh, Rer1,* and *Surf4,* raising the possibility that these genes may represent new skin stress response genes. Given the absence of this oxidative/ER stress response signature in our Mc1r-dependent UVB response, we interpret our findings to suggest that melanocytes, despite constituting a small fraction of the skin cell population, play a major role during oxidative and ER stress in the basal epidermis and, significantly, that this role is independent of Mc1r signaling.

Taken together, our results provide a molecular framework for the underlying mechanisms by which melanocytes and the Mc1r independently mediate, and afford protection against, UVB signals within the normal skin environment. This latter finding is of particular significance as it suggests insights into the molecular mechanisms by which *MC1R* mutations predispose toward melanoma susceptibility.

## Materials and Methods

### Mice and UVB irradiation.

C57BL/6J mice of three different pigmentation genotypes—*Mc1r^+^/Mc1r^+^; Kit^+^/Kit^+^, Mc1r^e^/Mc1r^e^; Kit^+^/Kit^+^,* and *Mc1r^+^/Mc1r^+^; Kit^W-v^/Kit^W-v^*—were obtained from The Jackson Laboratory (Bar Harbor, Maine, United States). P1.5 pups were immobilized (ventrum side down) with double-sided adhesive tape on an inverted plastic mouse cage. The dorsums of the pups were irradiated with a single dose of 100 mJ/cm^2^ UVB, using a Stratalinker 2400 UV crosslinker (Stratagene, http://www.stratagene.com), equipped with five 15-W bulbs with a peak emission wavelength of 312 nm. The irradiance of the bulbs was measured with an International Light 1400 Radiometer (International Light, http://www.intl-light.com). The administered UVB dose corresponded to a mild sunburn dose, approximately one minimal erythemal dose, for the C57BL/6J strain [77]. Control pups from all three genotypes were shielded from UV irradiation with a black Perspex box. Immediately postirradiation, all pups were returned to their mothers for 24 h, after which they were killed. Because *Kit^W-v^/Kit^W-v^* mice are sterile, homozygous neonates were obtained by heterozygous matings followed by a polymerase chain reaction and NciI restriction digest–based diagnostic genotyping assay for the *Kit^W-v^* mutation [78]. Dorsal trunk skins were dissected and basal epidermal cells were prepared and stored as previously described [49].

### Experimental design.

The methodology described here conforms to Minimum Information About a Microarray Experiment (MIAME) standards (http://www.mged.org/Workgroups/MIAME/miame.html). Gene expression patterns were studied in the basal epidermis of neonatal mice by comparing three groups of animals *(Mc1r^+^/Mc1r^+^; Kit^+^/Kit^+^, Mc1r^e^/Mc1r^e^; Kit^+^/Kit^+^,* and *Mc1r^+^/Mc1r^+^; Kit^W-v^/Kit^W-v^)* under two conditions (−UVB and +UVB). We thus studied six groups altogether, with three biological replicates per group. Each basal epidermal replicate was derived from a separate pool containing between two and four neonatal littermates. We used a common reference experimental design in which each basal epidermal sample was hybridized together with a common RNA reference pool. This reference pool has been previously described [49] and allowed us to easily perform comparisons across all of our basal epidermal samples.

### Microarray design.

The cDNA microarrays used in this study were generated at Stanford University, Stanford Functional Genomics Facility (http://www.microarray.org), using standard protocols (http://cmgm.stanford.edu/pbrown/protocols/index.html). Briefly, 35,328 cDNA clones, representing 14,605 unique mouse transcripts (UniGene Build 148), were obtained from the following five sources: 16,896 clones from RIKEN (http://fantom.gsc.riken.jp), 15,264 clones from the National Institute on Aging (http://lgsun.grc.nia.nih.gov/cDNA/15k.html), 1,632 I.M.A.G.E. clones from Invitrogen/Research Genetics (http://www.invitrogen.com), 960 clones from the Brain Molecular Anatomy Project (http://trans.nih.gov/bmap/resources/resources.htm), and 576 hand-selected clones from Stanford University.

### RNA extraction and labeling.

Total RNAs were extracted from the basal epidermal cells using TRIzol Reagent (Invitrogen) followed by further purification using a RNeasy cleanup protocol (Qiagen, http://www.qiagen.com). Experimental and reference RNA samples (20 μg each) were reverse-transcribed and then directly labeled using an anchored oligo-dT_18_ primer (Qiagen) and a CyScribe First-Strand cDNA Labeling Kit (Amersham Biosciences, http://www.amershambiosciences.com), as per the manufacturer's instructions. The RNA was degraded in 1N NaOH for 30 min at 70 °C followed by neutralization in 1N HCl. The Cy5-dUTP–labeled basal epidermal and Cy3-dUTP–labeled reference samples were combined, concentrated using Centricon YM-30 microconcentrators (Millipore/Amicon, http://www.millipore.com), and then competitively hybridized to a 35,000 spotted glass mouse microarray slide.

### Microarray hybridizations.

Hybridizations were performed in 32-μl reaction volumes, as previously described [49] in 3.4× standard sodium citrate (SSC), 0.3% sodium dodecyl sulfate, containing 1 μg/μl mouse *Cot-1* DNA (Invitrogen), 10 μg/μl yeast tRNA (Invitrogen), and 10 μg/μl synthetic poly(A)+ RNA (Sigma-Aldrich, http://www.sigmaaldrich.com), at 65 °C for 16 h, using custom-designed humidified hybridization chambers (Die-Tech, http://www.die-technology.com). Posthybridization washes were done for 2 min in each of 2× SSC, 0.03% sodium dodecyl sulfate, 2× SSC, 1× SSC, and 0.1× SSC. All washes were performed at room temperature. Slides were dried by centrifugation for 10 min at 600 rpm immediately prior to scanning.

### Data processing and statistical analyses.

Images were acquired with a GenePix 4000A scanner and feature measurements were extracted using GenePix Pro 3.0 software (Axon Instruments, http://www.axon.com). Raw data were uploaded into the Stanford Microarray Database (publicly available at http://smd.stanford.edu) where background-subtracted fluorescence ratios were linear-normalized such that the mean Cy5/Cy3 ratio on each array was 1. Good-quality spots were selected by manually excluding spots that were irregularly shaped or of small diameter or contained excessive background intensities. We next selected spots whose median signal intensity was 1.5-fold above the median background for both Cy3 and Cy5 channels. Our normalized, background-subtracted, raw data set totaled 31,756 spots. For our statistical analyses, the raw data set was filtered such that measurements were present in at least two of three replicates per experimental condition. This filter reduced our data set to 13,508 cDNAs (9,110 unique genes). Student's *t*- and two-way ANOVA tests were performed using GeneSpring Software (Agilent, Palo Alto, California, United States) on centered data sets, in which the median log_2_ ratio for each gene equaled 0. Over-representation analysis was performed using EASE [48], which reports functional gene categories as statistically significant GO terms. Significance values were adjusted for multiple hypothesis testing by the FDR, and values with an FDR of 0.05 were selected for further analyses.

### Unsupervised hierarchical clustering of melanoma data sets.

Using our list of 120 Mc1r-dependent UVB-responsive mouse genes, together with National Center for Biotechnology Information's HomoloGene and BLASTn algorithms, we retrieved gene expression data for 147 and 74 orthologous human genes from the primary melanoma and melanoma metastases data sets of Talantov et al. [55] and Haqq et al. [56], respectively. The log_2_ ratios for both data sets were median-centered separately, and nonredundant human gene lists for each of the data sets were generated by averaging expression data for gene replicates. The resulting primary melanoma data set, now consisting of 86 genes and 45 samples, and the melanoma metastases data set, now consisting of 53 genes and 19 samples, were clustered separately using an unsupervised algorithm in Cluster [79], in which uncentered correlation coefficients (similarity metric) for both samples and genes underwent average linkage clustering. The results are displayed as heatmaps using Treeview software [79].

## Supporting Information

Table S1 UVB-Responsive Genes in the *+/+* Basal Epidermis of P2.5 Mice (684 KB PDF)

Table S2 Enriched GO Terms for Downregulated Genes in the Basal Epidermis of P2.5 *Kit^W-v^/Kit^W-v^* Mice (67 KB PDF)

Table S3 Enriched GO Terms for Upregulated Genes in the Basal Epidermis of P2.5 *Kit^W-v^/Kit^W-v^* Mice (68 KB PDF)

Table S4 Mc1r-Dependent UVB-Responsive Genes in the Basal Epidermis of P2.5 Mice (50 KB PDF)

Table S5 Kit-Dependent UVB-Responsive Genes in the Basal Epidermis of P2.5 Mice (58 KB PDF)

Table S6 Summary of Large-Scale Gene Expression Studies that Have Identified UV-Responsive Genes (89 KB PDF)

Table S7 Nonoverlapping UVB-Responsive Genes in the *+/+* Basal Epidermis of P2.5 Mice (212 KB PDF)

## Abbreviations

ANOVA: analysis of variance
ER: endoplasmic reticulum
FDR: false discovery rate
GO: Gene Ontology
Mc1r or MC1R: melanocortin 1 receptor
P: postnatal day
SSC: standard sodium citrate
UV: ultraviolet
UVBi: ultraviolet B–induced
UVBr: ultraviolet B–repressed

## References

[pgen-0030009-b001] Hussein MR (2005). Ultraviolet radiation and skin cancer: Molecular mechanisms. J Cutan Pathol.

[pgen-0030009-b002] Norval M (2006). The mechanisms and consequences of ultraviolet-induced immunosuppression. Prog Biophys Mol Biol.

[pgen-0030009-b003] Kullavanijaya P, Lim HW (2005). Photoprotection. J Am Acad Dermatol.

[pgen-0030009-b004] Szabo G, Gerald AB, Pathak MA, Fitzpatrick TB (1969). Racial differences in the fate of melanosomes in human epidermis. Nature.

[pgen-0030009-b005] Thong HY, Jee SH, Sun CC, Boissy RE (2003). The patterns of melanosome distribution in keratinocytes of human skin as one determining factor of skin colour. Br J Dermatol.

[pgen-0030009-b006] Hennessy A, Oh C, Diffey B, Wakamatsu K, Ito S (2005). Eumelanin and pheomelanin concentrations in human epidermis before and after UVB irradiation. Pigment Cell Res.

[pgen-0030009-b007] Wakamatsu K, Kavanagh R, Kadekaro AL, Terzieva S, Sturm RA (2006). Diversity of pigmentation in cultured human melanocytes is due to differences in the type as well as quantity of melanin. Pigment Cell Res.

[pgen-0030009-b008] Valverde P, Healy E, Jackson I, Rees JL, Thody AJ (1995). Variants of the melanocyte-stimulating hormone receptor gene are associated with red hair and fair skin in humans. Nat Genet.

[pgen-0030009-b009] Sturm RA, Box NF, Ramsay M (1998). Human pigmentation genetics: The difference is only skin deep. Bioessays.

[pgen-0030009-b010] Rees JL (2000). The melanocortin 1 receptor (MC1R): More than just red hair. Pigment Cell Res.

[pgen-0030009-b011] Rees JL, Birch-Machin M, Flanagan N, Healy E, Phillips S (1999). Genetic studies of the human melanocortin-1 receptor. Ann N Y Acad Sci.

[pgen-0030009-b012] Flanagan N, Healy E, Ray A, Philips S, Todd C (2000). Pleiotropic effects of the melanocortin 1 receptor (MC1R) gene on human pigmentation. Hum Mol Genet.

[pgen-0030009-b013] Sturm RA (2002). Skin colour and skin cancer—MC1R, the genetic link. Melanoma Res.

[pgen-0030009-b014] Makova K, Norton H (2005). Worldwide polymorphism at the MC1R locus and normal pigmentation variation in humans. Peptides.

[pgen-0030009-b015] Wong TH, Rees JL (2005). The relation between melanocortin 1 receptor (MC1R) variation and the generation of phenotypic diversity in the cutaneous response to ultraviolet radiation. Peptides.

[pgen-0030009-b016] Garcia-Borron JC, Sanchez-Laorden BL, Jimenez-Cervantes C (2005). Melanocortin-1 receptor structure and functional regulation. Pigment Cell Res.

[pgen-0030009-b017] Bastiaens M, ter Huurne J, Gruis N, Bergman W, Westendorp R (2001). The melanocortin-1-receptor gene is the major freckle gene. Hum Mol Genet.

[pgen-0030009-b018] Leonard JH, Marks LH, Chen W, Cook AL, Boyle GM (2003). Screening of human primary melanocytes of defined melanocortin-1 receptor genotype: Pigmentation marker, ultrastructural and UV-survival studies. Pigment Cell Res.

[pgen-0030009-b019] Ringholm A, Klovins J, Rudzish R, Phillips S, Rees JL (2004). Pharmacological characterization of loss of function mutations of the human melanocortin 1 receptor that are associated with red hair. J Invest Dermatol.

[pgen-0030009-b020] Newton RA, Smit SE, Barnes CC, Pedley J, Parsons PG (2005). Activation of the cAMP pathway by variant human MC1R alleles expressed in HEK and in melanoma cells. Peptides.

[pgen-0030009-b021] Sanchez-Laorden BL, Sanchez-Mas J, Martinez-Alonso E, Martinez-Menarguez JA, Garcia-Borron JC (2006). Dimerization of the human melanocortin 1 receptor: Functional consequences and dominant-negative effects. J Invest Dermatol.

[pgen-0030009-b022] Palmer JS, Duffy DL, Box NF, Aitken JF, O'Gorman LE (2000). Melanocortin-1 receptor polymorphisms and risk of melanoma: Is the association explained solely by pigmentation phenotype?. Am J Hum Genet.

[pgen-0030009-b023] Box NF, Duffy DL, Irving RE, Russell A, Chen W (2001). Melanocortin-1 receptor genotype is a risk factor for basal and squamous cell carcinoma. J Invest Dermatol.

[pgen-0030009-b024] Pastorino L, Cusano R, Bruno W, Lantieri F, Origone P (2004). Novel MC1R variants in Ligurian melanoma patients and controls. Hum Mutat.

[pgen-0030009-b025] Landi MT, Kanetsky PA, Tsang S, Gold B, Munroe D (2005). MC1R, ASIP, and DNA repair in sporadic and familial melanoma in a Mediterranean population. J Natl Cancer Inst.

[pgen-0030009-b026] Stratigos AJ, Dimisianos G, Nikolaou V, Poulou M, Sypsa V (2006). Melanocortin receptor-1 gene polymorphisms and the risk of cutaneous melanoma in a low-risk southern European population. J Invest Dermatol.

[pgen-0030009-b027] Valverde P, Healy E, Sikkink S, Haldane F, Thody AJ (1996). The Asp84Glu variant of the melanocortin 1 receptor (MC1R) is associated with melanoma. Hum Mol Genet.

[pgen-0030009-b028] Smith R, Healy E, Siddiqui S, Flanagan N, Steijlen PM (1998). Melanocortin 1 receptor variants in an Irish population. J Invest Dermatol.

[pgen-0030009-b029] Vincensi MR, d'Ischia M, Napolitano A, Procaccini EM, Riccio G (1998). Phaeomelanin versus eumelanin as a chemical indicator of ultraviolet sensitivity in fair-skinned subjects at high risk for melanoma: A pilot study. Melanoma Res.

[pgen-0030009-b030] Wenczl E, Van der Schans GP, Roza L, Kolb RM, Timmerman AJ (1998). (Pheo)melanin photosensitizes UVA-induced DNA damage in cultured human melanocytes. J Invest Dermatol.

[pgen-0030009-b031] Chedekel MR, Smith SK, Post PW, Pokora A, Vessell DL (1978). Photodestruction of pheomelanin: Role of oxygen. Proc Natl Acad Sci U S A.

[pgen-0030009-b032] Maresca V, Flori E, Briganti S, Camera E, Cario-Andre M (2006). UVA-induced modification of catalase charge properties in the epidermis is correlated with the skin phototype. J Invest Dermatol.

[pgen-0030009-b033] Ye T, Hong L, Garguilo J, Pawlak A, Edwards GS (2006). Photoionization thresholds of melanins obtained free-electron laser photoelectron emission microscopy, femtosecond transient absorption spectroscopy, and EPR measurements of oxygen photoconsumption. Photochem Photobiol.

[pgen-0030009-b034] Menon IA, Persad S, Ranadive NS, Haberman HF (1983). Effects of ultraviolet-visible irradiation in the presence of melanin isolated from human black or red hair upon Ehrlich ascites carcinoma cells. Cancer Res.

[pgen-0030009-b035] Takeuchi S, Zhang W, Wakamatsu K, Ito S, Hearing VJ (2004). Melanin acts as a potent UVB photosensitizer to cause an atypical mode of cell death in murine skin. Proc Natl Acad Sci U S A.

[pgen-0030009-b036] Bolognia J, Murray M, Pawelek J (1989). UVB-induced melanogenesis may be mediated through the MSH-receptor system. J Invest Dermatol.

[pgen-0030009-b037] Sturm RA (1998). Human pigmentation genes and their response to solar UV radiation. Mutat Res.

[pgen-0030009-b038] Abdel-Malek Z, Suzuki I, Tada A, Im S, Akcali C (1999). The melanocortin-1 receptor and human pigmentation. Ann N Y Acad Sci.

[pgen-0030009-b039] Scott MC, Wakamatsu K, Ito S, Kadekaro AL, Kobayashi N (2002). Human melanocortin 1 receptor variants, receptor function and melanocyte response to UV radiation. J Cell Sci.

[pgen-0030009-b040] Bohm M, Wolff I, Scholzen TE, Robinson SJ, Healy E (2005). α-Melanocyte-stimulating hormone protects from ultraviolet radiation-induced apoptosis and DNA damage. J Biol Chem.

[pgen-0030009-b041] Kadekaro AL, Kavanagh R, Kanto H, Terzieva S, Hauser J (2005). α-Melanocortin and endothelin-1 activate antiapoptotic pathways and reduce DNA damage in human melanocytes. Cancer Res.

[pgen-0030009-b042] Hirobe T (1978). Stimulation of dendritogenesis in the epidermal melanocytes of newborn mice by melanocyte-stimulating hormone. J Cell Sci.

[pgen-0030009-b043] Hirobe T (1984). Histochemical survey of the distribution of the epidermal melanoblasts and melanocytes in the mouse during fetal and postnatal periods. Anat Rec.

[pgen-0030009-b044] Weiss LW, Zelickson AS (1975). Embryology of the epidermis: Ultrastructural aspects. III. Maturation and primary appearance of dendritic cells in the mouse with mammalian comparisons. Acta Derm Venereol.

[pgen-0030009-b045] Benjamini Y, Hochberg Y (1995). Controlling the false discovery rate: A practical and powerful approach to multiple testing. J R Stat Soc Ser B.

[pgen-0030009-b046] Yang G, Zhang G, Pittelkow MR, Ramoni M, Tsao H (2006). Expression profiling of UVB response in melanocytes identifies a set of p53-target genes. J Invest Dermatol.

[pgen-0030009-b047] McKay BC, Stubbert LJ, Fowler CC, Smith JM, Cardamore RA (2004). Regulation of ultraviolet light-induced gene expression by gene size. Proc Natl Acad Sci U S A.

[pgen-0030009-b048] Hosack DA, Dennis G, Sherman BT, Lane HC, Lempicki RA (2003). Identifying biological themes within lists of genes with EASE. Genome Biol.

[pgen-0030009-b049] April CS, Barsh GS (2006). Skin layer-specific transcriptional profiles in normal and recessive yellow (Mc1re/Mc1re) mice. Pigment Cell Res.

[pgen-0030009-b050] Nocka K, Tan JC, Chiu E, Chu TY, Ray P (1990). Molecular bases of dominant negative and loss of function mutations at the murine c-kit/white spotting locus: W37, Wv, W41 and W. EMBO J.

[pgen-0030009-b051] Mackenzie MA, Jordan SA, Budd PS, Jackson IJ (1997). Activation of the receptor tyrosine kinase Kit is required for the proliferation of melanoblasts in the mouse embryo. Dev Biol.

[pgen-0030009-b052] Robbins LS, Nadeau JH, Johnson KR, Kelly MA, Roselli-Rehfuss L (1993). Pigmentation phenotypes of variant extension locus alleles result from point mutations that alter MSH receptor function. Cell.

[pgen-0030009-b053] Kaneda A, Kaminishi M, Yanagihara K, Sugimura T, Ushijima T (2002). Identification of silencing of nine genes in human gastric cancers. Cancer Res.

[pgen-0030009-b054] Han Q, Leng J, Bian D, Mahanivong C, Carpenter KA (2002). Rac1-MKK3-p38-MAPKAPK2 pathway promotes urokinase plasminogen activator mRNA stability in invasive breast cancer cells. J Biol Chem.

[pgen-0030009-b055] Talantov D, Mazumder A, Yu JX, Briggs T, Jiang Y (2005). Novel genes associated with malignant melanoma but not benign melanocytic lesions. Clin Cancer Res.

[pgen-0030009-b056] Haqq C, Nosrati M, Sudilovsky D, Crothers J, Khodabakhsh D (2005). The gene expression signatures of melanoma progression. Proc Natl Acad Sci U S A.

[pgen-0030009-b057] Enk CD, Shahar I, Amariglio N, Rechavi G, Kaminski N (2004). Gene expression profiling of in vivo UVB-irradiated human epidermis. Photodermatol Photoimmunol Photomed.

[pgen-0030009-b058] Enk CD, Jacob-Hirsch J, Gal H, Verbovetski I, Amariglio N (2006). The UVB-induced gene expression profile of human epidermis in vivo is different from that of cultured keratinocytes. Oncogene.

[pgen-0030009-b059] Chaudru V, Laud K, Avril MF, Miniere A, Chompret A (2005). Melanocortin-1 receptor (MC1R) gene variants and dysplastic nevi modify penetrance of CDKN2A mutations in French melanoma-prone pedigrees. Cancer Epidemiol Biomarkers Prev.

[pgen-0030009-b060] Box NF, Duffy DL, Chen W, Stark M, Martin NG (2001). MC1R genotype modifies risk of melanoma in families segregating CDKN2A mutations. Am J Hum Genet.

[pgen-0030009-b061] Goldstein AM, Landi MT, Tsang S, Fraser MC, Munroe DJ (2005). Association of MC1R variants and risk of melanoma in melanoma-prone families with CDKN2A mutations. Cancer Epidemiol Biomarkers Prev.

[pgen-0030009-b062] Peris K, Fargnoli MC, Pacifico A, Surrenti T, Stolz W (2004). CDKN2A and MC1R mutations in patients with sporadic multiple primary melanoma. J Invest Dermatol.

[pgen-0030009-b063] van der Velden PA, Sandkuijl LA, Bergman W, Pavel S, van Mourik L (2001). Melanocortin-1 receptor variant R151C modifies melanoma risk in Dutch families with melanoma. Am J Hum Genet.

[pgen-0030009-b064] Landi MT, Bauer J, Pfeiffer RM, Elder DE, Hulley B (2006). MC1R germline variants confer risk for BRAF-mutant melanoma. Science.

[pgen-0030009-b065] Yamaguchi Y, Takahashi K, Zmudzka BZ, Kornhauser A, Miller SA (2006). Human skin responses to UV radiation: Pigment in the upper epidermis protects against DNA damage in the lower epidermis and facilitates apoptosis. FASEB J.

[pgen-0030009-b066] Bruins W, Zwart E, Attardi LD, Iwakuma T, Hoogervorst EM (2004). Increased sensitivity to UV radiation in mice with a p53 point mutation at Ser389. Mol Cell Biol.

[pgen-0030009-b067] Hauser JE, Kadekaro AL, Kavanagh RJ, Wakamatsu K, Terzieva S (2006). Melanin content and MC1R function independently affect UVR-induced DNA damage in cultured human melanocytes. Pigment Cell Res.

[pgen-0030009-b068] Takeda K, Yokoyama S, Aburatani H, Masuda T, Han F (2006). Lipocalin-type prostaglandin D synthase as a melanocyte marker regulated by MITF. Biochem Biophys Res Commun.

[pgen-0030009-b069] Kondo M, Shibata T, Kumagai T, Osawa T, Shibata N (2002). 15-Deoxy-Delta(12,14)-prostaglandin J(2): The endogenous electrophile that induces neuronal apoptosis. Proc Natl Acad Sci U S A.

[pgen-0030009-b070] Fuchs J, Huflejt ME, Rothfuss LM, Wilson DS, Carcamo G (1989). Impairment of enzymic and nonenzymic antioxidants in skin by UVB irradiation. J Invest Dermatol.

[pgen-0030009-b071] Shindo Y, Witt E, Han D, Packer L (1994). Dose-response effects of acute ultraviolet irradiation on antioxidants and molecular markers of oxidation in murine epidermis and dermis. J Invest Dermatol.

[pgen-0030009-b072] Podda M, Traber MG, Weber C, Yan LJ, Packer L (1998). UV-irradiation depletes antioxidants and causes oxidative damage in a model of human skin. Free Radic Biol Med.

[pgen-0030009-b073] Suzuki T, Spitz DR, Gandhi P, Lin HY, Crawford DR (2002). Mammalian resistance to oxidative stress: A comparative analysis. Gene Expr.

[pgen-0030009-b074] Harding HP, Zhang Y, Zeng H, Novoa I, Lu PD (2003). An integrated stress response regulates amino acid metabolism and resistance to oxidative stress. Mol Cell.

[pgen-0030009-b075] Murray JI, Whitfield ML, Trinklein ND, Myers RM, Brown PO (2004). Diverse and specific gene expression responses to stresses in cultured human cells. Mol Biol Cell.

[pgen-0030009-b076] Reimertz C, Kogel D, Rami A, Chittenden T, Prehn JH (2003). Gene expression during ER stress-induced apoptosis in neurons: Induction of the BH3-only protein Bbc3/PUMA and activation of the mitochondrial apoptosis pathway. J Cell Biol.

[pgen-0030009-b077] de Laat A, Kroon ED, de Gruijl FR (1997). Cell cycle effects and concomitant p53 expression in hairless murine skin after longwave UVA (365 nm) irradiation: A comparison with UVB irradiation. Photochem Photobiol.

[pgen-0030009-b078] Cable J, Jackson IJ, Steel KP (1995). Mutations at the W locus affect survival of neural crest-derived melanocytes in the mouse. Mech Dev.

[pgen-0030009-b079] Eisen MB, Spellman PT, Brown PO, Botstein D (1998). Cluster analysis and display of genome-wide expression patterns. Proc Natl Acad Sci U S A.

[pgen-0030009-b080] Manke IA, Nguyen A, Lim D, Stewart MQ, Elia AE (2005). MAPKAP kinase-2 is a cell cycle checkpoint kinase that regulates the G2/M transition and S phase progression in response to UV irradiation. Mol Cell.

[pgen-0030009-b081] Klezovitch O, Fernandez TE, Tapscott SJ, Vasioukhin V (2004). Loss of cell polarity causes severe brain dysplasia in Lgl1 knockout mice. Genes Dev.

[pgen-0030009-b082] Fritz G, Kaina B (2006). Rho GTPases: Promising cellular targets for novel anticancer drugs. Curr Cancer Drug Targets.

[pgen-0030009-b083] Shi L, Zhao G, Qiu D, Godfrey WR, Vogel H (2005). NF90 regulates cell cycle exit and terminal myogenic differentiation by direct binding to the 3'-untranslated region of MyoD and p21WAF1/CIP1 mRNAs. J Biol Chem.

[pgen-0030009-b084] Gooch JL, Toro JJ, Guler RL, Barnes JL (2004). Calcineurin A-alpha but not A-beta is required for normal kidney development and function. Am J Pathol.

[pgen-0030009-b085] Mishra S, Murphy LC, Nyomba BL, Murphy LJ (2005). Prohibitin: A potential target for new therapeutics. Trends Mol Med.

[pgen-0030009-b086] Verlinden L, Eelen G, Beullens I, Van Camp M, Van Hummelen P (2005). Characterization of the condensin component Cnap1 and protein kinase Melk as novel E2F target genes down-regulated by 1,25-dihydroxyvitamin D3. J Biol Chem.

[pgen-0030009-b087] Stern MH, Soulier J, Rosenzwajg M, Nakahara K, Canki-Klain N (1993). MTCP-1: a novel gene on the human chromosome Xq28 translocated to the T cell receptor alpha/delta locus in mature T cell proliferations. Oncogene.

[pgen-0030009-b088] Diehl JA, Yang W, Rimerman RA, Xiao H, Emili A (2003). Hsc70 regulates accumulation of cyclin D1 and cyclin D1-dependent protein kinase. Mol Cell Biol.

[pgen-0030009-b089] Angel P, Szabowski A, Schorpp-Kistner M (2001). Function and regulation of AP-1 subunits in skin physiology and pathology. Oncogene.

[pgen-0030009-b090] Bachmann M, Hennemann H, Xing PX, Hoffmann I, Moroy T (2004). The oncogenic serine/threonine kinase Pim-1 phosphorylates and inhibits the activity of Cdc25C-associated kinase 1 (C-TAK1): A novel role for Pim-1 at the G2/M cell cycle checkpoint. J Biol Chem.

[pgen-0030009-b091] Li T, Inoue A, Lahti JM, Kidd VJ (2004). Failure to proliferate and mitotic arrest of CDK11(p110/p58)-null mutant mice at the blastocyst stage of embryonic cell development. Mol Cell Biol.

[pgen-0030009-b092] Strezoska Z, Pestov DG, Lau LF (2002). Functional inactivation of the mouse nucleolar protein Bop1 inhibits multiple steps in pre-rRNA processing and blocks cell cycle progression. J Biol Chem.

[pgen-0030009-b093] Hong H, Kao C, Jeng MH, Eble JN, Koch MO (2004). Aberrant expression of CARM1, a transcriptional coactivator of androgen receptor, in the development of prostate carcinoma and androgen-independent status. Cancer.

[pgen-0030009-b094] Manjeshwar S, Branam DE, Lerner MR, Brackett DJ, Jupe ER (2003). Tumor suppression by the prohibitin gene 3' untranslated region RNA in human breast cancer. Cancer Res.

[pgen-0030009-b095] Ghosh I, Chowdhury AR, Rajeswari MR, Datta K (2004). Differential expression of hyaluronic acid binding protein 1 (HABP1)/P32/C1QBP during progression of epidermal carcinoma. Mol Cell Biochem.

[pgen-0030009-b096] Soulier J, Madani A, Cacheux V, Rosenzwajg M, Sigaux F (1994). The MTCP-1/c6.1B gene encodes for a cytoplasmic 8 kD protein overexpressed in T cell leukemia bearing a t(X;14) translocation. Oncogene.

[pgen-0030009-b097] Tarantul VZ, Nikolaev AI, Martynenko A, Hannig H, Hunsmann G (2000). Differential gene expression in B-cell non-Hodgkin's lymphoma of SIV-infected monkey. AIDS Res Hum Retroviruses.

[pgen-0030009-b098] Adams SM, Helps NR, Sharp MG, Brammar WJ, Walker RA (1992). Isolation and characterization of a novel gene with differential expression in benign and malignant human breast tumours. Hum Mol Genet.

[pgen-0030009-b099] Karan D, Kelly DL, Rizzino A, Lin MF, Batra SK (2002). Expression profile of differentially-regulated genes during progression of androgen-independent growth in human prostate cancer cells. Carcinogenesis.

[pgen-0030009-b100] Hu Y, Sun H, Drake J, Kittrell F, Abba MC (2004). From mice to humans: Identification of commonly deregulated genes in mammary cancer via comparative SAGE studies. Cancer Res.

[pgen-0030009-b101] Yen CC, Chen YJ, Pan CC, Lu KH, Chen PC (2005). Copy number changes of target genes in chromosome 3q25.3-qter of esophageal squamous cell carcinoma: TP63 is amplified in early carcinogenesis but down-regulated as disease progressed. World J Gastroenterol.

[pgen-0030009-b102] Lin H, Juang JL, Wang PS (2004). Involvement of Cdk5/p25 in digoxin-triggered prostate cancer cell apoptosis. J Biol Chem.

[pgen-0030009-b103] Hammer NA, Hansen TO, Byskov AG, Rajpert-De Meyts E, Grondahl ML (2005). Expression of IGF-II mRNA-binding proteins (IMPs) in gonads and testicular cancer. Reproduction.

[pgen-0030009-b104] Lakshmikuttyamma A, Selvakumar P, Kanthan R, Kanthan SC, Sharma RK (2005). Increased expression of calcineurin in human colorectal adenocarcinomas. J Cell Biochem.

[pgen-0030009-b105] Lin CW, Darzynkiewicz Z, Li X, Traganos F, Bedner E (2000). Differential expression of human topoisomerase IIIalpha during the cell cycle progression in HL-60 leukemia cells and human peripheral blood lymphocytes. Exp Cell Res.

[pgen-0030009-b106] Cleator S, Tsimelzon A, Ashworth A, Dowsett M, Dexter T (2006). Gene expression patterns for doxorubicin (Adriamycin) and cyclophosphamide (Cytoxan) (AC) response and resistance. Breast Cancer Res Treat.

[pgen-0030009-b107] Kubota T, Miyauchi M, Miura K, Hirokawa G, Awaya A (1998). Upregulation of nucleobindin expression in human-activated lymphocytes and non-Hodgkin's lymphoma. Pathol Int.

[pgen-0030009-b108] Singhal SS, Awasthi YC, Awasthi S (2006). Regression of melanoma in a murine model by RLIP76 depletion. Cancer Res.

[pgen-0030009-b109] Baugher PJ, Krishnamoorthy L, Price JE, Dharmawardhane SF (2005). Rac1 and Rac3 isoform activation is involved in the invasive and metastatic phenotype of human breast cancer cells. Breast Cancer Res.

[pgen-0030009-b110] Mikolajczyk SD, Millar LS, Marker KM, Rittenhouse HG, Wolfert RL (1999). Identification of a novel complex between human kallikrein 2 and protease inhibitor-6 in prostate cancer tissue. Cancer Res.

