# Supplementary material for: Distinct Pigmentary and Melanocortin 1 Receptor–Dependent Components of Cutaneous Defense against Ultraviolet Radiation
Source: PLoS Genet. 2007 Jan 12;3(1):e9. doi: 10.1371/journal.pgen.0030009 (PMC1774588; doi:10.1371/journal.pgen.0030009)
Supplement: Supplementary file 1 [file pgen.0030009.st001.pdf]

**Table S1.** UVB-Responsive Genes in the +/+ Basal Epidermis of P2.5 Mice				
**Accession ID**	**Gene Name**	**UVB Response**	**FDR-adjusted Student's *t* test *p*-Value**	**Average Fold Δ**
AV094752	Polymerase (RNA) II (DNA directed) polypeptide E	Repressed	1.14E-09	-1.67
AI837793	RIKEN cDNA 2410104I19 gene	Repressed	1.18E-09	-1.73
AV017919	Cadherin 13	Repressed	3.66E-09	-5.16
BG075546	Solute carrier family 14 (urea transporter), member 1	Repressed	5.31E-09	-2.06
AW554361	DNA segment, Chr X, Brigham & Women's Genetics 1396 expressed	Repressed	2.10E-07	-2.37
BG064583	Testis enhanced gene transcript	Repressed	2.32E-07	-2.33
BG073448	Sterol carrier protein 2, liver	Repressed	2.71E-07	-2.14
AV012453	RIKEN cDNA 2610507B11 gene	Repressed	2.72E-07	-2.46
BG075536	alpha 1	Repressed	5.93E-07	-2.01
AW548764	AW548764	Repressed	1.11E-06	-1.78
AV171635	alpha 1	Repressed	2.27E-06	-1.98
AV093721	Ribosomal protein S11	Repressed	7.91E-06	-1.99
AI838715	Ornithine aminotransferase	Repressed	8.30E-06	-2.25
AV016060	RIKEN cDNA 4930469P12 gene	Repressed	1.80E-05	-1.70
AW550140	AW550140	Repressed	2.19E-05	-1.70
BG073457	Expressed sequence AW549877	Repressed	2.44E-05	-3.56
AI838614	H19 fetal liver mRNA	Repressed	3.57E-05	-3.26
BG065902	Malignant fibrous histiocytoma amplified sequence 1	Repressed	3.69E-05	-2.78
AV022987	Nischarin	Repressed	3.69E-05	-1.45
AV085399	RIKEN cDNA 2410005O16 gene	Repressed	4.23E-05	-1.90
BG073025	Brain glycogen phosphorylase	Repressed	5.33E-05	-1.94
AV041015	Histone deacetylase 11	Repressed	6.79E-05	-2.55
BG065172	Solute carrier family 25 (mitochondrial carrier, adenine nucleotide translocator), member 5	Repressed	8.60E-05	-1.79
AV095135	Sperm associated antigen 7	Repressed	8.63E-05	-1.66
AV012849	Max interacting protein 1	Repressed	1.22E-04	-1.59
BG073247	Ribosomal protein S4, X-linked	Repressed	1.22E-04	-1.51
BG073933	TAF13 RNA polymerase II, TATA box binding protein (TBP)-associated factor	Repressed	1.41E-04	-1.57
BG069771	Ribosomal protein S3a	Repressed	1.53E-04	-1.82
AV095259	Origin recognition complex, subunit 5-like (S. cerevisiae)	Repressed	1.72E-04	-1.54
AU042511	Purine-nucleoside phosphorylase	Repressed	1.72E-04	-1.39
AV003958	Microsomal glutathione S-transferase 1	Repressed	1.83E-04	-2.29

AU020246	alpha 1	Repressed	2.25E-04	-2.09
AI605590	Histone 1, H1c	Repressed	2.39E-04	-2.48
AV074565	Expressed sequence AA536749	Repressed	2.46E-04	-1.41
BG076402	H3159D09	Repressed	2.80E-04	-1.46
BG073705	Ribosomal protein S3	Repressed	2.80E-04	-1.69
BG063840	H3011D05	Repressed	2.83E-04	-1.64
BG074268	Glutathione S-transferase, mu 1	Repressed	3.24E-04	-2.03
BG073252	Integral membrane protein 2B	Repressed	4.35E-04	-1.50
AV083198	Glucosidase, beta, acid	Repressed	4.36E-04	-1.66
AV107409	ATP synthase, H+ transporting mitochondrial F1 complex, beta subunit	Repressed	4.83E-04	-1.55
BG064817	Acidic ribosomal phosphoprotein P0	Repressed	4.85E-04	-1.56
AV067628	Fatty acid binding protein 2, intestinal	Repressed	5.88E-04	-1.51
AV084196	Adipose differentiation related protein	Repressed	6.32E-04	-1.64
AV059177	Cystatin C	Repressed	6.32E-04	-2.14
AV037303	Osteoclast stimulating factor 1	Repressed	6.32E-04	-2.33
AV171919	Ribosomal protein L22	Repressed	6.32E-04	-1.60
AV106281	AV106281	Repressed	6.56E-04	-1.67
AV061211	RNA binding motif protein 3	Repressed	6.70E-04	-1.56
BG065604	RIKEN cDNA 4933439C20 gene	Repressed	7.32E-04	-1.58
AV051534	Branched chain ketoacid dehydrogenase E1, beta polypeptide	Repressed	7.42E-04	-2.11
BG065259	Cytochrome b-5	Repressed	7.42E-04	-1.90
BG071068	Guanine nucleotide binding protein, beta 1	Repressed	7.42E-04	-1.53
AV053440	Monoglyceride lipase	Repressed	8.21E-04	-1.95
AI851021	Dehydrogenase/reductase (SDR family) member 3	Repressed	8.24E-04	-2.03
BG073449	Sterol carrier protein 2, liver	Repressed	8.63E-04	-1.70
2310050O13	2310050O13	Repressed	8.72E-04	-1.35
AV171864	Cystatin C	Repressed	8.94E-04	-1.79
BG072592	Ribosomal protein L3	Repressed	8.94E-04	-1.76
AV084156	(mitochondrial 3-oxoacyl-Coenzyme A thiolase)	Repressed	9.01E-04	-1.61
AV071317	2010323D05	Repressed	9.05E-04	-1.97
AV022721	Histidine ammonia lyase	Repressed	9.23E-04	-2.39
BG075997	Glutamyl-prolyl-tRNA synthetase	Repressed	9.54E-04	-1.49
BG064592	Diptheria toxin resistance protein required for diphthamide biosynthesis (Saccharomyces)-like 2	Repressed	9.68E-04	-1.41
BG068048	RIKEN cDNA 1110007A06 gene	Repressed	9.68E-04	-1.57
BG067161	RIKEN cDNA 2310067E08 gene	Repressed	9.69E-04	-2.09

BG069786	Tyrosine 3-monooxygenase/tryptophan 5-monooxygenase activation protein, beta polypeptide	Repressed	9.74E-04	-2.53
AV161761	ATP synthase, H+ transporting mitochondrial F1 complex, beta subunit	Repressed	1.00E-03	-1.71
BG064728	Heat shock protein 1 (chaperonin)	Repressed	1.01E-03	-1.74
BG071087	Sterol-C4-methyl oxidase-like	Repressed	1.05E-03	-1.88
BG066827	Enoyl Coenzyme A hydratase, short chain, 1, mitochondrial	Repressed	1.06E-03	-1.68
BG071337	Acyl-CoA synthetase long-chain family member 5	Repressed	1.17E-03	-1.55
AV059584	LIM domain containing preferred translocation partner in lipoma	Repressed	1.17E-03	-1.90
AV084246	Selenoprotein K	Repressed	1.17E-03	-1.72
BG073073	Acidic ribosomal phosphoprotein P0	Repressed	1.18E-03	-1.42
AW554113	AW554113	Repressed	1.23E-03	-2.21
BG064868	H3024G04	Repressed	1.23E-03	-1.89
AV033765	NADH dehydrogenase (ubiquinone) 1 alpha subcomplex 10	Repressed	1.23E-03	-1.36
BG075175	H19 fetal liver mRNA	Repressed	1.36E-03	-3.28
AV086729	Lipidosin	Repressed	1.36E-03	-1.69
AW556262	Piwi like homolog 2 (Drosophila)	Repressed	1.36E-03	-1.70
BG074034	Ribosomal protein L4	Repressed	1.36E-03	-2.50
BG068342	RIKEN cDNA 2610044O15 gene	Repressed	1.36E-03	-2.16
AV086400	RNA binding motif protein 3	Repressed	1.36E-03	-2.59
AV171750	Lactate dehydrogenase 2, B chain	Repressed	1.39E-03	-1.79
AA123885	Carnitine palmitoyltransferase 2	Repressed	1.42E-03	-1.85
AV094590	Eph receptor A5	Repressed	1.44E-03	-1.92
AV060544	RIKEN cDNA 5730589K01 gene	Repressed	1.46E-03	-1.43
BG069739	3-hydroxy-3-methylglutaryl-Coenzyme A synthase 1	Repressed	1.50E-03	-2.05
BG075245	Aldo-keto reductase family 1, member A4 (aldehyde reductase)	Repressed	1.51E-03	-1.41
BG065197	Ribosomal protein L5	Repressed	1.51E-03	-2.07
BG064983	Ubiquilin 1	Repressed	1.51E-03	-1.89
AV013520	Forkhead box O3a	Repressed	1.52E-03	-1.60
BG067445	Taxilin	Repressed	1.52E-03	-1.72
AV011553	Solute carrier family 30 (zinc transporter), member 4	Repressed	1.53E-03	-2.34
BG065525	Eukaryotic translation initiation factor 3, subunit 6	Repressed	1.55E-03	-1.62
BG072875	Proline-rich nuclear receptor coactivator 1	Repressed	1.55E-03	-1.57

BG065219	Cathepsin L	Repressed	1.57E-03	-1.72
AW556256	Ribosomal protein S6	Repressed	1.69E-03	-1.81
AA139015	Beta-2 microglobulin	Repressed	1.84E-03	-1.77
BG074847	Ubiquitin specific protease 10	Repressed	1.84E-03	-1.96
AV095097	LOC433110	Repressed	1.87E-03	-2.12
AV065300	Polyadenylate-binding protein-interacting protein 2	Repressed	1.99E-03	-1.66
AA221629	Carboxypeptidase Z	Repressed	2.02E-03	-1.87
AV088685	NADH dehydrogenase (ubiquinone) 1, alpha/beta subcomplex, 1	Repressed	2.02E-03	-1.92
BG075310	Transketolase	Repressed	2.02E-03	-1.56
BG076496	RIKEN cDNA 1200008A14 gene	Repressed	2.03E-03	-1.62
AV014557	Phenylalkylamine Ca2+ antagonist (emopamil) binding protein	Repressed	2.05E-03	-1.52
AV010020	Histidine ammonia lyase	Repressed	2.13E-03	-3.02
AV012335	gamma	Repressed	2.17E-03	-1.51
AV086173	Casein kinase 1, gamma 1	Repressed	2.20E-03	-1.45
BG074606	Ornithine aminotransferase	Repressed	2.20E-03	-1.98
AV036483	RAB9, member RAS oncogene family	Repressed	2.20E-03	-1.77
AI746663	Cathepsin D	Repressed	2.22E-03	-1.60
AV033315	ATP synthase, H+ transporting mitochondrial F1 complex, beta subunit	Repressed	2.31E-03	-1.40
BG063261	H19 fetal liver mRNA	Repressed	2.31E-03	-2.74
AV070671	Mitochondrial ribosomal protein L49	Repressed	2.31E-03	-2.03
AV104259	Integral membrane protein 2B	Repressed	2.35E-03	-1.83
BG073970	Pumilio 2 (Drosophila)	Repressed	2.36E-03	-1.53
BG064483	RAB18, member RAS oncogene family	Repressed	2.39E-03	-1.44
BG074898	Ribosomal protein L4	Repressed	2.39E-03	-1.91
BG074639	CDNA sequence BC043098	Repressed	2.45E-03	-1.61
BG073615	alpha 1	Repressed	2.45E-03	-2.35
C78484	alpha 1	Repressed	2.45E-03	-2.14
AV004594	Folate hydrolase	Repressed	2.45E-03	-1.75
BG073650	Voltage-dependent anion channel 1	Repressed	2.48E-03	-1.26
BG075588	Eukaryotic translation initiation factor 3, subunit 7 (zeta)	Repressed	2.53E-03	-1.41
BG065066	Eukaryotic translation initiation factor 4B	Repressed	2.53E-03	-1.40
AV017041	N-acetylneuraminate pyruvate lyase	Repressed	2.53E-03	-1.64
AV061426	SMT3 suppressor of mif two 3 homolog 1 (yeast)	Repressed	2.53E-03	-1.72
AI841363	Synaptotagmin 11	Repressed	2.53E-03	-1.42
BG074122	Sterol carrier protein 2, liver	Repressed	2.54E-03	-1.72
BG064879	RAB14, member RAS oncogene family	Repressed	2.62E-03	-1.65

BG073437	ATP synthase, H+ transporting mitochondrial F1 complex, beta subunit	Repressed	2.65E-03	-1.35
AV091639	Erythrocyte protein band 4.1	Repressed	2.65E-03	-1.38
AV081347	alpha 1	Repressed	2.65E-03	-2.04
BG065195	Ribosomal protein L5	Repressed	2.65E-03	-1.81
AW547555	AW547555	Repressed	2.69E-03	-1.46
AV083006	Cd200 antigen	Repressed	2.72E-03	-1.21
AK011603	2610028P08	Repressed	2.74E-03	-2.56
BG074683	Cleavage stimulation factor, 3' pre-RNA subunit 2	Repressed	2.74E-03	-1.76
BG072620	Ribosomal protein S6	Repressed	2.74E-03	-1.60
AV036152	1600017F23	Repressed	2.80E-03	-1.59
AV055902	RAB14, member RAS oncogene family	Repressed	2.85E-03	-1.80
AA142800	Spectrin alpha 2	Repressed	2.85E-03	-2.33
BG076103	Eukaryotic translation initiation factor 3, subunit 3 (gamma)	Repressed	2.97E-03	-1.90
AV031368	Integral membrane protein 2B	Repressed	3.01E-03	-1.75
BG068193	Nedd4 family interacting protein 1	Repressed	3.01E-03	-1.89
BG076357	Erythroid differentiation regulator 1	Repressed	3.09E-03	-2.28
AV069120	Solute carrier family 25 (mitochondrial carrier, adenine nucleotide translocator), member 5	Repressed	3.10E-03	-1.53
AI838561	RIKEN cDNA 4921506J03 gene	Repressed	3.14E-03	-2.89
2200004I23	2200004I23	Repressed	3.18E-03	-1.63
AW553234	FGFR1 oncogene partner 2	Repressed	3.18E-03	-2.17
AV010401	Integral membrane protein 2B	Repressed	3.18E-03	-1.14
AV001502	Aconitase 1	Repressed	3.19E-03	-2.48
AV078417	Dynein 2 light intermediate chain	Repressed	3.19E-03	-3.29
BG067835	Zinc finger, matrin type 2	Repressed	3.21E-03	-1.69
AI183128	Protein phosphatase 1, catalytic subunit, beta isoform	Repressed	3.22E-03	-1.46
AW556756	Phosphotriesterase related	Repressed	3.36E-03	-1.37
BG063856	Actin related protein 2/3 complex, subunit 1A	Repressed	3.39E-03	-1.08
AV054325	Ubiquilin 1	Repressed	3.39E-03	-1.46
BG072373	Serine protease inhibitor, Kunitz type 2	Repressed	3.45E-03	-1.62
BG072644	Solute carrier family 38, member 2	Repressed	3.47E-03	-1.53
BG064951	Splicing factor, arginine/serine-rich 3 (SRp20)	Repressed	3.54E-03	-1.42
AI840745	Trans-prenyltransferase	Repressed	3.54E-03	-1.64
BG073397	DNA segment, Chr 10, ERATO Doi 214, expressed	Repressed	3.55E-03	-1.66
AU023376	Tyrosinase	Repressed	3.55E-03	-1.93
AV103910	RAB11a, member RAS oncogene family	Repressed	3.57E-03	-1.46

BG070942	Polymerase (DNA-directed), delta interacting protein 3	Repressed	3.62E-03	-1.23
AV083988	RIKEN cDNA 2310004K06 gene	Repressed	3.72E-03	-1.38
BG075587	RIKEN cDNA 7530414M10 gene	Repressed	3.73E-03	-1.84
AV055199	Calsyntenin 1	Repressed	3.74E-03	-1.94
AI841334	H3 histone, family 3B	Repressed	3.74E-03	-1.45
AV171630	3300001I07	Repressed	3.75E-03	-1.62
AI841643	Platelet derived growth factor, B polypeptide	Repressed	3.75E-03	-1.75
AV007784	Retinoblastoma-like 2	Repressed	3.75E-03	-1.83
BG076117	Ribosomal protein L3	Repressed	3.75E-03	-1.72
AV078454	RIKEN cDNA 1300007B12 gene	Repressed	3.75E-03	-2.12
AV061704	1810074P07	Repressed	3.76E-03	-1.30
AW539206	Autophagy 7-like (S. cerevisiae)	Repressed	3.76E-03	-1.21
AV018960	Ras homolog gene family, member B	Repressed	3.76E-03	-1.61
AV108847	Guanine nucleotide binding protein-like 2 (nucleolar)	Repressed	3.81E-03	-1.83
AI851042	Activating transcription factor 5	Repressed	3.96E-03	-2.44
BG065405	Leukocyte receptor cluster (LRC) member 4	Repressed	3.97E-03	-1.53
AV106932	Adipose differentiation related protein	Repressed	3.99E-03	-1.86
BG074895	alpha 1	Repressed	4.00E-03	-2.30
AW551507	H3 histone, family 3B	Repressed	4.00E-03	-1.55
BG073604	Microsomal glutathione S-transferase 1	Repressed	4.00E-03	-1.92
AW558484	AW558484	Repressed	4.01E-03	-1.41
AV037280	Kelch-like 7 (Drosophila)	Repressed	4.01E-03	-1.64
AV073432	Mitochondrial ribosomal protein L30	Repressed	4.01E-03	-1.44
BG072823	Morf4 family associated protein 1	Repressed	4.01E-03	-1.44
BG066376	Progressive ankylosis	Repressed	4.01E-03	-2.36
BG074876	Electron transferring flavoprotein, alpha polypeptide	Repressed	4.09E-03	-1.75
AV086076	ATPase, Na+/K+ transporting, beta 3 polypeptide	Repressed	4.13E-03	-1.27
BG072595	Ribosomal protein L3	Repressed	4.13E-03	-1.73
BG075994	ADP-ribosylation factor-like 10C	Repressed	4.14E-03	-2.41
AW546231	AW546231	Repressed	4.15E-03	-1.78
AV162274	RIKEN cDNA 3110001I20 gene	Repressed	4.15E-03	-1.96
BG074596	Zona pellucida glycoprotein 2	Repressed	4.15E-03	-1.67
AW549995	AW549995	Repressed	4.25E-03	-1.83
BG074578	H1 histone family, member 0	Repressed	4.30E-03	-1.46
AV030536	OCIA domain containing 1	Repressed	4.40E-03	-1.33
BG065152	Ribosomal protein L15	Repressed	4.40E-03	-1.36
AV082197	Ferritin heavy chain 1	Repressed	4.43E-03	-1.63
AW545567	C0195C06	Repressed	4.48E-03	-4.93

BG065397	Ribosomal protein L30	Repressed	4.63E-03	-1.39
BG073564	Amyloid beta (A4) precursor protein	Repressed	4.76E-03	-1.51
AV095020	ATP synthase, H+ transporting mitochondrial F1 complex, beta subunit	Repressed	4.76E-03	-1.47
BG065194	Ribosomal protein L5	Repressed	4.76E-03	-1.98
AA140461	Solute carrier family 25 (mitochondrial carrier, adenine nucleotide translocator), member 5	Repressed	4.76E-03	-1.54
BG067903	Malate dehydrogenase 1, NAD (soluble)	Repressed	4.77E-03	-2.10
BG073317	H3121C03	Repressed	4.87E-03	-1.80
BG073071	Ribosomal protein S15a	Repressed	5.02E-03	-1.51
AV061197	RIKEN cDNA 1810073G14 gene	Repressed	5.09E-03	-1.53
BG069613	RIKEN cDNA 2700008N14 gene	Repressed	5.09E-03	-1.96
AV095255	ATP synthase, H+ transporting, mitochondrial F1 complex, alpha subunit, isoform 1	Repressed	5.12E-03	-1.72
BG065978	DNA segment, Chr 15, ERATO Doi 50, expressed	Repressed	5.12E-03	-3.03
BG073107	2	Repressed	5.12E-03	-1.51
BG063435	Protein phosphatase 2a, catalytic subunit, alpha isoform	Repressed	5.12E-03	-1.15
BG072891	BG072891	Repressed	5.26E-03	-1.36
BG075216	Matrin 3	Repressed	5.27E-03	-1.41
BG070116	Guanine nucleotide binding protein, beta 2, related sequence 1	Repressed	5.52E-03	-1.44
AV061276	Testis enhanced gene transcript	Repressed	5.52E-03	-1.33
AI837783	Calmodulin 3	Repressed	5.63E-03	-1.39
AA170700	Eukaryotic translation initiation factor 3, subunit 6	Repressed	5.67E-03	-1.72
BG073065	Ribosomal protein L5	Repressed	5.67E-03	-1.72
AV037348	ATPase, Na+/K+ transporting, beta 3 polypeptide	Repressed	5.70E-03	-1.78
AV070650	Ribosomal protein S3	Repressed	5.83E-03	-1.62
AV090161	ketoacyl-Coenzyme A thiolase/enoyl-Coenzyme A hydratase (trifunctional protein), beta subunit	Repressed	5.96E-03	-1.52
AV074746	Thymosin, beta 4, X chromosome	Repressed	5.98E-03	-1.84
AV014866	Proliferating cell nuclear antigen	Repressed	6.02E-03	-1.75
BG068503	(polypyrimidine tract binding protein associated)	Repressed	6.08E-03	-1.63
AV089761	Morf4 family associated protein 1	Repressed	6.10E-03	-1.94
AV055041	5	Repressed	6.14E-03	-2.16
BG072593	Ribosomal protein L3	Repressed	6.14E-03	-1.58
AV093840	Spermatogenesis associated, serine-rich 1	Repressed	6.14E-03	-1.65

AV035072	Sterol-C4-methyl oxidase-like	Repressed	6.17E-03	-1.48
AA034768	Mid1 interacting protein 1 (gastrulation specific G12-like (zebrafish))	Repressed	6.27E-03	-1.75
AV106382	Ribosomal protein S8	Repressed	6.44E-03	-2.49
AV030539	Histidyl-tRNA synthetase	Repressed	6.74E-03	-2.15
BG073708	Ubiquitin C	Repressed	6.74E-03	-1.75
BG064474	Ferritin light chain 1	Repressed	6.78E-03	-2.81
BG073954	H19 fetal liver mRNA	Repressed	6.80E-03	-2.45
AW554061	AW554061	Repressed	6.82E-03	-1.22
BG074450	Open reading frame 18	Repressed	6.82E-03	-1.46
BG067375	RIKEN cDNA A130038L21 gene	Repressed	6.82E-03	-1.62
AV032540	Gamma-aminobutyric acid (GABA-A) receptor-associated protein-like 2	Repressed	6.94E-03	-2.63
BG072061	Guanine nucleotide binding protein, alpha inhibiting 1	Repressed	6.94E-03	-1.41
AV083535	Protein phosphatase 1A, magnesium dependent, alpha isoform	Repressed	6.95E-03	-1.70
AW550503	AW550503	Repressed	7.21E-03	-1.40
AW553509	AW553509	Repressed	7.21E-03	-1.64
BG067798	Jun oncogene	Repressed	7.21E-03	-2.36
AV085274	Proteasome (prosome, macropain) 26S subunit, non-ATPase, 2	Repressed	7.21E-03	-1.18
AV059437	Ribonuclease, RNase A family, 1 (pancreatic)	Repressed	7.21E-03	-1.93
BG065641	Thioredoxin domain containing 5	Repressed	7.21E-03	-1.43
AA140080	Ubiquinol-cytochrome c reductase, Rieske iron-sulfur polypeptide 1	Repressed	7.35E-03	-1.41
BG072870	ATP synthase, H+ transporting, mitochondrial F1 complex, gamma polypeptide 1	Repressed	7.46E-03	-1.08
BG065300	Calsyntenin 1	Repressed	7.46E-03	-2.09
BG064570	Eukaryotic translation initiation factor 3, subunit 12	Repressed	7.47E-03	-1.27
AW558546	2310016C16Rik	Repressed	7.50E-03	-2.06
BG065175	Amine oxidase (flavin containing) domain 2	Repressed	7.56E-03	-1.38
AV004270	ATPase, H+ transporting, V0 subunit C	Repressed	7.56E-03	-1.83
BG069644	Lipidosin	Repressed	7.59E-03	-1.60
AV093842	Apolipoprotein E	Repressed	7.69E-03	-2.00
BG064450	RIKEN cDNA 1200015F23 gene	Repressed	7.73E-03	-1.25
AI851027	Testis enhanced gene transcript	Repressed	7.73E-03	-1.64
AV083963	DEAH (Asp-Glu-Ala-His) box polypeptide 15	Repressed	7.85E-03	-1.23
AV019903	Protein tyrosine phosphatase, non-receptor type 11	Repressed	7.85E-03	-1.41
AV060444	Ribosomal protein L4	Repressed	7.85E-03	-1.84

AV094558	Nuclear transcription factor-Y beta	Repressed	7.87E-03	-1.31
AV037272	DNA segment, Chr 11, KL Mohlke 35	Repressed	7.88E-03	-1.87
BG071088	Lemur tyrosine kinase 2	Repressed	7.96E-03	-2.01
AV103135	AV103135	Repressed	7.97E-03	-1.25
AV033691	Integral membrane protein 2B	Repressed	7.97E-03	-1.51
AV141401	ADP-ribosylation factor-like 5	Repressed	8.04E-03	-1.53
BG063843	Cytochrome c oxidase subunit VIIa polypeptide 2-like	Repressed	8.09E-03	-1.46
BG074479	Actin related protein 2/3 complex, subunit 1A	Repressed	8.15E-03	-1.23
AV033259	Heterogeneous nuclear ribonucleoproteins methyltransferase-like 2 (S. cerevisiae)	Repressed	8.27E-03	-2.10
BG071324	IK cytokine	Repressed	8.27E-03	-1.52
AI840760	RNA binding motif protein 9	Repressed	8.27E-03	-1.95
AV057406	Microsomal glutathione S-transferase 1	Repressed	8.33E-03	-1.69
BG064089	ELOVL family member 6, elongation of long chain fatty acids (yeast)	Repressed	8.36E-03	-1.59
AV095045	Ribosomal protein S3	Repressed	8.38E-03	-1.79
BY356335	Laminin receptor 1 (ribosomal protein SA)	Repressed	8.42E-03	-1.89
AV053944	OCIA domain containing 1	Repressed	8.42E-03	-2.03
AV108296	Open reading frame 18	Repressed	8.42E-03	-1.48
BG073236	Ribosomal protein 10	Repressed	8.42E-03	-1.44
BG068210	Protein tyrosine phosphatase, non-receptor type 11	Repressed	8.50E-03	-1.15
AV084807	Selenoprotein K	Repressed	8.51E-03	-1.71
BG063299	ATP synthase, H+ transporting mitochondrial F1 complex, beta subunit	Repressed	8.59E-03	-1.45
AW548883	AW548883	Repressed	8.59E-03	-1.29
AV057014	RIKEN cDNA 1810044A24 gene	Repressed	8.59E-03	-2.07
BG075919	8	Repressed	8.61E-03	-1.48
BG075350	Guanosine diphosphate (GDP) dissociation inhibitor 3	Repressed	8.69E-03	-1.52
AV090727	Ubiquitin-conjugating enzyme E2D 3 (UBC4/5 homolog, yeast)	Repressed	8.74E-03	-1.21
AA408467	Poly(A)-specific ribonuclease (deadenylation nuclease)	Repressed	8.75E-03	-1.35
AV095167	Glutathione peroxidase 1	Repressed	8.79E-03	-1.11
BG073370	Tetratricopeptide repeat domain 3	Repressed	8.79E-03	-1.54
AW554960	AW554960	Repressed	8.94E-03	-3.13
BG074733	BG074733	Repressed	9.07E-03	-1.16
AW545291	Calmodulin 1	Repressed	9.07E-03	-1.84
BG065740	Heterogeneous nuclear ribonucleoprotein A1	Repressed	9.07E-03	-1.45

AV003213	UDP glucuronosyltransferase 1 family, polypeptide A6A	Repressed	9.07E-03	-1.38
BG070002	CAMP responsive element binding protein 3	Repressed	9.20E-03	-2.01
AV171591	Nucleophosmin 1	Repressed	9.20E-03	-1.59
BG075714	3-hydroxyisobutyrate dehydrogenase	Repressed	9.25E-03	-2.13
AV114407	Rho-related BTB domain containing 3	Repressed	9.25E-03	-2.43
BG073248	Ribosomal protein S4, X-linked	Repressed	9.26E-03	-1.43
BG074060	RIKEN cDNA 9530068E07 gene	Repressed	9.29E-03	-1.46
AA036237	3-hydroxyisobutyrate dehydrogenase	Repressed	9.42E-03	-1.85
AV088169	Calmodulin 4	Repressed	9.42E-03	-1.68
AA119251	Mitogen activated protein kinase 1	Repressed	9.42E-03	-1.40
BG063861	Ribosomal protein L3	Repressed	9.47E-03	-1.45
AV084484	Small EDRK-rich factor 2	Repressed	9.54E-03	-1.43
BG073064	Spectrin alpha 2	Repressed	9.54E-03	-1.39
AV065884	2010104L04	Repressed	9.55E-03	-2.29
AV070290	Diazepam binding inhibitor	Repressed	9.55E-03	-1.80
BG065615	CDNA sequence BC053393	Repressed	9.63E-03	-1.30
AV058848	gamma	Repressed	9.63E-03	-1.48
BG073515	YTH domain family 3	Repressed	9.63E-03	-1.44
AW554034	AW554034	Repressed	9.98E-03	-1.19
AA656056	Nonagouti	Repressed	1.01E-02	-1.77
AV030897	Cystatin C	Repressed	1.02E-02	-1.48
AW556925	GNAS (guanine nucleotide binding protein, alpha stimulating) complex locus	Repressed	1.02E-02	-2.06
BG073275	Ring finger protein 10	Repressed	1.02E-02	-1.18
AW538269	C0105E04	Repressed	1.03E-02	-1.50
AV037400	Cbp/p300-interacting transactivator, with Glu/Asp-rich carboxy-terminal domain, 2	Repressed	1.03E-02	-1.37
BG075647	Cyclin I	Repressed	1.03E-02	-1.48
AV083871	F11 receptor	Repressed	1.03E-02	-1.58
AV000213	Fumarylacetoacetate hydrolase domain containing 2A	Repressed	1.03E-02	-1.33
BG074635	NADH dehydrogenase (ubiquinone) Fe-S protein 2	Repressed	1.03E-02	-1.59
BG064666	Ubiquitin-activating enzyme E1, Chr X	Repressed	1.03E-02	-1.52
BE629378	Protein phosphatase 1, catalytic subunit, beta isoform	Repressed	1.05E-02	-1.64
AV141396	Ras suppressor protein 1	Repressed	1.05E-02	-1.33
AV016274	Actin-binding LIM protein 1	Repressed	1.06E-02	-1.57
BG069344	Acyl-CoA synthetase long-chain family member 4	Repressed	1.06E-02	-1.69
BG069075	expressed sequence AU024582	Repressed	1.06E-02	-1.44

BG071577	N-acetylneuraminate pyruvate lyase	Repressed	1.06E-02	-1.82
BG073805	Nischarin	Repressed	1.06E-02	-1.50
AV011766	DNA segment, Chr 6, Wayne State University 176, expressed	Repressed	1.07E-02	-2.73
BG064735	Peroxiredoxin 6	Repressed	1.08E-02	-1.70
AV017252	F-box and leucine-rich repeat protein 17	Repressed	1.09E-02	-2.86
BG063923	H3 histone, family 3B	Repressed	1.09E-02	-1.24
BG075646	Praja 2, RING-H2 motif containing	Repressed	1.09E-02	-1.73
AW552118	Terf1 (TRF1)-interacting nuclear factor 2	Repressed	1.09E-02	-1.75
BI076857	BI076857	Repressed	1.10E-02	-1.42
AV088394	Calmodulin 4	Repressed	1.10E-02	-1.53
AV038433	Echinoderm microtubule associated protein like 2	Repressed	1.10E-02	-2.18
BG072496	ELOVL family member 5, elongation of long chain fatty acids (yeast)	Repressed	1.10E-02	-2.12
AV089939	Eukaryotic translation initiation factor 4B	Repressed	1.10E-02	-1.70
AV085724	Histone 1, H2bg	Repressed	1.10E-02	-1.57
AV095051	Splicing factor, arginine/serine-rich 5 (SRp40, HRS)	Repressed	1.11E-02	-1.57
AV048511	Gamma-glutamyl hydrolase	Repressed	1.12E-02	-1.95
AI841014	GNAS (guanine nucleotide binding protein, alpha stimulating) complex locus	Repressed	1.12E-02	-2.23
BG076334	RIKEN cDNA 1810044A24 gene	Repressed	1.12E-02	-1.74
BG073503	Small EDRK-rich factor 2	Repressed	1.12E-02	-1.29
BG073653	Calcium binding protein 39	Repressed	1.13E-02	-1.33
BG074598	Pyruvate carboxylase	Repressed	1.13E-02	-1.94
AV103514	ATPase, H+ transporting, V0 subunit D isoform 1	Repressed	1.14E-02	-1.21
BG063994	Beta-2 microglobulin	Repressed	1.14E-02	-1.66
AV088774	Electron transferring flavoprotein, alpha polypeptide	Repressed	1.14E-02	-1.56
C78483	alpha 1	Repressed	1.14E-02	-1.65
AV053864	Hepatitis B virus x interacting protein	Repressed	1.14E-02	-1.32
AV088731	Tyrosine 3-monooxygenase/tryptophan 5-monooxygenase activation protein, epsilon polypeptide	Repressed	1.14E-02	-1.51
BG075176	H19 fetal liver mRNA	Repressed	1.15E-02	-3.24
AV032980	Lactate dehydrogenase 2, B chain	Repressed	1.15E-02	-1.79
BG063216	RNA, U64 small nucleolar	Repressed	1.15E-02	-1.42
BG074328	alpha 1	Repressed	1.17E-02	-1.58
AI841338	RAB6, member RAS oncogene family	Repressed	1.17E-02	-1.83
BG064729	Heat shock protein 1 (chaperonin)	Repressed	1.18E-02	-1.45

AV014153	Proteasome (prosome, macropain) 26S subunit, non-ATPase, 8	Repressed	1.18E-02	-1.20
BG064542	Proteasome (prosome, macropain) subunit, beta type 1	Repressed	1.18E-02	-1.49
BG075073	Thymosin, beta 4, X chromosome	Repressed	1.18E-02	-2.37
AV141532	Carnitine deficiency-associated gene expressed in ventricle 3	Repressed	1.19E-02	-1.52
BG074978	Four and a half LIM domains 1	Repressed	1.19E-02	-1.82
BG073156	Kelch-like 17 (Drosophila)	Repressed	1.19E-02	-1.72
AV052188	Multiple coagulation factor deficiency 2	Repressed	1.19E-02	-1.99
AV015004	1	Repressed	1.20E-02	-1.42
BG067305	Phosphatidic acid phosphatase type 2B	Repressed	1.22E-02	-2.33
BG065344	A kinase (PRKA) anchor protein 8	Repressed	1.26E-02	-1.52
BG065538	3-hydroxy-3-methylglutaryl-Coenzyme A reductase	Repressed	1.27E-02	-1.34
BG073524	Serine protease inhibitor, Kunitz type 2	Repressed	1.27E-02	-1.53
BG065196	Ribosomal protein L5	Repressed	1.28E-02	-2.02
BG072328	RIKEN cDNA 1810046J19 gene	Repressed	1.28E-02	-1.34
AW550308	Tripartite motif-containing 44	Repressed	1.28E-02	-1.37
BG065440	Upstream transcription factor 2	Repressed	1.28E-02	-1.63
AV052537	2	Repressed	1.29E-02	-1.58
BG069614	Nudix (nucleoside diphosphate linked moiety X)-type motif 7	Repressed	1.29E-02	-2.29
AV020794	RIKEN cDNA 1110007A06 gene	Repressed	1.29E-02	-1.72
BG063157	RIKEN cDNA 2410104I19 gene	Repressed	1.29E-02	-1.07
AV085270	Schwannomin interacting protein 1	Repressed	1.29E-02	-2.17
AV054658	2	Repressed	1.29E-02	-1.14
AV061508	Biphenyl hydrolase-like (serine hydrolase, breast epithelial mucin-associated antigen)	Repressed	1.30E-02	-2.44
AV084290	Glutamate dehydrogenase 1	Repressed	1.30E-02	-1.16
AV081997	Histocompatibility 2, D region locus 1	Repressed	1.30E-02	-2.16
AV008954	Poly(rC) binding protein 2	Repressed	1.30E-02	-1.69
BG065216	RIKEN cDNA 2700060E02 gene	Repressed	1.30E-02	-1.26
AV094502	Tyrosine 3-monooxygenase/tryptophan 5-monooxygenase activation protein, theta polypeptide	Repressed	1.30E-02	-1.45
BG075353	Zinc finger, FYVE domain containing 21	Repressed	1.30E-02	-1.40
AV106965	Ribosomal protein L32	Repressed	1.31E-02	-1.70
BG073773	alpha 1	Repressed	1.32E-02	-1.55
BG065187	SEC13-like 1 (S. cerevisiae)	Repressed	1.32E-02	-1.20
BG066529	Ribonuclease T2	Repressed	1.33E-02	-1.44
BB667069	2310041E13	Repressed	1.34E-02	-1.48

AV043826	Acyl-CoA synthetase long-chain family member 5	Repressed	1.34E-02	-1.43
AV033633	Adducin 3 (gamma)	Repressed	1.34E-02	-1.54
AW545296	ATPase, H+ transporting, V1 subunit A, isoform 1	Repressed	1.34E-02	-1.76
AW549514	AW549514	Repressed	1.34E-02	-1.56
BG067866	Coxsackievirus and adenovirus receptor	Repressed	1.34E-02	-1.30
AV061681	gamma	Repressed	1.34E-02	-1.62
AV052630	General transcription factor IIB	Repressed	1.34E-02	-1.55
AV135840	Inhibitor of DNA binding 2	Repressed	1.34E-02	-1.78
AV056273	Kelch-like 7 (Drosophila)	Repressed	1.34E-02	-1.68
AV085439	RIKEN cDNA 2310002J15 gene	Repressed	1.34E-02	-1.98
AV094597	Solute carrier family 25 (mitochondrial carrier, phosphate carrier), member 3	Repressed	1.34E-02	-1.64
AV103717	Stromal cell derived factor 2	Repressed	1.34E-02	-1.22
AW557711	alpha 1	Repressed	1.35E-02	-1.69
AV039876	1700009P10	Repressed	1.36E-02	-1.64
AV013104	ADP-ribosylation factor-like 6 interacting protein 1	Repressed	1.36E-02	-1.32
BG074892	Annexin A6	Repressed	1.36E-02	-1.64
AA410046	RIKEN cDNA 4921517N04 gene	Repressed	1.36E-02	-1.79
BG072874	Secreted acidic cysteine rich glycoprotein	Repressed	1.36E-02	-1.56
AV019354	Down syndrome critical region homolog 1 (human)	Repressed	1.38E-02	-1.53
AI838638	Anaplastic lymphoma kinase	Repressed	1.39E-02	-2.35
BG066638	POU domain, class 6, transcription factor 1	Repressed	1.40E-02	-1.85
AV060618	B-cell translocation gene 2, anti-proliferative	Repressed	1.42E-02	-1.40
2310020E16	2310020E16	Repressed	1.45E-02	-2.32
BG073001	Cellular nucleic acid binding protein 1	Repressed	1.45E-02	-1.59
BG074908	H19 fetal liver mRNA	Repressed	1.45E-02	-1.61
AV015754	Pre B-cell leukemia transcription factor 1	Repressed	1.45E-02	-1.52
AV094794	Ribosomal protein L37	Repressed	1.45E-02	-1.38
AV082005	RIKEN cDNA 0610040D20 gene	Repressed	1.45E-02	-1.44
BG070131	Spindlin	Repressed	1.47E-02	-1.07
BG065603	Cytochrome c oxidase subunit IV isoform 1	Repressed	1.49E-02	-1.35
BG065107	Guanine nucleotide binding protein, beta 2, related sequence 1	Repressed	1.49E-02	-1.21
AI841373	Latexin	Repressed	1.49E-02	-1.73
AV082302	Peroxiredoxin 6	Repressed	1.49E-02	-1.27
AK117104	2610028K08	Repressed	1.52E-02	-1.44
AV052379	Death-associated protein	Repressed	1.52E-02	-1.21
AI840046	RIKEN cDNA 2310047D13 gene	Repressed	1.52E-02	-1.73

AV036285	CDNA sequence BC031181	Repressed	1.53E-02	-1.59
BG064782	DEAD (Asp-Glu-Ala-Asp) box polypeptide 5	Repressed	1.53E-02	-1.40
AV107070	Ribosomal protein S8	Repressed	1.53E-02	-2.13
BI076687	WASP family 1	Repressed	1.53E-02	-1.34
BG074458	24-dehydrocholesterol reductase	Repressed	1.55E-02	-1.68
AW546688	AW546688	Repressed	1.55E-02	-2.44
AA162760	Keratin complex 1, acidic, gene 10	Repressed	1.55E-02	-2.80
BG066575	Jumonji, AT rich interactive domain 2	Repressed	1.56E-02	-1.46
BG066357	Mitogen activated protein kinase kinase kinase 12	Repressed	1.56E-02	-1.76
AW555786	AW555786	Repressed	1.57E-02	-1.30
BG069673	Mortality factor 4 like 2	Repressed	1.57E-02	-1.65
W53283	Notch gene homolog 3 (Drosophila)	Repressed	1.58E-02	-1.55
2210003C24	2210003C24	Repressed	1.59E-02	-1.80
AV085190	2310010I19	Repressed	1.59E-02	-1.72
BG075244	Aldo-keto reductase family 1, member A4 (aldehyde reductase)	Repressed	1.59E-02	-1.42
AV078540	ADP-ribosylation factor guanine nucleotide-exchange factor 1(brefeldin A-inhibited)	Repressed	1.62E-02	-1.86
AV057751	Glutathione S-transferase, mu 1	Repressed	1.62E-02	-1.62
BG072621	Nucleophosmin 1	Repressed	1.62E-02	-1.20
BG065091	RIKEN cDNA 2400003C14 gene	Repressed	1.62E-02	-1.17
AV054009	Scleraxis	Repressed	1.62E-02	-1.81
AW988741	Secreted acidic cysteine rich glycoprotein	Repressed	1.62E-02	-1.33
AV065416	Nucleosome assembly protein 1-like 1	Repressed	1.63E-02	-1.93
AV032929	Hippocalcin-like 1	Repressed	1.66E-02	-1.80
AV053031	Stearoyl-Coenzyme A desaturase 1	Repressed	1.66E-02	-1.82
BG069990	Bcl2-associated athanogene 1	Repressed	1.67E-02	-1.53
AV113950	Karyopherin (importin) alpha 3	Repressed	1.68E-02	-1.74
AV032500	Prion protein	Repressed	1.68E-02	-1.55
BG076310	RIKEN cDNA 1810014L12 gene	Repressed	1.68E-02	-1.31
AV018850	RIKEN cDNA 1810058I24 gene	Repressed	1.68E-02	-1.93
AV084769	RIKEN cDNA 2310003P10 gene	Repressed	1.68E-02	-1.59
AI840618	RIKEN cDNA 4921506J03 gene	Repressed	1.68E-02	-1.20
AV071376	Transmembrane protein 14C	Repressed	1.70E-02	-1.68
AV061394	Dehydrogenase/reductase (SDR family) member 4	Repressed	1.72E-02	-1.91
AV084923	RIKEN cDNA 2310076L09 gene	Repressed	1.72E-02	-1.51
BG067773	3-oxoacid CoA transferase 1	Repressed	1.73E-02	-2.71
BG072552	(mitochondrial 3-oxoacyl-Coenzyme A thiolase)	Repressed	1.73E-02	-1.76
BG067893	Alanyl-tRNA synthetase	Repressed	1.73E-02	-1.48

AV103569	Catalase	Repressed	1.73E-02	-1.54
BE305962	Catenin beta	Repressed	1.73E-02	-1.72
AV094441	DNA methyltransferase 2	Repressed	1.73E-02	-1.79
BG073681	gamma	Repressed	1.73E-02	-1.21
AV014878	Monoglyceride lipase	Repressed	1.73E-02	-1.70
BG063719	Ribosomal protein L4	Repressed	1.73E-02	-1.69
AV032714	Sterol carrier protein 2, liver	Repressed	1.73E-02	-1.64
AV089898	Electron transferring flavoprotein, alpha polypeptide	Repressed	1.74E-02	-1.38
BG065263	Proteasome (prosome, macropain) subunit, alpha type 3	Repressed	1.74E-02	-1.53
AV072259	Ribosomal protein L30	Repressed	1.74E-02	-1.64
AV089006	Dual specificity phosphatase 14	Repressed	1.75E-02	-1.78
BG066296	Casein kinase II, beta subunit	Repressed	1.76E-02	-1.37
AW554618	Diaphanous homolog 1 (Drosophila)	Repressed	1.77E-02	-1.27
AI854079	DNA segment, Chr 9, Wayne State University 20, expressed	Repressed	1.77E-02	-2.55
BG064778	T-complex protein 1	Repressed	1.77E-02	-1.24
BG065069	Eukaryotic translation initiation factor 4, gamma 2	Repressed	1.78E-02	-1.16
BG074107	Ribosomal protein L3	Repressed	1.78E-02	-1.61
C78001	subunit	Repressed	1.79E-02	-1.94
AV140966	2810407D09	Repressed	1.80E-02	-1.68
AV020195	Cyclin I	Repressed	1.80E-02	-1.34
BE307724	Prosaposin	Repressed	1.80E-02	-3.25
BG072985	Ribosomal protein L7	Repressed	1.80E-02	-1.56
BG072201	RIKEN cDNA 1200015A19 gene	Repressed	1.80E-02	-1.33
1810038O03	1810038O03	Repressed	1.81E-02	-1.81
BG072463	H1 histone family, member 0	Repressed	1.81E-02	-1.44
AV095215	Leukotriene A4 hydrolase	Repressed	1.81E-02	-1.16
AV094839	Eukaryotic translation elongation factor 1 delta (guanine nucleotide exchange protein)	Repressed	1.82E-02	-1.54
AV074598	Protein kinase C and casein kinase substrate in neurons 3	Repressed	1.83E-02	-1.38
AV088659	Serine (or cysteine) proteinase inhibitor, clade B (ovalbumin), member 12	Repressed	1.83E-02	-1.43
BG073325	RIKEN cDNA F830004M19 gene	Repressed	1.84E-02	-1.67
BG072415	CGG triplet repeat binding protein 1	Repressed	1.85E-02	-1.38
BG073964	Protein-L-isoaspartate (D-aspartate) O-methyltransferase 1	Repressed	1.85E-02	-1.54
BG070989	Reticulon 4	Repressed	1.85E-02	-1.66
BG064783	DEAD (Asp-Glu-Ala-Asp) box polypeptide 5	Repressed	1.88E-02	-1.04

AV085319	Expressed sequence AW552001	Repressed	1.88E-02	-1.56
BG073162	Oxysterol binding protein-like 1A	Repressed	1.88E-02	-1.71
BG070422	Pumilio 2 (Drosophila)	Repressed	1.88E-02	-1.66
AV033648	RIKEN cDNA 1500010J02 gene	Repressed	1.88E-02	-1.64
AV025443	Brain protein 17	Repressed	1.89E-02	-1.53
BG071421	Inhibitor of DNA binding 2	Repressed	1.89E-02	-2.64
AW557873	Inhibitor of DNA binding 3	Repressed	1.89E-02	-1.72
AV112997	Mortality factor 4 like 1	Repressed	1.89E-02	-1.43
AV085507	protein 2	Repressed	1.89E-02	-2.06
AV088072	RIKEN cDNA 2310034L04 gene	Repressed	1.89E-02	-2.13
AV104148	Secreted acidic cysteine rich glycoprotein	Repressed	1.89E-02	-1.86
AU017673	TatD DNase domain containing 2	Repressed	1.89E-02	-1.71
AW552886	Valosin containing protein	Repressed	1.89E-02	-1.25
BG071303	X-box binding protein 1	Repressed	1.90E-02	-1.47
AW548385	AW548385	Repressed	1.91E-02	-1.89
BG067357	Calponin 1	Repressed	1.92E-02	-1.24
AV059616	Gamma-glutamyl hydrolase	Repressed	1.92E-02	-1.79
AU041598	Histocompatibility 2, K1, K region	Repressed	1.92E-02	-1.38
AI840617	Sequestosome 1	Repressed	1.92E-02	-1.41
BG072695	(Drosophila)	Repressed	1.93E-02	-1.24
AA144790	T-complex protein 1	Repressed	1.93E-02	-1.34
AV089488	expressed sequence AI642036	Repressed	1.94E-02	-1.23
AV134975	RIKEN cDNA 2810012H18 gene	Repressed	1.94E-02	-1.31
AW538486	C0108B10	Repressed	1.95E-02	-1.28
AW556830	Dynactin 2	Repressed	1.95E-02	-1.24
AV059483	Poly(rC) binding protein 2	Repressed	1.95E-02	-1.29
AV053681	Ribosomal protein L4	Repressed	1.96E-02	-1.89
BG064234	Progesterone receptor membrane component 1	Repressed	1.98E-02	-1.41
AW548804	SWI/SNF related, matrix associated, actin dependent regulator of chromatin, subfamily b, member 1	Repressed	1.98E-02	-1.17
BG074338	Voltage-dependent anion channel 3	Repressed	1.98E-02	-1.14
BG064678	GLE1 RNA export mediator-like (yeast	Repressed	1.99E-02	-2.30
AW988741	Secreted acidic cysteine rich glycoprotein	Repressed	1.99E-02	-1.32
BG065535	Fibronectin type 3 and SPRY domain-containing protein	Repressed	2.01E-02	-4.25
BG075217	RIKEN cDNA 1200006O19 gene	Repressed	2.01E-02	-2.70
AV140878	RIKEN cDNA 2310008M10 gene	Repressed	2.02E-02	-1.64
BG064609	RIKEN cDNA 2810409H07 gene	Repressed	2.02E-02	-1.52
AV088458	Thimet oligopeptidase 1	Repressed	2.02E-02	-2.43
BG064661	Brain abundant, membrane attached signal protein 1	Repressed	2.04E-02	-4.47

AV070951	Pentaxin related gene	Repressed	2.04E-02	-1.72
BG071748	Ubiquitin-conjugating enzyme E2S	Repressed	2.04E-02	-1.42
BG071112	Vesicle-associated membrane protein, associated protein A	Repressed	2.04E-02	-1.52
AW546823	AW546823	Repressed	2.08E-02	-1.52
AV069270	7	Repressed	2.08E-02	-1.49
AV043801	Nudix (nucleoside diphosphate linked moiety X)-type motif 4	Repressed	2.08E-02	-1.53
BG075727	Poly(rC) binding protein 2	Repressed	2.08E-02	-1.25
BG067321	Regulator of G-protein signaling 2	Repressed	2.08E-02	-1.70
AV054075	RIKEN cDNA 0610040D20 gene	Repressed	2.08E-02	-1.40
AV103649	RIKEN cDNA 1110030J09 gene	Repressed	2.08E-02	-1.68
BG074894	Thioredoxin domain containing 7	Repressed	2.08E-02	-1.51
AV095037	Acidic ribosomal phosphoprotein P0	Repressed	2.10E-02	-1.26
AV031502	ATPase, H+ transporting, V1 subunit A, isoform 1	Repressed	2.10E-02	-1.90
BG074307	ATP-binding cassette, sub-family B (MDR/TAP), member 7	Repressed	2.10E-02	-1.36
AV033538	Lactate dehydrogenase 2, B chain	Repressed	2.10E-02	-1.52
AV024103	N-acetylneuraminate pyruvate lyase	Repressed	2.10E-02	-1.89
AV095052	Proteasome (prosome, macropain) subunit, alpha type 4	Repressed	2.10E-02	-1.95
BG065356	Ribosomal protein L30	Repressed	2.10E-02	-1.60
AV094943	Ribosomal protein L4	Repressed	2.10E-02	-1.80
AV095042	Ribosomal protein L5	Repressed	2.10E-02	-1.91
AV103708	Ribosomal protein L9	Repressed	2.10E-02	-1.67
AV094539	RIKEN cDNA 1110004B13 gene	Repressed	2.10E-02	-1.52
BG072804	Speckle-type POZ protein	Repressed	2.10E-02	-1.59
AV040481	WW domain binding protein 2	Repressed	2.10E-02	-1.39
AV171545	Dynein, cytoplasmic, intermediate chain 2	Repressed	2.11E-02	-1.16
BG071411	Acyl-Coenzyme A dehydrogenase, short/branched chain	Repressed	2.12E-02	-1.27
AV007140	Chondroitin sulfate proteoglycan 4	Repressed	2.12E-02	-2.51
AV065480	7	Repressed	2.12E-02	-1.56
BG072077	Insulin-like growth factor binding protein 4	Repressed	2.13E-02	-1.77
BG075463	Heat shock protein 1, beta	Repressed	2.15E-02	-1.82
AV013144	L-3-hydroxyacyl-Coenzyme A dehydrogenase, short chain	Repressed	2.15E-02	-1.38
AV026104	NudC domain containing 3	Repressed	2.15E-02	-1.77
BG062962	RAB1, member RAS oncogene family	Repressed	2.15E-02	-1.28
BG074372	Storage granule protein 23	Repressed	2.15E-02	-1.13
AV000446	0610008C16	Repressed	2.19E-02	-1.35

AV032265	Apolipoprotein E	Repressed	2.19E-02	-1.64
AV106468	RNA terminal phosphate cyclase-like 1	Repressed	2.19E-02	-1.25
BG065151	Ribosomal protein L15	Repressed	2.20E-02	-1.39
BG072594	Ribosomal protein L3	Repressed	2.20E-02	-1.46
AA137292	Chemokine (C-C motif) ligand 19	Repressed	2.23E-02	-1.65
AV067598	Cyclin-dependent kinase inhibitor 2B (p15, inhibits CDK4)	Repressed	2.23E-02	-1.68
BG073821	Dendritic cell protein GA17	Repressed	2.23E-02	-1.59
BG074397	Glutathione S-transferase, mu 1	Repressed	2.23E-02	-1.17
AV074036	Golgi SNAP receptor complex member 2	Repressed	2.24E-02	-1.36
AA760344	Keratin complex 1, acidic, gene 15	Repressed	2.24E-02	-1.74
AV032030	Praja1, RING-H2 motif containing	Repressed	2.24E-02	-1.85
AA137489	Casein kinase 1, alpha 1	Repressed	2.25E-02	-1.52
AV095469	Ribosomal protein L19	Repressed	2.25E-02	-1.49
AV104173	RIKEN cDNA 2510003E04 gene	Repressed	2.25E-02	-1.59
BG065013	Secreted acidic cysteine rich glycoprotein	Repressed	2.25E-02	-2.73
AV162193	Valosin containing protein	Repressed	2.25E-02	-1.16
AV057694	8-oxoguanine DNA-glycosylase 1	Repressed	2.26E-02	-2.28
AV135848	Peroxisome biogenesis factor 19	Repressed	2.26E-02	-1.75
AV059592	Ras homolog gene family, member A	Repressed	2.26E-02	-1.28
AV010030	Adaptor-related protein complex 3, delta 1 subunit	Repressed	2.27E-02	-1.60
BG072686	Agrin	Repressed	2.27E-02	-1.15
AV086597	Checkpoint supressor 1	Repressed	2.27E-02	-1.79
AV016287	CTD (carboxy-terminal domain, RNA polymerase II, polypeptide A) small phosphatase 2	Repressed	2.27E-02	-1.74
BI076595	Cytochrome c oxidase subunit II	Repressed	2.27E-02	-1.22
AV113475	High mobility group box 2	Repressed	2.27E-02	-1.72
BG066212	Leukotriene B4 12-hydroxydehydrogenase	Repressed	2.27E-02	-1.14
AA259766	Pro-opiomelanocortin-alpha	Repressed	2.27E-02	-1.83
AV094522	RIKEN cDNA 2210016L21 gene	Repressed	2.27E-02	-1.77
BG067098	Suppressor of cytokine signaling 7	Repressed	2.27E-02	-1.56
BG066092	Translocase of outer mitochondrial membrane 20 homolog (yeast)	Repressed	2.27E-02	-1.36
BG072696	Tumor protein, translationally-controlled 1	Repressed	2.27E-02	-1.45
AV088048	2,4-dienoyl CoA reductase 1, mitochondrial	Repressed	2.32E-02	-1.77
AV030537	Malate dehydrogenase 1, NAD (soluble)	Repressed	2.32E-02	-1.52
AV010260	Tetratricopeptide repeat domain 3	Repressed	2.32E-02	-1.05
BG075608	Triosephosphate isomerase 1	Repressed	2.32E-02	-1.35
AV014973	Apoptosis inhibitor 5	Repressed	2.34E-02	-1.52

BG064701	Proteasome (prosome, macropain) 26S subunit, ATPase 2	Repressed	2.34E-02	-1.26
W17530	Protein kinase, cAMP dependent, catalytic, alpha	Repressed	2.34E-02	-1.85
BG075384	Ras homolog gene family, member A	Repressed	2.34E-02	-1.19
AV035241	RIKEN cDNA 1600014C10 gene	Repressed	2.34E-02	-1.80
AV093026	RIKEN cDNA 2310076O14 gene	Repressed	2.34E-02	-1.47
AV023199	Selenoprotein W, muscle 1	Repressed	2.34E-02	-1.79
AV053387	Ribosomal protein L13a	Repressed	2.36E-02	-1.43
BG066600	RIKEN cDNA 0610039N19 gene	Repressed	2.36E-02	-1.57
AV034308	Tax1 (human T-cell leukemia virus type I) binding protein 1	Repressed	2.39E-02	-1.44
BG065546	CXXC finger 5	Repressed	2.40E-02	-1.49
AV086337	Keratin complex 1, acidic, gene 15	Repressed	2.40E-02	-1.89
BG075484	Ribosomal protein L7	Repressed	2.40E-02	-1.76
AW556153	Ribosomal protein S6	Repressed	2.40E-02	-1.29
AV071929	3-oxoacid CoA transferase 1	Repressed	2.41E-02	-1.70
AV103051	Caveolin, caveolae protein 1	Repressed	2.41E-02	-1.27
BG065579	Special AT-rich sequence binding protein 1	Repressed	2.41E-02	-1.72
AV055683	ATP-binding cassette, sub-family A (ABC1), member 3	Repressed	2.43E-02	-2.24
AA003086	ADP-ribosylation factor-like 4	Repressed	2.44E-02	-1.80
AW539380	C0119B07	Repressed	2.44E-02	-1.81
BG064652	Macrophage erythroblast attacher	Repressed	2.44E-02	-1.29
BG069760	Cystathionine beta-synthase	Repressed	2.45E-02	-1.21
AV037365	Histocompatibility 2, D region locus 1	Repressed	2.45E-02	-1.53
BG074141	Tyrosine kinase, non-receptor, 2	Repressed	2.45E-02	-1.33
AV018921	Inhibitor of growth family, member 5	Repressed	2.47E-02	-1.72
AW553307	Capping protein (actin filament) muscle Z-line, alpha 2	Repressed	2.50E-02	-1.45
AA259766	Pro-opiomelanocortin-alpha	Repressed	2.51E-02	-1.85
AV039920	ADP-ribosylation factor-like 10C	Repressed	2.52E-02	-1.73
AV093558	Apolipoprotein E	Repressed	2.52E-02	-1.78
AV042417	Cellular nucleic acid binding protein 1	Repressed	2.52E-02	-1.58
BG067932	synthase)	Repressed	2.52E-02	-1.67
AV074444	Restin (Reed-Steinberg cell-expressed intermediate filament-associated protein)	Repressed	2.52E-02	-1.55
AA145778	Cadherin 1	Repressed	2.53E-02	-1.30
AV073394	2	Repressed	2.54E-02	-1.50
BG075767	Prosaposin	Repressed	2.58E-02	-2.15
AV032401	Ribosomal protein S24	Repressed	2.58E-02	-1.47
AV094490	RAS related protein 1b	Repressed	2.59E-02	-1.55

AV078318	RIKEN cDNA 1100001I22 gene	Repressed	2.59E-02	-1.39
AV069862	RIKEN cDNA 2510002A14 gene	Repressed	2.59E-02	-1.57
AK011589	2610028H05	Repressed	2.61E-02	-1.42
AV088144	Casein kinase 1, alpha 1	Repressed	2.61E-02	-1.23
AV037518	Chemokine (C-C motif) ligand 27	Repressed	2.61E-02	-1.83
AV021908	Procollagen, type IV, alpha 2	Repressed	2.63E-02	-1.86
AV085826	Purine rich element binding protein B	Repressed	2.64E-02	-1.08
BG074557	Uncoupling protein 2 (mitochondrial, proton carrier)	Repressed	2.64E-02	-1.16
AA529041	Adenylate cyclase 9	Repressed	2.66E-02	-1.55
AV025718	Annexin A7	Repressed	2.66E-02	-1.17
BG074801	H3139D11	Repressed	2.66E-02	-1.61
BG063545	Kruppel-like factor 6	Repressed	2.66E-02	-1.27
BE685736	Nuclear factor of activated T-cells, cytoplasmic, calcineurin-dependent 3	Repressed	2.67E-02	-1.79
AV170771	Tetratricopeptide repeat domain 3	Repressed	2.67E-02	-3.40
AI596110	Cathepsin B	Repressed	2.68E-02	-1.63
AV084893	Interferon, alpha-inducible protein 27	Repressed	2.68E-02	-1.37
AV082112	Alcohol dehydrogenase 1 (class I)	Repressed	2.71E-02	-2.55
AW909347	RIKEN cDNA 4921504P13 gene	Repressed	2.71E-02	-1.79
AA221832	Four jointed box 1 (Drosophila)	Repressed	2.72E-02	-2.28
AV094789	Homer homolog 2 (Drosophila)	Repressed	2.72E-02	-1.55
AI840666	Acetyl-Coenzyme A dehydrogenase, medium chain	Repressed	2.75E-02	-1.77
AI841308	ATPase, Na+/K+ transporting, beta 1 polypeptide	Repressed	2.75E-02	-2.26
AV015921	RIKEN cDNA 9630058J23 gene	Repressed	2.75E-02	-2.01
BG075650	Golgi SNAP receptor complex member 2	Repressed	2.76E-02	-1.68
AV088036	G elongation factor, mitochondrial 2	Repressed	2.77E-02	-1.53
AV005085	Secretory granule neuroendocrine protein 1, 7B2 protein	Repressed	2.77E-02	-1.26
BG075109	Syntaxin 5A	Repressed	2.77E-02	-1.42
AW552922	AW552922	Repressed	2.78E-02	-1.58
BG067626	Prostaglandin F2 receptor negative regulator	Repressed	2.78E-02	-1.14
C87464	Tumor protein D52	Repressed	2.78E-02	-1.76
AV009567	Ferrochelatase	Repressed	2.79E-02	-1.51
BG065226	Nuclear receptor coactivator 4	Repressed	2.79E-02	-2.69
BG072856	Ribosomal protein L35	Repressed	2.80E-02	-1.41
AI841222	Morf4 family associated protein 1	Repressed	2.81E-02	-1.34
BG074414	Ribosomal protein L22	Repressed	2.81E-02	-1.49
AV103412	Transketolase	Repressed	2.81E-02	-1.37
AV086208	UDP-glucose pyrophosphorylase 2	Repressed	2.81E-02	-1.56

BG071420	Nucleoredoxin	Repressed	2.83E-02	-1.77
AV141372	T-cell leukemia, homeobox 2	Repressed	2.83E-02	-1.52
AV030820	Craniofacial development protein 1	Repressed	2.85E-02	-1.41
AV084131	DNA segment, Chr 10, ERATO Doi 641, expressed	Repressed	2.85E-02	-1.41
AV043303	AV043303	Repressed	2.86E-02	-1.49
AA276279	Cathepsin B	Repressed	2.86E-02	-1.31
AV033293	Expressed sequence AW742319	Repressed	2.86E-02	-1.17
AV086425	IlvB (bacterial acetolactate synthase)-like	Repressed	2.86E-02	-1.50
AV056142	Splicing factor, arginine/serine-rich 5 (SRp40, HRS)	Repressed	2.86E-02	-2.52
BG074900	ATP synthase F0 subunit 6	Repressed	2.87E-02	-1.70
BG073136	ATPase, Na+/K+ transporting, beta 3 polypeptide	Repressed	2.89E-02	-1.51
BG075985	Integral membrane protein 2B	Repressed	2.89E-02	-1.69
BG076208	RIKEN cDNA 3300001P08 gene	Repressed	2.90E-02	-1.70
AV049127	Leukotriene A4 hydrolase	Repressed	2.92E-02	-1.34
C80679	ATPase, H+ transporting, lysosomal accessory protein 1	Repressed	2.93E-02	-1.19
BG073920	Lactate dehydrogenase 2, B chain	Repressed	2.93E-02	-2.13
AV056563	AV056563	Repressed	2.94E-02	-1.28
AW553165	AW553165	Repressed	2.95E-02	-1.18
AI840542	Ribosomal protein S11	Repressed	2.95E-02	-1.55
AV035117	Myosin light chain, regulatory B	Repressed	2.97E-02	-1.33
BG074924	DNA segment, Chr 8, Wayne State University 49, expressed	Repressed	2.99E-02	-2.99
AV072432	Peptidylprolyl isomerase A	Repressed	2.99E-02	-1.99
BG073729	Prolyl 4-hydroxylase, beta polypeptide	Repressed	3.00E-02	-1.33
BG067962	RIKEN cDNA 1110020C13 gene	Repressed	3.00E-02	-1.08
AI840523	UI-M-AM0-adp-b-05-0-UI	Repressed	3.00E-02	-1.55
AV043327	Aldolase 1, A isoform, pseudogene 1	Repressed	3.03E-02	-1.90
AI838414	Karyopherin (importin) alpha 1	Repressed	3.06E-02	-1.32
AV066125	RIKEN cDNA 2010106G01 gene	Repressed	3.06E-02	-1.26
AV105929	Topoisomerase (DNA) I	Repressed	3.06E-02	-1.53
BG072271	RIKEN cDNA 2610510H01 gene	Repressed	3.08E-02	-1.41
BG065012	Tyrosine 3-monooxygenase/tryptophan 5-monooxygenase activation protein, eta polypeptide	Repressed	3.08E-02	-1.41
AV086198	RIKEN cDNA 9130404D14 gene	Repressed	3.09E-02	-1.44
BG068537	Tetratricopeptide repeat domain 12	Repressed	3.09E-02	-1.13
AV086965	Calmodulin 1	Repressed	3.11E-02	-1.21
2300001N02	2300001N02	Repressed	3.14E-02	-1.69

AV103421	Protease (prosome, macropain) 26S subunit, ATPase 1	Repressed	3.14E-02	-1.51
BG063883	Ribosomal protein L7	Repressed	3.14E-02	-1.48
BG075904	RIKEN cDNA 6330578E17 gene	Repressed	3.14E-02	-1.21
AV086114	Isopentenyl-diphosphate delta isomerase	Repressed	3.15E-02	-1.69
AV162289	Polymerase (DNA-directed), delta interacting protein 2	Repressed	3.15E-02	-1.54
AV103528	SWI/SNF related, matrix associated, actin dependent regulator of chromatin, subfamily b, member 1	Repressed	3.15E-02	-1.35
BG063990	gamma	Repressed	3.16E-02	-1.46
AV104104	G protein pathway suppressor 1	Repressed	3.16E-02	-1.27
BG066650	Proteasome (prosome, macropain) 28 subunit, alpha	Repressed	3.16E-02	-1.50
BG073611	Ribosomal protein S6	Repressed	3.16E-02	-1.59
AV032859	AV032859	Repressed	3.17E-02	-1.15
BG065964	Guanine nucleotide binding protein, beta 1	Repressed	3.17E-02	-1.69
BG071218	N-myc downstream regulated gene 2	Repressed	3.17E-02	-1.86
BG071261	Selenoprotein	Repressed	3.17E-02	-1.34
AV043502	Cystatin C	Repressed	3.18E-02	-1.48
BG075899	RIKEN cDNA 5830434P21 gene	Repressed	3.18E-02	-1.69
AV103718	Phenylalanine-tRNA synthetase 2 (mitochondrial)	Repressed	3.19E-02	-1.18
AV060478	Death-associated protein	Repressed	3.22E-02	-1.44
AW559034	Huntingtin interacting protein 2	Repressed	3.23E-02	-1.55
AV083850	2310004F20	Repressed	3.24E-02	-1.59
AW555055	AW555055	Repressed	3.24E-02	-1.26
AV094566	Ubiquitin specific protease 16	Repressed	3.24E-02	-1.68
AV086605	Branched chain ketoacid dehydrogenase E1, alpha polypeptide	Repressed	3.25E-02	-1.76
AV007250	CDC42 small effector 2	Repressed	3.26E-02	-1.32
AV043318	Cut-like 1 (Drosophila)	Repressed	3.26E-02	-1.66
AV093535	Parkinson disease (autosomal recessive, early onset) 7	Repressed	3.26E-02	-1.32
BG076336	RIKEN cDNA 2410042D21 gene	Repressed	3.26E-02	-1.50
BG071504	RIKEN cDNA 2500002L14 gene	Repressed	3.26E-02	-1.04
2200004N05	2200004N05	Repressed	3.27E-02	-1.30
BG076338	F-box only protein 21	Repressed	3.27E-02	-1.89
AI841368	2	Repressed	3.29E-02	-1.32
AV014618	1110055I03	Repressed	3.31E-02	-1.11
AV077886	Cytochrome b-5	Repressed	3.32E-02	-1.29
BG076235	Catalase	Repressed	3.34E-02	-1.99

AA119394	ATP synthase, H+ transporting, mitochondrial F0 complex, subunit b, isoform 1	Repressed	3.35E-02	-2.04
AV083633	2310003J02	Repressed	3.36E-02	-1.56
AI322261	Ribosomal protein L19	Repressed	3.36E-02	-1.50
BG074111	ATP synthase, H+ transporting, mitochondrial F1 complex, gamma polypeptide 1	Repressed	3.37E-02	-1.45
AV094650	Developmentally regulated GTP binding protein 1	Repressed	3.37E-02	-1.52
BG074363	Phosphatidylinositol glycan, class Q	Repressed	3.37E-02	-1.25
BG065070	Eukaryotic translation initiation factor 4, gamma 2	Repressed	3.39E-02	-1.41
AV022782	Dynein, cytoplasmic, heavy chain 1	Repressed	3.40E-02	-1.55
BG068553	RIKEN cDNA 1110003E01 gene	Repressed	3.41E-02	-1.53
AV056620	Ropporin 1-like	Repressed	3.41E-02	-1.73
BG067823	Aminopeptidase puromycin sensitive	Repressed	3.43E-02	-1.19
AW555492	AW555492	Repressed	3.43E-02	-1.06
AV104548	Splicing factor 3b, subunit 1	Repressed	3.43E-02	-1.38
BG069067	RIKEN cDNA 4121402D02 gene	Repressed	3.44E-02	-1.76
BG071684	F-box only protein 3	Repressed	3.45E-02	-1.42
AV083325	Inositol hexaphosphate kinase 1	Repressed	3.45E-02	-1.45
AV093793	Ribosomal protein L24	Repressed	3.46E-02	-1.47
AV048822	Ribosomal protein S6	Repressed	3.46E-02	-1.45
BG074808	SEC31-like 1 (S. cerevisiae)	Repressed	3.46E-02	-1.31
BG071789	Signaling lymphocytic activation molecule family member 1	Repressed	3.46E-02	-1.55
BG064584	Translin	Repressed	3.46E-02	-1.65
AV072536	2200004B21	Repressed	3.47E-02	-1.37
AV088033	Cytochrome c oxidase subunit VIIa polypeptide 2-like	Repressed	3.47E-02	-1.81
AA656056	Nonagouti	Repressed	3.47E-02	-1.74
BG075966	Tetraspanin 9	Repressed	3.47E-02	-1.88
AV073401	Transgelin	Repressed	3.48E-02	-1.15
AV032329	Integral membrane protein 2B	Repressed	3.50E-02	-1.98
BG076352	RIKEN cDNA 1700020I14 gene	Repressed	3.50E-02	-1.84
AV135866	WD repeat domain 33	Repressed	3.50E-02	-1.75
BG076355	3	Repressed	3.51E-02	-1.23
AW549924	AW549924	Repressed	3.52E-02	-1.30
AV090250	Glucocorticoid induced transcript 1	Repressed	3.53E-02	-1.43
BG064832	RIKEN cDNA 4930542G03 gene	Repressed	3.53E-02	-1.64
BG075328	Ring finger protein 7	Repressed	3.53E-02	-1.18
AV050843	2	Repressed	3.53E-02	-1.67
BG073995	H3129F06	Repressed	3.58E-02	-1.66

BG065146	Ribosomal protein L15	Repressed	3.58E-02	-1.43
BG066223	Actin related protein 2/3 complex, subunit 2	Repressed	3.60E-02	-1.92
AV069980	Beta-2 microglobulin	Repressed	3.60E-02	-1.63
AV008661	Monoglyceride lipase	Repressed	3.60E-02	-2.15
AA221782	RIKEN cDNA 9330164J24 gene	Repressed	3.60E-02	-1.60
AV104072	Sterol carrier protein 2, liver	Repressed	3.60E-02	-1.65
AV088602	WD repeat domain 23	Repressed	3.60E-02	-1.33
AV057772	Myc induced nuclear antigen	Repressed	3.63E-02	-1.49
AV033666	Glucose regulated protein	Repressed	3.67E-02	-1.55
AV103984	Leptin receptor overlapping transcript	Repressed	3.67E-02	-1.31
BG075528	Leucine-rich repeats and immunoglobulin-like domains 3	Repressed	3.67E-02	-1.27
BG075688	Parkinson disease (autosomal recessive, early onset) 7	Repressed	3.67E-02	-1.26
AV014782	Programmed cell death 4	Repressed	3.67E-02	-1.84
AV038329	RIKEN cDNA 1810045K07 gene	Repressed	3.67E-02	-1.52
AI841357	Solute carrier family 25 (mitochondrial carrier, adenine nucleotide translocator), member 4	Repressed	3.67E-02	-1.33
AV031080	Ubiquitin-like 3	Repressed	3.67E-02	-1.64
BG073760	DEAD (Asp-Glu-Ala-Asp) box polypeptide 5	Repressed	3.73E-02	-1.33
BG067497	Peroxiredoxin 1	Repressed	3.73E-02	-1.24
AV095054	Ribosomal protein L9	Repressed	3.75E-02	-1.63
BG075361	Low density lipoprotein receptor-related protein 1	Repressed	3.76E-02	-1.93
BG064914	Malate dehydrogenase 1, NAD (soluble)	Repressed	3.76E-02	-1.74
AV041580	High mobility group box 2	Repressed	3.80E-02	-1.34
BG073960	Eukaryotic translation elongation factor 2	Repressed	3.81E-02	-2.10
AV073472	Olfactomedin 1	Repressed	3.82E-02	-1.53
BG073636	Proteasome (prosome, macropain) 28 subunit, alpha	Repressed	3.82E-02	-1.47
AW551176	AW551176	Repressed	3.85E-02	-1.24
BG070614	RIKEN cDNA 2610009E16 gene	Repressed	3.85E-02	-1.50
AV053280	Twisted gastrulation homolog 1 (Drosophila)	Repressed	3.85E-02	-1.55
BG073707	Ubiquitin C	Repressed	3.85E-02	-1.56
2310041F23	2310041F23	Repressed	3.88E-02	-1.57
BG072819	Laminin receptor 1 (ribosomal protein SA)	Repressed	3.88E-02	-1.27
AV135760	Acyl-Coenzyme A dehydrogenase family, member 9	Repressed	3.89E-02	-1.12
BG069998	Beta-site APP cleaving enzyme 1	Repressed	3.89E-02	-2.16
AU022809	Deleted in polyposis 1	Repressed	3.89E-02	-1.55
AV103186	Mortality factor 4 like 2	Repressed	3.89E-02	-1.36
BG067898	ATP synthase F0 subunit 6	Repressed	3.90E-02	-1.49

AV061111	Gamma-glutamyl hydrolase	Repressed	3.90E-02	-1.53
AV039137	Nitrilase 1	Repressed	3.90E-02	-1.82
AV037994	Protein phosphatase 2 (formerly 2A), regulatory subunit B, alpha	Repressed	3.90E-02	-1.69
BG073219	Ribosomal protein S26	Repressed	3.90E-02	-1.25
AV041527	RIKEN cDNA 2600001J17 gene	Repressed	3.90E-02	-1.46
BG076192	RIKEN cDNA 2810021B07 gene	Repressed	3.90E-02	-1.70
BG066355	Stromal cell derived factor 4	Repressed	3.90E-02	-1.43
AA163986	Guanosine diphosphate (GDP) dissociation inhibitor 3	Repressed	3.91E-02	-1.48
BG064915	Proteasome (prosome, macropain) 26S subunit, non-ATPase, 13	Repressed	3.91E-02	-1.54
AV010913	SPFH domain family, member 2	Repressed	3.91E-02	-1.46
AV059610	AV059610	Repressed	3.93E-02	-1.28
BG064713	RIKEN cDNA 2600011C06 gene	Repressed	3.93E-02	-1.47
BG076110	RIKEN cDNA 4921506J03 gene	Repressed	3.93E-02	-1.38
AV018613	RNA binding motif protein 13	Repressed	3.93E-02	-1.58
BG075001	2	Repressed	3.93E-02	-1.39
AA407888	Desmoplakin	Repressed	3.95E-02	-1.33
AV009996	HLA-B-associated transcript 3	Repressed	3.95E-02	-1.34
BG073982	Makorin, ring finger protein, 1	Repressed	3.95E-02	-1.54
BG068996	Phosphatidylinositol 3-kinase, regulatory subunit, polypeptide 1 (p85 alpha)	Repressed	3.96E-02	-1.55
BG074849	Myotrophin	Repressed	3.98E-02	-1.27
AV002933	Palmitoyl-protein thioesterase 2	Repressed	3.98E-02	-1.67
AV022767	Pleiotrophin	Repressed	3.98E-02	-1.68
BG065098	CDC-like kinase 1	Repressed	3.99E-02	-1.61
BG072080	Ring finger protein 44	Repressed	4.00E-02	-1.39
AV031224	Coatomer protein complex, subunit gamma	Repressed	4.02E-02	-1.18
AV103159	Tubulin, beta 5	Repressed	4.02E-02	-1.25
BG074759	Cathepsin D	Repressed	4.04E-02	-1.16
BB565248	2300001F12	Repressed	4.05E-02	-1.49
AV084119	ADP-ribosylation factor-like 4	Repressed	4.06E-02	-1.73
AV052783	Anaphase-promoting complex subunit 5	Repressed	4.09E-02	-1.17
BG063432	DEAD (Asp-Glu-Ala-Asp) box polypeptide 48	Repressed	4.10E-02	-1.35
AV170924	RIKEN cDNA 2510048O06 gene	Repressed	4.10E-02	-1.30
AV114441	Translin	Repressed	4.11E-02	-1.14
BG068010	Ring finger protein 2	Repressed	4.13E-02	-1.37
AI841379	Cyclin D3	Repressed	4.15E-02	-1.31
AW543826	RAB2, member RAS oncogene family	Repressed	4.15E-02	-1.16
AV040590	Sorbitol dehydrogenase 1	Repressed	4.15E-02	-1.29
AW539669	Sequestosome 1	Repressed	4.17E-02	-1.48

AA146030	Sterol carrier protein 2, liver	Repressed	4.17E-02	-1.37
AW701842	Tial1 cytotoxic granule-associated RNA binding protein-like 1	Repressed	4.18E-02	-1.64
BG074678	A430091O22Rik	Repressed	4.19E-02	-1.37
AV017472	Ferritin heavy chain 1	Repressed	4.19E-02	-1.31
AV010091	Iduronidase, alpha-L-	Repressed	4.19E-02	-1.47
AV031462	Malignant T cell amplified sequence 1	Repressed	4.19E-02	-1.59
AV036516	Nucleophosmin 1	Repressed	4.19E-02	-1.57
AV134917	REST co-repressor 2	Repressed	4.19E-02	-1.91
AV021056	Supervillin	Repressed	4.19E-02	-1.31
AI840878	Homeobox only domain	Repressed	4.20E-02	-2.16
BG073880	Adiponectin receptor 1	Repressed	4.21E-02	-1.16
AW546647	Pam, highwire, rpm 1	Repressed	4.21E-02	-1.85
BG065652	Cystatin C	Repressed	4.22E-02	-1.46
AV104549	Osteoclast stimulating factor 1	Repressed	4.23E-02	-1.38
BG073607	Ribosomal protein L4	Repressed	4.23E-02	-1.88
AI838603	Ephrin A5	Repressed	4.24E-02	-1.74
BG071253	CTD (carboxy-terminal domain, RNA polymerase II, polypeptide A) small phosphatase 1	Repressed	4.26E-02	-1.05
AA684233	Matrix metalloproteinase 12	Repressed	4.28E-02	-1.90
BG075206	Ribosomal protein L6	Repressed	4.28E-02	-1.34
AU021796	Churchill domain containing 1	Repressed	4.31E-02	-1.37
AV030672	Influenza virus NS1A binding protein	Repressed	4.32E-02	-1.30
AV112756	RIKEN cDNA 2600011C06 gene	Repressed	4.32E-02	-1.85
AV171117	U1 small nuclear ribonucleoprotein polypeptide A	Repressed	4.32E-02	-1.27
BG064816	Acidic ribosomal phosphoprotein P0	Repressed	4.33E-02	-1.44
AV025785	AV025785	Repressed	4.37E-02	-1.34
AV013738	Bone gamma carboxyglutamate protein 1	Repressed	4.37E-02	-1.44
BG066564	Vacuolar protein sorting 24 (yeast)	Repressed	4.37E-02	-1.38
AU016579	AU016579	Repressed	4.38E-02	-1.21
BG073511	Cellular nucleic acid binding protein 1	Repressed	4.40E-02	-1.37
AV031166	Integral membrane protein 2B	Repressed	4.40E-02	-2.22
BG074739	Syndecan 4	Repressed	4.41E-02	-1.78
C77236	C77236	Repressed	4.43E-02	-1.42
AU044044	Membrane interacting protein of RGS16	Repressed	4.43E-02	-1.36
AA240738	Rho GTPase activating protein 12	Repressed	4.43E-02	-1.45
AV068654	RIKEN cDNA 1110005A23 gene	Repressed	4.43E-02	-1.75
BG071783	Spastic paraplegia 21 homolog (human)	Repressed	4.43E-02	-1.52
AV005056	Phospholipase D3	Repressed	4.46E-02	-1.41
AI851011	Spinocerebellar ataxia 2 homolog (human)	Repressed	4.53E-02	-1.41

BG076340	CDNA sequence BC003324	Repressed	4.58E-02	-1.44
AV058809	N-terminal Asn amidase	Repressed	4.58E-02	-1.42
AW548440	Ribosomal protein S15	Repressed	4.58E-02	-3.10
AV048780	Stromal cell derived factor 4	Repressed	4.58E-02	-1.35
AV112179	AV112179	Repressed	4.59E-02	-1.65
AA153173	Dystroglycan 1	Repressed	4.59E-02	-1.77
AV084172	Ornithine aminotransferase	Repressed	4.59E-02	-2.03
BG073052	Prothymosin alpha	Repressed	4.59E-02	-1.39
AV030530	Solute carrier family 25 (mitochondrial carrier, palmitoylcarnitine transporter), member 29	Repressed	4.59E-02	-1.81
AV061292	Ubiquitin specific protease 16	Repressed	4.59E-02	-1.84
BG073955	Swi/SNF related matrix associated, actin dependent regulator of chromatin, subfamily a-like 1	Repressed	4.60E-02	-2.07
BG073959	Eukaryotic translation elongation factor 2	Repressed	4.62E-02	-2.02
AV015067	Non-catalytic region of tyrosine kinase adaptor protein 1	Repressed	4.62E-02	-1.91
BG068883	RIKEN cDNA 4833439L19 gene	Repressed	4.62E-02	-1.25
BG064520	START domain containing 7	Repressed	4.62E-02	-1.35
AV073579	Cyclin L1	Repressed	4.63E-02	-1.45
BG068899	H3070E06	Repressed	4.63E-02	-1.24
BG073686	Ribosomal protein, large, P1	Repressed	4.63E-02	-1.21
AV084245	Synaptophysin-like protein	Repressed	4.63E-02	-1.23
AV095041	Arrestin domain containing 4	Repressed	4.65E-02	-1.59
AI840764	AI840764	Repressed	4.69E-02	-1.27
AA139719	Electron transferring flavoprotein, alpha polypeptide	Repressed	4.69E-02	-1.90
AI840657	Cytochrome c oxidase subunit VIIa polypeptide 2-like	Repressed	4.70E-02	-1.66
AV050158	RIKEN cDNA 1810047C23 gene	Repressed	4.72E-02	-1.77
BG076278	Yippee-like 3 (Drosophila)	Repressed	4.72E-02	-1.60
BG074973	Growth arrest specific 1	Repressed	4.73E-02	-1.10
AV095179	Guanine nucleotide binding protein, beta 2, related sequence 1	Repressed	4.79E-02	-1.42
AV017692	Kelch-like 7 (Drosophila)	Repressed	4.80E-02	-1.89
BG072862	Ribosomal protein L23a	Repressed	4.80E-02	-1.29
AI840610	UI-M-AM0-adq-d-06-0-UI	Repressed	4.82E-02	-1.58
BG064745	Phosphoglycerate kinase 1	Repressed	4.88E-02	-1.53
BG066682	Expressed sequence AA536749	Repressed	4.89E-02	-1.19
AV052727	Ring finger protein 7	Repressed	4.89E-02	-1.18
BI076582	C0196G08	Repressed	4.91E-02	-1.41
AV061968	Alcohol dehydrogenase 6A (class V)	Repressed	4.92E-02	-1.83

AW549974	AW549974	Repressed	4.92E-02	-1.37
AV078433	Membrane interacting protein of RGS16	Repressed	4.92E-02	-1.93
BG074718	Procollagen, type IV, alpha 2	Repressed	4.92E-02	-1.70
BG074727	Sema domain, immunoglobulin domain (Ig), transmembrane domain (TM) and short cytoplasmic domain, (semaphorin) 4G	Repressed	4.96E-02	-2.06
BG074831	RIKEN cDNA 0610031J06 gene	Repressed	4.97E-02	-1.08
AU022217	Epoxide hydrolase 2, cytoplasmic	Repressed	4.97E-02	-2.24
AV037205	DNA segment, Chr 10, ERATO Doi 516, expressed	Repressed	4.97E-02	-1.63
AV030790	Phosphatidylethanolamine binding protein	Repressed	4.97E-02	-1.22
AV086800	Actin, alpha 1, skeletal muscle	Repressed	4.97E-02	-1.74
AV141694	Signal recognition particle 54	Repressed	4.97E-02	-1.77
BG066549	Ubiquitin-conjugating enzyme E2L 3	Repressed	4.97E-02	-1.27
BG072707	Spermidine/spermine N1-acetyl transferase 1	Repressed	4.97E-02	-1.62
AW545246	Renal tumor antigen	Repressed	4.97E-02	-1.66
C79165	Toll interacting protein	Repressed	4.97E-02	-1.54
AV112669	RIKEN cDNA 2610014I16 gene	Repressed	4.97E-02	-2.14
AV017314	OTU domain, ubiquitin aldehyde binding 1	Repressed	4.97E-02	-1.96
BG066093	PRA1 domain family 2	Repressed	4.97E-02	-1.48
AV002407	Splicing factor, arginine/serine-rich 5 (SRp40, HRS)	Repressed	4.97E-02	-1.58
BG064090	ketoacyl-Coenzyme A thiolase/enoyl-Coenzyme A hydratase (trifunctional protein), beta subunit	Repressed	4.97E-02	-1.40
AV093499	Thymosin, beta 4, X chromosome	Repressed	4.97E-02	-1.86
BG064581	RIKEN cDNA 4921506J03 gene	Repressed	4.97E-02	-1.71
AV085874	UDP-glucose pyrophosphorylase 2	Repressed	4.97E-02	-1.53
AV031173	Prion protein	Repressed	4.97E-02	-1.30
BG065052	Receptor-like tyrosine kinase	Repressed	4.97E-02	-1.90
AI838731	DNA segment, Chr 17, Wayne State University 92, expressed	Repressed	4.97E-02	-1.34
AV052097	RIKEN cDNA 1110032D12 gene	Repressed	4.97E-02	-1.48
AV162437	Expressed sequence AU019823	Repressed	4.97E-02	-2.23
BG064276	Reticulon 4	Repressed	4.97E-02	-1.59
AV078181	RIKEN cDNA 1200009B18 gene	Repressed	4.97E-02	-2.63
AV016436	G protein-coupled receptor 133	Repressed	4.97E-02	-1.25
BG075951	Myotubularin related protein 3	Repressed	4.97E-02	-1.64
AV094520	HpaII tiny fragments locus 9c	Repressed	4.97E-02	-1.94
AV103516	H19 fetal liver mRNA	Repressed	4.97E-02	-1.69
AV041910	LanC (bacterial lantibiotic synthetase component C)-like 1	Repressed	4.97E-02	-1.33

AU040633	Rho guanine nucleotide exchange factor (GEF) 11	Repressed	4.97E-02	-1.52
BG070487	Acetyl-Coenzyme A acetyltransferase 1	Repressed	4.97E-02	-1.58
AV104410	SET translocation	Repressed	4.98E-02	-1.25
AV009183	CDNA sequence BC043098	Repressed	4.98E-02	-1.55
AV095043	Tumor protein, translationally-controlled 1	Repressed	4.98E-02	-1.53
BG073583	Proteasome (prosome, macropain) 26S subunit, non-ATPase, 4	Repressed	4.98E-02	-1.36
BG068850	E1A binding protein p400	Repressed	4.98E-02	-1.35
BG067793	RNA binding motif protein 18	Repressed	4.98E-02	-1.83
AV050135	RIKEN cDNA 4833420G17 gene	Repressed	4.98E-02	-1.49
BG073105	Ribosomal protein S17	Repressed	4.98E-02	-1.33
AI838295	Ribosomal protein S9	Repressed	4.98E-02	-1.55
AV090034	Testis expressed gene 2	Repressed	4.98E-02	-1.53
AV093667	Proliferating cell nuclear antigen	Repressed	4.98E-02	-1.59
AV060145	Ornithine decarboxylase antizyme 2	Repressed	4.98E-02	-1.53
BG073867	RIKEN cDNA 2900097C17 gene	Repressed	4.98E-02	-1.34
BG073526	Prostate tumor over expressed gene 1	Repressed	4.98E-02	-1.50
AW555660	AW555660	Repressed	4.98E-02	-1.62
AV057503	Serine protease inhibitor, Kunitz type 2	Repressed	4.98E-02	-1.40
AV103300	Sarcoma amplified sequence	Repressed	4.98E-02	-1.44
BG067648	H3056F09	Repressed	4.98E-02	-3.04
AV065485	Malate dehydrogenase 1, NAD (soluble)	Repressed	4.98E-02	-1.48
BG069789	RIKEN cDNA 1110021H02 gene	Repressed	4.98E-02	-1.56
AV094961	Ribosomal protein S6	Repressed	4.98E-02	-2.18
BG071381	Neurocalcin delta	Repressed	4.98E-02	-1.48
BG075142	Splicing factor, arginine/serine-rich 2 (SC-35)	Repressed	4.98E-02	-1.57
AV104528	Adenylate kinase 2	Repressed	4.98E-02	-1.25
AV086153	Von Hippel-Lindau binding protein 1	Repressed	4.98E-02	-2.13
BG071219	Histocompatibility 2, D region locus 1	Repressed	4.98E-02	-1.04
BG072625	Ribosomal protein L19	Repressed	4.98E-02	-1.45
BG070402	Williams-Beuren syndrome chromosome region 1 homolog (human)	Repressed	4.98E-02	-1.34
BG065202	Ribosomal protein S15a	Repressed	4.98E-02	-1.34
AV083977	Coiled-coil-helix-coiled-coil-helix domain containing 3	Repressed	4.98E-02	-1.66
AV051660	Capping protein (actin filament) muscle Z-line, alpha 2	Repressed	4.98E-02	-1.53
BG070286	Capping protein (actin filament) muscle Z-line, alpha 2	Repressed	4.98E-02	-1.60
BG064700	Proteasome (prosome, macropain) 26S subunit, ATPase 2	Repressed	4.98E-02	-2.37

AV084024	Armadillo repeat gene deleted in velo-cardio-facial syndrome	Repressed	4.98E-02	-1.66
AV052634	N-myc downstream regulated gene 1	Repressed	4.99E-02	-1.61
BG065703	Lin 10 homolog (C. elegans)	Repressed	4.99E-02	-1.41
AV170828	Secreted acidic cysteine rich glycoprotein	Repressed	4.99E-02	-1.52
BG073775	Ribosomal protein L26	Repressed	4.99E-02	-1.58
BG064856	Tripeptidyl peptidase II	Repressed	4.99E-02	-1.90
AV036580	Gelsolin	Repressed	4.99E-02	-1.36
AV021152	RAB18, member RAS oncogene family	Repressed	4.99E-02	-1.42
BG074964	Steroid receptor RNA activator 1	Repressed	4.99E-02	-1.32
AV095102	2	Repressed	4.99E-02	-1.74
BG065087	Nucleolar protein family A, member 2	Repressed	4.99E-02	-1.85
BG075721	COMM domain containing 7	Repressed	4.99E-02	-1.49
BG064935	ADP-ribosylarginine hydrolase	Repressed	4.99E-02	-1.52
AV086052	Syntaxin 5A	Repressed	4.99E-02	-1.56
BG072486	MAF1 homolog (yeast)	Repressed	4.99E-02	-1.40
AV094553	Ubiquitin specific protease 14	Repressed	4.99E-02	-1.35
AV085666	Tumor-associated calcium signal transducer 1	Repressed	4.99E-02	-1.36
AI851052	THUMP domain containing 1	Repressed	4.99E-02	-2.00
BG075132	Expressed sequence AW555464	Repressed	4.99E-02	-1.27
BG076120	SMAD specific E3 ubiquitin protein ligase 1	Repressed	4.99E-02	-1.54
AA004048	Proteasome (prosome, macropain) 26S subunit, non-ATPase, 4	Repressed	4.99E-02	-1.82
BG074875	RIKEN cDNA 5031439G07 gene	Repressed	4.99E-02	-1.18
BG071645	RIKEN cDNA 0610040D20 gene	Repressed	4.99E-02	-1.59
AI838613	Epithelial membrane protein 1	Repressed	4.99E-02	-1.49
AV039195	Calcium/calmodulin -dependent protein kinase II gamma	Repressed	4.99E-02	-1.67
AV042402	ADP-ribosylation factor 1	Repressed	4.99E-02	-1.09
AI837820	Signal recognition particle 68	Repressed	4.99E-02	-1.47
BG073049	RIKEN cDNA C030011O14 gene	Repressed	4.99E-02	-1.51
AI847544	GPI-anchored membrane protein 1	Repressed	4.99E-02	-1.24
AV084141	2	Repressed	4.99E-02	-1.66
AI854102	Farnesyltransferase, CAAX box, alpha	Repressed	4.99E-02	-1.48
BG063515	Ferritin heavy chain 1	Repressed	5.00E-02	-1.53
BG065981	RIKEN cDNA 2900064A13 gene	Repressed	5.00E-02	-1.19
AW544139	C0178C03	Repressed	5.00E-02	-1.48
BG073659	H3126E11	Repressed	5.00E-02	-1.24
AV108774	Prolylcarboxypeptidase (angiotensinase C)	Repressed	5.00E-02	-1.76
BG073016	Membrane associated guanylate kinase, WW and PDZ domain containing 2	Repressed	5.00E-02	-2.49
AV033809	Aldolase 3, C isoform	Repressed	5.00E-02	-1.66

AV025325	RIKEN cDNA A630078A22 gene	Repressed	5.00E-02	-2.39
BG072994	Hydroxysteroid (17-beta) dehydrogenase 4	Repressed	5.00E-02	-2.53
AV042103	Coiled-coil-helix-coiled-coil-helix domain containing 6	Repressed	5.00E-02	-1.96
AV032283	Mitogen activated protein kinase 10	Repressed	5.00E-02	-4.23
AV015100	T-cell receptor beta, variable 13	Repressed	5.00E-02	-2.61
AV015499	ADP-ribosylation factor-like 6 interacting protein 2	Repressed	5.00E-02	-1.60
AA059718	Polymerase (DNA directed), beta	Repressed	5.00E-02	-1.16
BG074707	Activity-dependent neuroprotective protein	Repressed	5.00E-02	-3.33
AV086733	Acetyl-Coenzyme A dehydrogenase, medium chain	Repressed	5.00E-02	-1.97
BG075648	Serine (or cysteine) proteinase inhibitor, clade G, member 1	Repressed	5.00E-02	-1.69
AI838562	SMC6 structural maintenance of chromosomes 6-like 1 (yeast)	Repressed	5.00E-02	-2.09
AV018210	Ribosomal protein S21	Repressed	5.00E-02	-1.31
BG074098	Nucleoplasmin 3	Induced	1.14E-09	+3.09
AA409339	Testis expressed gene 261	Induced	3.91E-09	+1.69
BG073680	Cell division cycle 34 homolog (S. cerevisiae)	Induced	1.33E-07	+1.75
BG064948	Spindle pole body component 24 homolog (S. cerevisiae)	Induced	3.29E-07	+2.96
BG071427	Dicarbonyl L-xylulose reductase	Induced	9.13E-07	+2.54
AV084455	Dolichyl-phosphate mannosyltransferase polypeptide 3	Induced	1.57E-05	+2.06
BG075840	RIKEN cDNA 2010100O12 gene	Induced	2.46E-05	+1.66
AV086746	Small proline-rich protein 1B	Induced	3.32E-05	+3.98
BG074979	Expressed sequence AA408556	Induced	5.33E-05	+1.99
AV095233	Tubulin, gamma 1	Induced	1.72E-04	+1.49
AV094905	Differentially expressed in FDCP 6	Induced	3.64E-04	+1.75
AA116654	Dicarbonyl L-xylulose reductase	Induced	4.34E-04	+2.70
BG069364	Apoptosis antagonizing transcription factor	Induced	5.88E-04	+1.44
BG065165	Thymopoietin	Induced	6.32E-04	+1.86
BG071245	Carbonyl reductase 2	Induced	6.70E-04	+1.49
BG070554	Protein tyrosine phosphatase 4a3	Induced	7.90E-04	+3.48
BG072793	Lymphocyte antigen 6 complex, locus A	Induced	8.94E-04	+2.84
AV054851	ST6 (alpha-N-acetyl-neuraminyl-2,3-beta-galactosyl-1,3)-N-acetylgalactosaminide alpha-2,6-sialyltransferase 2	Induced	9.23E-04	+2.25
AA063844	ATPase, Na+/K+ transporting, alpha 1 polypeptide	Induced	9.24E-04	+1.92
AV048481	Fractured callus expressed transcript 1	Induced	9.69E-04	+1.54

BG064905	Nucleolar protein 1	Induced	9.69E-04	+2.74
AA163925	B-cell leukemia/lymphoma 3	Induced	1.18E-03	+2.31
AV025104	Expressed sequence AI451006	Induced	1.23E-03	+2.54
BG067558	Microtubule-associated protein, RP/EB family, member 1	Induced	1.44E-03	+1.66
BG063104	Peptidylprolyl isomerase D (cyclophilin D)	Induced	1.49E-03	+6.19
AV093677	Polymerase (DNA directed), delta 2, regulatory subunit	Induced	1.51E-03	+1.79
AA163512	AA163512	Induced	1.71E-03	+1.45
AU022992	Centaurin, delta 1	Induced	1.78E-03	+1.46
BG068047	Cytokine induced apoptosis inhibitor 1	Induced	1.80E-03	+1.66
AV056264	Solute carrier family 19 (sodium/hydrogen exchanger), member 1	Induced	2.31E-03	+1.93
BG075470	Ash2 (absent, small, or homeotic)-like (Drosophila)	Induced	2.45E-03	+1.32
BG064927	AE binding protein 2	Induced	2.53E-03	+1.71
AI854095	CDNA sequence AF155546	Induced	2.53E-03	+2.71
AV089779	Myocyte enhancer factor 2B	Induced	2.53E-03	+1.78
BG063938	Aurora kinase A	Induced	2.69E-03	+2.66
AA080218	GLI-Kruppel family member HKR3	Induced	2.69E-03	+2.43
BG070634	Polymerase (RNA) II (DNA directed) polypeptide I	Induced	2.69E-03	+1.39
BG075671	Nuclear transcription factor-Y gamma	Induced	2.85E-03	+1.51
BG074358	Ngfi-A binding protein 1	Induced	3.10E-03	+2.04
AV090011	Hypoxia-inducible factor 1, alpha subunit inhibitor	Induced	3.18E-03	+2.54
AV008153	Protein tyrosine phosphatase, receptor type, f polypeptide (PTPRF), interacting protein (liprin), alpha 4	Induced	3.55E-03	+1.82
BG073790	RIKEN cDNA 2700094K13 gene	Induced	3.55E-03	+1.41
AA125268	Tnf receptor-associated factor 7	Induced	3.61E-03	+1.66
BG065212	Polymerase (DNA directed), delta 2, regulatory subunit	Induced	3.74E-03	+1.65
BG067130	Protein phosphatase 2, regulatory subunit B (B56), gamma isoform	Induced	3.75E-03	+1.99
AV085682	(human)	Induced	4.30E-03	+1.72
AV057471	RIKEN cDNA 1810009K13 gene	Induced	4.46E-03	+1.97
BG072013	RIKEN cDNA 2410003K15 gene	Induced	4.52E-03	+1.60
AV026345	Mitogen activated protein kinase kinase 7	Induced	4.56E-03	+1.99
AV058496	Histone 1, H4h	Induced	4.68E-03	+1.79
AV012325	Pyruvate dehydrogenase kinase, isoenzyme 3	Induced	4.69E-03	+2.03
AV103844	Arginine vasopressin-induced 1	Induced	4.76E-03	+1.90

AV104326	Immediate early response 3	Induced	4.77E-03	+2.46
BG063066	Heparan sulfate (glucosamine) 3-O-sulfotransferase 1	Induced	4.87E-03	+3.60
AV095081	Cyclin E1	Induced	4.93E-03	+1.69
BG075690	RIKEN cDNA B430218L07 gene	Induced	5.29E-03	+1.67
AA061454	Sec61 alpha 1 subunit (S. cerevisiae)	Induced	5.29E-03	+1.81
BG071714	Apoptotic chromatin condensation inducer 1	Induced	5.49E-03	+1.43
BG067014	Carbonyl reductase 3	Induced	5.52E-03	+1.81
AV043571	Exosome component 5	Induced	5.52E-03	+1.67
AV170878	Rhotekin	Induced	5.52E-03	+1.49
BG075773	RIKEN cDNA 2010109N14 gene	Induced	5.67E-03	+1.54
BG064696	RIKEN cDNA 2310009B15 gene	Induced	6.27E-03	+1.84
AV048928	RIKEN cDNA 1810008A18 gene	Induced	6.74E-03	+2.07
AV094547	CD68 antigen	Induced	6.75E-03	+1.59
AI841376	Protein phosphatase 5, catalytic subunit	Induced	6.80E-03	+2.12
BG068304	RIKEN cDNA 2310061C15 gene	Induced	7.06E-03	+1.41
BG063000	Arginine vasopressin-induced 1	Induced	7.21E-03	+2.19
BG068556	Phosphatidylinositol glycan, class F	Induced	7.56E-03	+1.53
BG070559	Integrin beta 4 binding protein	Induced	8.06E-03	+2.63
AV023921	RIKEN cDNA 2510039O18 gene	Induced	8.34E-03	+1.53
BG070781	RIKEN cDNA 1110001A12 gene	Induced	8.42E-03	+1.88
AV035186	Calcium regulated heat stable protein 1	Induced	8.59E-03	+2.43
BG065292	Mitochondrial ribosomal protein S11	Induced	8.73E-03	+2.44
AV083936	Tumor differentially expressed 2-like	Induced	8.73E-03	+2.12
AV103724	N-acetyl galactosaminidase, alpha	Induced	8.79E-03	+1.70
BG064068	G patch domain containing 4	Induced	9.07E-03	+2.28
AV094930	alacrimia	Induced	9.20E-03	+2.04
AV051644	RNA, U17d small nucleolar	Induced	9.20E-03	+1.76
AA521703	Janus kinase 2	Induced	9.21E-03	+2.55
BG074668	Minichromosome maintenance deficient 2 mitotin (S. cerevisiae)	Induced	9.22E-03	+1.75
AW539204	Eukaryotic translation initiation factor 4 gamma, 3	Induced	9.25E-03	+1.69
AV038265	Lymphocyte antigen 6 complex, locus A	Induced	9.25E-03	+3.30
AV112830	RIKEN cDNA 1300002C08 gene	Induced	9.54E-03	+1.29
AA138098	Sec61 beta subunit	Induced	9.54E-03	+2.06
BG067547	RIKEN cDNA 2600010E01 gene	Induced	9.99E-03	+4.99
AV088125	RIKEN cDNA 1110007C05 gene	Induced	1.03E-02	+1.81
AU021079	Transmembrane protein induced by tumor necrosis factor alpha	Induced	1.07E-02	+1.67
AV085647	RIKEN cDNA 9130227C08 gene	Induced	1.09E-02	+1.36
BG070527	CDC28 protein kinase regulatory subunit 2	Induced	1.10E-02	+1.46

BG066921	WD repeat domain 50	Induced	1.10E-02	+1.38
BG064577	DNA segment, Chr 2, Wayne State University 81, expressed	Induced	1.14E-02	+1.68
BG069886	Splicing factor 3b, subunit 4	Induced	1.14E-02	+2.08
BG064839	WD repeat domain 1	Induced	1.14E-02	+2.64
BG071448	General transcription factor IIIC, polypeptide 5	Induced	1.17E-02	+1.75
AV055813	1810036N04	Induced	1.18E-02	+1.38
BG074776	DNA segment, Chr 5, ERATO Doi 585, expressed	Induced	1.19E-02	+1.90
AV015860	Ubiquitin-like, containing PHD and RING finger domains, 1	Induced	1.19E-02	+1.53
BG065055	Minichromosome maintenance deficient 3 (S. cerevisiae)	Induced	1.24E-02	+1.59
AV051503	STE20-like kinase (yeast)	Induced	1.26E-02	+1.92
BG071781	Expressed sequence AU044856	Induced	1.29E-02	+1.90
AV084363	Metallothionein 2	Induced	1.30E-02	+1.81
BG063978	Capping protein (actin filament), gelsolin-like	Induced	1.34E-02	+1.56
BG074275	DOT1-like, histone H3 methyltransferase (S. cerevisiae)	Induced	1.34E-02	+1.56
AA007914	Keratin complex 2, basic, gene 6a	Induced	1.34E-02	+8.61
AV054713	Ral GEF with PH domain and SH3 binding motif 2	Induced	1.34E-02	+1.65
AI315174	Syntaxin binding protein 2	Induced	1.34E-02	+1.84
AV083733	RIKEN cDNA 2610528J11 gene	Induced	1.35E-02	+2.28
AV085715	2310015A10	Induced	1.44E-02	+2.29
AV093621	H2A histone family, member X	Induced	1.45E-02	+1.90
AV018519	Ubiquitin-associated protein 2	Induced	1.47E-02	+1.44
BG074318	Small nuclear ribonucleoprotein D2	Induced	1.50E-02	+1.74
AV135009	2810012L14	Induced	1.51E-02	+1.56
BG072453	Epoxide hydrolase 1, microsomal	Induced	1.51E-02	+2.02
BG073755	RIKEN cDNA 9330182L06 gene	Induced	1.52E-02	+2.07
BG063690	RIKEN cDNA 0610009O03 gene	Induced	1.55E-02	+2.35
AV078205	Eukaryotic translation initiation factor 4E binding protein 1	Induced	1.56E-02	+1.26
BG071969	RIKEN cDNA 4732473B16 gene	Induced	1.56E-02	+1.96
BG074852	AarF domain containing kinase 5	Induced	1.57E-02	+2.87
BG074780	Fanconi anemia, complementation group L	Induced	1.58E-02	+2.36
BG072545	CDC28 protein kinase 1b	Induced	1.63E-02	+1.80
BG066812	RIKEN cDNA 1600027N09 gene	Induced	1.65E-02	+2.24
BG063423	CTF18, chromosome transmission fidelity factor 18 homolog (S. cerevisiae)	Induced	1.66E-02	+1.61
AI893759	Vang, van gogh-like 1 (Drosophila)	Induced	1.66E-02	+1.90

AA031147	Annexin A11	Induced	1.67E-02	+1.74
BG063171	RIKEN cDNA 2310014H01 gene	Induced	1.72E-02	+1.83
BG068253	RIKEN cDNA 3110031B13 gene	Induced	1.72E-02	+1.93
AV043702	Actin-like 6A	Induced	1.73E-02	+1.46
AU043350	Budding uninhibited by benzimidazoles 3 homolog (S. cerevisiae)	Induced	1.73E-02	+1.32
BG071162	Fas (TNFRSF6)-associated via death domain	Induced	1.73E-02	+2.54
AA408693	RIKEN cDNA 4633402N23 gene	Induced	1.73E-02	+2.26
BG071659	Tuftelin 1	Induced	1.77E-02	+1.63
BG073181	Cyclin G1	Induced	1.79E-02	+2.13
AV074448	Catechol-O-methyltransferase domain containing 1	Induced	1.80E-02	+1.81
BG072617	Stem-loop binding protein	Induced	1.80E-02	+1.64
BG068799	Cell division cycle associated 5	Induced	1.86E-02	+2.56
AV090113	2310047E02	Induced	1.88E-02	+1.37
AV011145	RIKEN cDNA 1110002E23 gene	Induced	1.88E-02	+1.71
AV037674	Nudix (nucleoside diphosphate linked moiety X)-type motif 14	Induced	1.89E-02	+1.61
AV162171	RIKEN cDNA 3110001D03 gene	Induced	1.90E-02	+1.34
AU045159	Mki67 (FHA domain) interacting nucleolar phosphoprotein	Induced	1.91E-02	+1.90
BG074443	Lectin, galactose binding, soluble 7	Induced	1.92E-02	+1.79
AV060457	RIKEN cDNA 1810063B05 gene	Induced	1.92E-02	+2.90
AA162497	Tripartite motif protein 27	Induced	1.92E-02	+1.74
BG063089	RIKEN cDNA 2510002D24 gene	Induced	1.95E-02	+3.32
AV009795	Thioesterase, adipose associated	Induced	1.96E-02	+1.76
BG070972	RNA, U transporter 1	Induced	2.00E-02	+1.53
BG063678	Thyroid hormone receptor interactor 13	Induced	2.01E-02	+3.12
BG064523	Longevity assurance homolog 5 (S. cerevisiae)	Induced	2.02E-02	+1.81
BG069569	Era (G-protein)-like 1 (E. coli)	Induced	2.03E-02	+1.83
BG067086	Claspin homolog (Xenopus laevis)	Induced	2.04E-02	+2.99
BG073019	Cyclin-dependent kinase 2	Induced	2.04E-02	+1.95
BG074224	RIKEN cDNA 1110002M09 gene	Induced	2.04E-02	+1.29
BG071486	Arginyl-tRNA synthetase-like	Induced	2.05E-02	+1.44
AA031007	Retinoblastoma binding protein 4	Induced	2.08E-02	+1.49
AV006844	RIKEN cDNA 1110001M19 gene	Induced	2.08E-02	+1.82
BG064073	RIKEN cDNA 5730461K03 gene	Induced	2.08E-02	+2.75
BG068207	Catechol-O-methyltransferase domain containing 1	Induced	2.10E-02	+1.91
AV030670	Exosome component 1	Induced	2.10E-02	+1.62
AV090052	Phosphoribosylglycinamide formyltransferase	Induced	2.10E-02	+1.84
BG073819	RIKEN cDNA 1810060D16 gene	Induced	2.10E-02	+1.91

BG064810	Sec61 alpha 1 subunit (S. cerevisiae)	Induced	2.12E-02	+1.94
AV055765	RIKEN cDNA 1300018I05 gene	Induced	2.16E-02	+1.90
BG074816	Leucine zipper protein 5	Induced	2.18E-02	+2.64
AV093509	Polymerase (DNA directed), delta 1, catalytic subunit	Induced	2.18E-02	+1.82
BG070542	RIKEN cDNA 1810006K21 gene	Induced	2.19E-02	+1.35
BG063889	Ring finger protein 41	Induced	2.20E-02	+2.12
AU041357	Ras homolog gene family, member D	Induced	2.23E-02	+1.78
AV067190	2010203M03	Induced	2.26E-02	+1.24
AI854081	DNA segment, Chr 10, Wayne State University 102, expressed	Induced	2.26E-02	+1.78
AV002587	Spindle pole body component 25 homolog (S. cerevisiae)	Induced	2.27E-02	+1.96
BG072882	RIKEN cDNA 1110007C05 gene	Induced	2.28E-02	+1.97
AV113932	2610025P03	Induced	2.29E-02	+2.05
AA007914	Keratin complex 2, basic, gene 6a	Induced	2.31E-02	+6.63
BG064119	RIKEN cDNA 1110056N09 gene	Induced	2.41E-02	+2.95
AW549759	TAF6 RNA polymerase II, TATA box binding protein (TBP)-associated factor	Induced	2.41E-02	+1.61
AV020251	RIKEN cDNA 1810061M12 gene	Induced	2.43E-02	+1.62
AV018540	Stimulated by retinoic acid 13	Induced	2.49E-02	+1.28
AV061212	RIKEN cDNA 9430008C03 gene	Induced	2.52E-02	+1.97
AV094723	RNA binding motif protein 17	Induced	2.52E-02	+1.94
BG062965	Stratifin	Induced	2.56E-02	+1.88
BG063172	Sec61, alpha subunit 2 (S. cerevisiae)	Induced	2.57E-02	+2.01
AV114610	2610035D17	Induced	2.58E-02	+2.13
BG064971	CDNA sequence BC011248	Induced	2.58E-02	+1.89
BG071826	Guanylate kinase 1	Induced	2.66E-02	+1.57
AV036690	Prolactin-like protein F	Induced	2.66E-02	+1.74
BG069766	Neuroblastoma ras oncogene	Induced	2.75E-02	+2.07
AV103928	RIKEN cDNA 2610019I03 gene	Induced	2.76E-02	+2.52
AA049761	CDC28 protein kinase 1b	Induced	2.77E-02	+1.58
BG063109	Integrin beta 4	Induced	2.77E-02	+1.52
AV081325	RIKEN cDNA 4632409L19 gene	Induced	2.78E-02	+1.41
BG067535	Tetratricopeptide repeat domain 4	Induced	2.85E-02	+1.22
BG063980	Coiled-coil-helix-coiled-coil-helix domain containing 2	Induced	2.86E-02	+1.59
BG063134	Procollagen-proline, 2-oxoglutarate 4-dioxygenase (proline 4-hydroxylase), alpha II polypeptide	Induced	2.86E-02	+2.63
BG074649	BG074649	Induced	2.90E-02	+4.24
BG073466	A630082K20Rik	Induced	2.93E-02	+2.41

BG064376	Budding uninhibited by benzimidazoles 3 homolog (S. cerevisiae)	Induced	3.00E-02	+1.58
BG063213	Forkhead box M1	Induced	3.04E-02	+2.01
AV049214	RIKEN cDNA 0610006I08 gene	Induced	3.04E-02	+1.37
AV059331	Ubiquitin-like 4	Induced	3.08E-02	+1.84
AU043663	Importin 4	Induced	3.10E-02	+1.43
BG074217	Expressed sequence AA409316	Induced	3.11E-02	+1.83
BG072792	Arrestin domain containing 1	Induced	3.15E-02	+1.51
AV088360	Williams Beuren syndrome chromosome region 21	Induced	3.15E-02	+1.54
BG070586	NHP2 non-histone chromosome protein 2-like 1 (S. cerevisiae)	Induced	3.16E-02	+1.35
AW558029	Ubiquinol-cytochrome c reductase (6.4kD) subunit	Induced	3.16E-02	+2.28
BG071030	RIKEN cDNA 2610304G08 gene	Induced	3.20E-02	+1.61
AV051025	ATPase type 13A2	Induced	3.22E-02	+1.60
AV037696	LSM4 homolog, U6 small nuclear RNA associated (S. cerevisiae)	Induced	3.22E-02	+1.30
AV140980	RIKEN cDNA 2700062C07 gene	Induced	3.22E-02	+1.37
AA408470	DNA segment, Chr 15, Wayne State University 169, expressed	Induced	3.23E-02	+2.73
AV095264	Cell division cycle associated 3	Induced	3.25E-02	+1.51
AV054939	RNA polymerase 1-3	Induced	3.27E-02	+1.31
BG064541	BG064541	Induced	3.29E-02	+2.30
BG064150	Expressed sequence AW544865	Induced	3.29E-02	+2.03
AV010356	Progestin and adipoQ receptor family member IV	Induced	3.35E-02	+2.13
AV085671	RIKEN cDNA 2410012H22 gene	Induced	3.37E-02	+1.62
AV056014	RIKEN cDNA 1700121I08 gene	Induced	3.39E-02	+1.44
BG069307	RIKEN cDNA 4631424J17 gene	Induced	3.39E-02	+1.42
BG068213	Chromodomain helicase DNA binding protein 1-like	Induced	3.41E-02	+1.25
BG072006	RIKEN cDNA 2310039H08 gene	Induced	3.41E-02	+1.80
BG063289	Dipeptidylpeptidase 4	Induced	3.44E-02	+4.04
AW538759	C0111E01	Induced	3.46E-02	+2.09
AV002974	Glutathione peroxidase 6	Induced	3.46E-02	+1.62
AV095113	COX10 homolog, cytochrome c oxidase assembly protein, heme A: farnesyltransferase (yeast)	Induced	3.53E-02	+1.64
BG063112	Gem (nuclear organelle) associated protein 5	Induced	3.53E-02	+3.23
BG075713	Nucleoporin 50	Induced	3.53E-02	+1.59
AV095025	9	Induced	3.54E-02	+1.43

AV114515	Phytoceramidase, alkaline	Induced	3.56E-02	+2.29
BG070240	RIKEN cDNA 1110025L05 gene	Induced	3.57E-02	+1.84
BG072276	Cysteine-rich protein 1 (intestinal)	Induced	3.59E-02	+1.60
BG067124	Abhydrolase domain containing 4	Induced	3.60E-02	+1.51
BG062975	SRY-box containing gene 13	Induced	3.63E-02	+2.69
BG070894	5',3'-nucleotidase, cytosolic	Induced	3.67E-02	+1.69
AA404089	Kit ligand	Induced	3.67E-02	+1.93
BG068580	PET112-like (yeast)	Induced	3.67E-02	+2.02
AV081283	Ribonucleotide reductase M2	Induced	3.67E-02	+1.75
AW543803	Hypoxia inducible factor 1, alpha subunit	Induced	3.69E-02	+1.37
BG064846	Cell division cycle 2 homolog A (S. pombe)	Induced	3.70E-02	+1.59
BG064095	Uridine-cytidine kinase 2	Induced	3.70E-02	+2.03
AV095249	Surfeit gene 6	Induced	3.72E-02	+1.45
BG067302	Cytochrome c oxidase subunit VIb polypeptide 2	Induced	3.73E-02	+1.81
AA036390	Inhibitor of DNA binding 1	Induced	3.73E-02	+1.84
BG073950	Mitochondrial ribosomal protein L3	Induced	3.73E-02	+1.37
BG073309	Barrier to autointegration factor 1	Induced	3.75E-02	+1.24
AV103331	Gene trap ROSA b-geo 22	Induced	3.76E-02	+1.44
AV050667	1810014K16	Induced	3.80E-02	+1.68
AV061364	Williams Beuren syndrome chromosome region 22	Induced	3.84E-02	+1.58
AV012429	DNA methyltransferase (cytosine-5) 1	Induced	3.85E-02	+1.48
BG062979	COP9 (constitutive photomorphogenic) homolog, subunit 7b (Arabidopsis thaliana)	Induced	3.91E-02	+2.13
BG070492	Synaptoporin	Induced	3.91E-02	+1.68
BI076559	C0181G01	Induced	3.96E-02	+1.60
AV085862	Gene trap locus F3a	Induced	3.98E-02	+2.36
BG068907	UDP-GlcNAc:betaGal beta-1,3-N-acetylglucosaminyltransferase 1	Induced	3.99E-02	+1.98
AV114430	2610034B18	Induced	4.02E-02	+2.11
BG065649	H3033F08	Induced	4.02E-02	+2.42
BG071768	LOC545341	Induced	4.07E-02	+1.85
AW553200	Transcriptional regulator, SIN3A (yeast)	Induced	4.07E-02	+1.54
BG064137	Metadherin	Induced	4.15E-02	+1.78
AV067338	Thrombospondin, type I domain containing 6	Induced	4.19E-02	+2.25
AV025038	Transmembrane protein 43	Induced	4.21E-02	+1.54
AV055189	AV055189	Induced	4.23E-02	+1.88
BG063206	AarF domain containing kinase 2	Induced	4.24E-02	+2.64
BG074564	Guanine monphosphate synthetase	Induced	4.32E-02	+1.85
BG071259	BG071259	Induced	4.37E-02	+3.40
AV065496	2010016E13	Induced	4.42E-02	+1.46

AV171702	RIKEN cDNA 3300001M20 gene	Induced	4.43E-02	+1.41
AV089061	Small proline-rich protein 1B	Induced	4.43E-02	+2.66
BG063452	PWP2 (periodic tryptophan protein) homolog, yeast	Induced	4.45E-02	+4.65
BG063278	RAP2B, member of RAS oncogene family	Induced	4.50E-02	+1.85
BG063959	H3013G12	Induced	4.51E-02	+2.84
BG067439	RIKEN cDNA E430024C06 gene	Induced	4.51E-02	+3.09
BG069879	Expressed sequence 2 embryonic lethal	Induced	4.54E-02	+1.93
AV054283	JTV1 gene	Induced	4.56E-02	+1.44
AV083969	Jun-B oncogene	Induced	4.56E-02	+1.78
BG070608	DEAD (Asp-Glu-Ala-Asp) box polypeptide 18	Induced	4.59E-02	+1.70
AV162365	Insulin-like growth factor 2, binding protein 3	Induced	4.63E-02	+1.64
BG068773	MAM domain containing 1	Induced	4.70E-02	+1.72
AV078377	2210403N22	Induced	4.71E-02	+1.76
AI604914	Transforming growth factor, beta 1	Induced	4.71E-02	+1.68
AV061715	1810074P22	Induced	4.72E-02	+1.45
BG072056	Centromere autoantigen A	Induced	4.72E-02	+1.68
AA137960	IL2-inducible T-cell kinase	Induced	4.73E-02	+2.66
BG063473	BG063473	Induced	4.82E-02	+2.62
AA245472	Homeo box C4	Induced	4.83E-02	+1.73
BG074000	RIKEN cDNA 1200013B22 gene	Induced	4.87E-02	+2.27
BG068648	RIKEN cDNA E430024C06 gene	Induced	4.90E-02	+3.00
AA276890	Rho, GDP dissociation inhibitor (GDI) beta	Induced	4.97E-02	+1.46
BG064731	RIKEN cDNA 2810475A17 gene	Induced	4.97E-02	+1.94
BG073909	Tnf receptor-associated factor 6	Induced	4.97E-02	+2.20
BG063957	Baculoviral IAP repeat-containing 5	Induced	4.97E-02	+1.70
AV073480	Retinol dehydrogenase 12	Induced	4.97E-02	+1.29
AV056245	Calpain, small subunit 1	Induced	4.97E-02	+1.58
AV056304	RIKEN cDNA 1110025L05 gene	Induced	4.97E-02	+1.70
BG063492	RIKEN cDNA 1110064N10 gene	Induced	4.97E-02	+3.52
AV103873	BolA-like 1 (E. coli)	Induced	4.97E-02	+1.58
AV070957	Thrombospondin, type I domain containing 6	Induced	4.97E-02	+2.92
AA408701	C0031G05	Induced	4.97E-02	+1.43
AV048212	Hyaluronidase 2	Induced	4.97E-02	+1.65
BG064822	RIKEN cDNA 1110008B24 gene	Induced	4.97E-02	+1.71
BG073345	RIKEN cDNA 2810028N01 gene	Induced	4.97E-02	+1.75
BG071176	RIKEN cDNA 2900017G11 gene	Induced	4.97E-02	+1.35
AV055700	DEAD (Asp-Glu-Ala-Asp) box polypeptide 55	Induced	4.97E-02	+1.59
BG063227	A'	Induced	4.97E-02	+1.95
AV006659	Translocator of inner mitochondrial membrane 17b	Induced	4.98E-02	+1.65

AV019805	SH2-B PH domain containing signaling mediator 1	Induced	4.98E-02	+1.50
AV134814	Sjogren's syndrome/scleroderma autoantigen 1 homolog (human)	Induced	4.98E-02	+1.78
BG075069	Phosphatidylserine receptor	Induced	4.98E-02	+1.26
AV065609	Mitochondrial ribosomal protein L43	Induced	4.98E-02	+1.16
AI327056	Kinesin family member 22	Induced	4.98E-02	+1.45
AA162498	Protein tyrosine phosphatase, receptor type, E	Induced	4.98E-02	+1.60
BG071001	Uridine-cytidine kinase 2	Induced	4.98E-02	+2.54
BG067973	Spermine oxidase	Induced	4.98E-02	+1.69
BG074410	Splicing factor 3b, subunit 5	Induced	4.98E-02	+1.48
BG063925	Metallothionein 2	Induced	4.98E-02	+2.13
AV067418	Interleukin 18 binding protein	Induced	4.98E-02	+2.03
AV083081	Loricrin	Induced	4.98E-02	+1.54
AV060097	Topoisomerase I binding, arginine/serine-rich	Induced	4.98E-02	+1.59
AV086475	Bcl-2-related ovarian killer protein	Induced	4.98E-02	+1.27
BG064184	RRS1 ribosome biogenesis regulator homolog (S. cerevisiae)	Induced	4.98E-02	+1.68
BG071755	H3102H02	Induced	4.99E-02	+2.26
AV061250	Adenine phosphoribosyl transferase	Induced	4.99E-02	+1.56
AV020091	Ring finger protein 126	Induced	4.99E-02	+1.36
AV037093	Fractured callus expressed transcript 1	Induced	4.99E-02	+1.47
AA144244	V-crk sarcoma virus CT10 oncogene homolog (avian)-like	Induced	4.99E-02	+1.64
AV017255	General transcription factor III C 1	Induced	4.99E-02	+1.60
BG070490	RIKEN cDNA E430025E21 gene	Induced	4.99E-02	+1.67
AV053656	Proteasome (prosome, macropain) subunit, beta type 2	Induced	4.99E-02	+1.24
AV083948	Translocase of inner mitochondrial membrane 9 homolog (yeast)	Induced	4.99E-02	+1.58
BG075765	Defender against cell death 1	Induced	4.99E-02	+1.29
BG073624	H3126B09	Induced	4.99E-02	+2.86
BG074856	Fas-activated serine/threonine kinase	Induced	4.99E-02	+1.54
BG066796	3-phosphoglycerate dehydrogenase	Induced	4.99E-02	+1.37
BG064322	Excision repair cross-complementing rodent repair deficiency, complementation group 1	Induced	4.99E-02	+1.43
AW543765	C0173H02	Induced	4.99E-02	+1.44
AV052028	Myocyte enhancer factor 2B	Induced	4.99E-02	+1.32
AV088404	D-dopachrome tautomerase	Induced	4.99E-02	+1.25
BG063774	Zinc finger, CCHC domain containing 9	Induced	4.99E-02	+2.22
AV043891	Myocyte enhancer factor 2B	Induced	4.99E-02	+1.55

BG064298	UDP-Gal:betaGlcNAc beta 1,4-galactosyltransferase, polypeptide 3	Induced	5.00E-02	+1.90
AV066323	Interferon-stimulated protein	Induced	5.00E-02	+1.55
BG063208	Hepsin	Induced	5.00E-02	+1.95
AV162425	Translocase of inner mitochondrial membrane 23 homolog (yeast)	Induced	5.00E-02	+1.29
BG064513	RIKEN cDNA C130032J12 gene	Induced	5.00E-02	+1.65
BG072903	Zinc finger, RAN-binding domain containing 1	Induced	5.00E-02	+2.26
BG063110	Fibronectin type III domain containing 3a	Induced	5.00E-02	+3.47
AV114591	Ribonuclease H1	Induced	5.00E-02	+2.10
BG068714	H3068E09	Induced	5.00E-02	+1.69
BG064611	MUS81 endonuclease homolog (yeast)	Induced	5.00E-02	+3.11
AV094758	RIKEN cDNA 2410015M20 gene	Induced	5.00E-02	+1.44
AV042076	Solute carrier family 2 (facilitated glucose transporter), member 5	Induced	5.00E-02	+2.20
BG075638	H3149F07	Induced	5.00E-02	+1.40
AV088623	RIKEN cDNA 2010100O12 gene	Induced	5.00E-02	+1.72
AV010817	RIKEN cDNA 1110029E03 gene	Induced	5.00E-02	+1.58
AW544771	C0185G01	Induced	5.00E-02	+1.77
AV007317	Polymerase (DNA directed), lambda	Induced	5.00E-02	+1.88
AV043034	RIKEN cDNA 2900016G09 gene	Induced	5.00E-02	+2.32
BG064249	Chemokine (C-X-C motif) ligand 7	Induced	5.00E-02	+2.99
BG065606	Serine (or cysteine) proteinase inhibitor, clade B, member 6c	Induced	5.00E-02	+2.28
AV042920	DNA segment, Chr 2, ERATO Doi 750, expressed	Induced	5.00E-02	+1.80
AV088345	Small proline rich-like 1	Induced	5.00E-02	+4.19
AA063824	Tripartite motif protein 25	Induced	5.00E-02	+2.71
BG072904	RAD51 homolog (S. cerevisiae)	Induced	5.00E-02	+4.21
BG064942	SCY1-like 2 (S. cerevisiae)	Induced	5.00E-02	+2.38
AV141800	RIKEN cDNA 2700078E11 gene	Induced	5.00E-02	+3.91
BG067980	H3060D07	Induced	5.00E-02	+2.71
AI322376	Notchless homolog 1 (Drosophila)	Induced	5.00E-02	+2.09
BG066288	H3041B10	Induced	5.00E-02	+2.32
BG068906	Cytoplasmic polyadenylation element binding protein 1	Induced	5.00E-02	+1.64
