# Supplementary material for: Distinct Pigmentary and Melanocortin 1 Receptor–Dependent Components of Cutaneous Defense against Ultraviolet Radiation
Source: PLoS Genet. 2007 Jan 12;3(1):e9. doi: 10.1371/journal.pgen.0030009 (PMC1774588; doi:10.1371/journal.pgen.0030009)
Supplement: Supplementary file 2 [file pgen.0030009.st002.pdf]

Table S2. Enriched Gene Ontology (GO) Terms for Downregulated Genes in the Basal Epidermis of P2.5 *Kit*$^{W\text{-}v}$/*Kit*$^{W\text{-}v}$ Mice

GO Organizing Principle	Gene Ontology Term[a]	Fold Enrichment	*p*- Value
Biological Process	melanin biosynthesis	48.69	6.51E-04
Molecular Function	protein transport activity	4.09	2.51E-03
Biological Process	organelle organization and biogenesis	2.8	2.60E-03
Biological Process	carboxylic acid metabolism	3.6	2.94E-03
Molecular Function	ATP binding	2.48	3.53E-03
Molecular Function	adenyl nucleotide binding	2.46	3.72E-03
Cellular Component	actin cytoskeleton	3.76	2.26E-02
Biological Process	small GTPase mediated signal transduction	2.79	3.26E-02
Molecular Function	guanyl nucleotide binding	2.77	3.38E-02
Biological Process	electron transport	2.31	4.44E-02

[a]Enriched GO terms, that were significantly enriched ($p \leq 0.05$) by ≥2-fold, relative to all of the assayed genes on the 35K array (35328 genes), were obtained by Over-Representation Analysis using EASE software (Hosack *et al* ., 2003).
