# Supplementary material for: Distinct Pigmentary and Melanocortin 1 Receptor–Dependent Components of Cutaneous Defense against Ultraviolet Radiation
Source: PLoS Genet. 2007 Jan 12;3(1):e9. doi: 10.1371/journal.pgen.0030009 (PMC1774588; doi:10.1371/journal.pgen.0030009)
Supplement: Supplementary file 3 [file pgen.0030009.st003.pdf]

**Table S3**. Enriched Gene Ontology (GO) Terms for Upregulated Genes in the Basal Epidermis of P2.5 *Kit*$^{W\text{-}v}$/*Kit*$^{W\text{-}v}$ Mice			
**GO Organizing Principle**	**Gene Ontology Term**[a]	**Fold Enrichment**	***p*-Value**
Biological Process	embryogenesis and morphogenesis	8.28	5.47E-03
Molecular Function	phosphoric monoester hydrolase activity	4.87	8.88E-03
Molecular Function	catalytic activity	2.15	9.43E-03
Biological Process	morphogenesis	2.19	1.46E-02
Biological Process	RNA processing	2.84	1.79E-02
Molecular Function	signal transducer activity	2.31	2.10E-02
Biological Process	development	2.28	2.32E-02
Molecular Function	cell adhesion molecule activity	3.61	2.42E-02
Cellular Component	extracellular space	2.17	2.59E-02
Molecular Function	enzyme regulator activity	2.52	4.55E-02

[a]Enriched GO terms, that were significantly enriched ($p \leq 0.05$) by ≥2-fold, relative to all of the assayed genes on the 35K array (35328 genes), were obtained by Over-Representation Analysis using EASE software (Hosack *et al*., 2003).
