# Supplementary material for: Distinct Pigmentary and Melanocortin 1 Receptor–Dependent Components of Cutaneous Defense against Ultraviolet Radiation
Source: PLoS Genet. 2007 Jan 12;3(1):e9. doi: 10.1371/journal.pgen.0030009 (PMC1774588; doi:10.1371/journal.pgen.0030009)
Supplement: Supplementary file 4 [file pgen.0030009.st004.pdf]

**Table S4.** Mc1r-Dependent UVB-Responsive Genes in the Basal Epidermis of P2.5 Mice		
**Accession ID**	**Gene Name**	**UVB Response**
BG074162	BG074162	Induced in *Mc1r* ^{e} /*Mc1r* ^{e}
BG072062	Cysteine and histidine-rich domain (CHORD)-containing, zinc-binding protein 1	Induced in *Mc1r* ^{e} /*Mc1r* ^{e}
BG071813	Topoisomerase (DNA) III alpha	Induced in *Mc1r* ^{e} /*Mc1r* ^{e}
BG071000	IMP3, U3 small nucleolar ribonucleoprotein, homolog (yeast)	Induced in *Mc1r* ^{e} /*Mc1r* ^{e}
AV056089	Asparagine-linked glycosylation 5 homolog (yeast, dolichyl-phosphate beta-glucosyltransferase)	Induced in *Mc1r* ^{e} /*Mc1r* ^{e}
AI836964	Blocked early in transport 1 homolog (S. cerevisiae)-like	Induced in *Mc1r* ^{e} /*Mc1r* ^{e}
BG073903	Expressed sequence C78541	Induced in *Mc1r* ^{e} /*Mc1r* ^{e}
AV014799	Regulatory solute carrier protein, family 1, member 1	Induced in *Mc1r* ^{e} /*Mc1r* ^{e}
AU045309	RIKEN cDNA 2700038C09 gene	Induced in *Mc1r* ^{e} /*Mc1r* ^{e}
BG075933	MAP kinase-activated protein kinase 2	Induced in *Mc1r* ^{e} /*Mc1r* ^{e}
BG068911	Inositol polyphosphate multikinase	Induced in *Mc1r* ^{e} /*Mc1r* ^{e}
C76049	RIKEN cDNA 2310079N02 gene	Induced in *Mc1r* ^{e} /*Mc1r* ^{e}
AA137828	Cell division cycle 2-like 1	Induced in *Mc1r* ^{e} /*Mc1r* ^{e}
BG074549	RAB35, member RAS oncogene family	Induced in *Mc1r* ^{e} /*Mc1r* ^{e}
AI837774	RIKEN cDNA 0610007C21 gene	Induced in *Mc1r* ^{e} /*Mc1r* ^{e}
BG073311	F-box protein 10	Induced in *Mc1r* ^{e} /*Mc1r* ^{e}
BG069588	MAP/microtubule affinity-regulating kinase 3	Induced in *Mc1r* ^{e} /*Mc1r* ^{e}
AU042876	Cyclin-dependent kinase 5	Induced in *Mc1r* ^{e} /*Mc1r* ^{e}
BG064747	Glutamic pyruvic transaminase 1, soluble	Induced in *Mc1r* ^{e} /*Mc1r* ^{e}
BG073940	TGFB-induced factor 2	Induced in *Mc1r* ^{e} /*Mc1r* ^{e}
AW553290	Protein phosphatase 3, catalytic subunit, alpha isoform	Induced in *Mc1r* ^{e} /*Mc1r* ^{e}
AV071031	RIKEN cDNA 1810009O10 gene	Induced in *Mc1r* ^{e} /*Mc1r* ^{e}
BG069869	Cleavage and polyadenylation specificity factor 3	Induced in *Mc1r* ^{e} /*Mc1r* ^{e}
AV013568	Phosphofurin acidic cluster sorting protein 2	Induced in *Mc1r* ^{e} /*Mc1r* ^{e}
AV084607	RIKEN cDNA 2310007O11 gene	Induced in *Mc1r* ^{e} /*Mc1r* ^{e}
AV033582	Protein phosphatase 1, regulatory (inhibitor) subunit 11	Induced in *Mc1r* ^{e} /*Mc1r* ^{e}
BG066456	ATPase type 13A3	Induced in *Mc1r* ^{e} /*Mc1r* ^{e}
AI840560	AI840560	*Mc1r* ^{e} /*Mc1r* ^{e}
BG071796	Cadherin 22	*Mc1r* ^{e} /*Mc1r* ^{e}
BG070959	RalA binding protein 1	*Mc1r* ^{e} /*Mc1r* ^{e}
BG064900	Stearoyl-Coenzyme A desaturase 2	*Mc1r* ^{e} /*Mc1r* ^{e}

AV039046	RIKEN cDNA 2810439K08 gene	$Mc1r^{e}/Mc1r^{e}$
BG064919	Interleukin enhancer binding factor 3	$Mc1r^{e}/Mc1r^{e}$
AV093668	Trinucleotide repeat containing 5	$Mc1r^{e}/Mc1r^{e}$
BG069575	RIKEN cDNA 4933433P14 gene	$Mc1r^{e}/Mc1r^{e}$
AV030759	Nucleobindin 1	$Mc1r^{e}/Mc1r^{e}$
AA060259	Fibroblast growth factor receptor 1	$Mc1r^{e}/Mc1r^{e}$
BG074722	Lethal giant larvae homolog (Llglh), mRNA	$Mc1r^{e}/Mc1r^{e}$
AW550881	Ngg1 interacting factor 3-like 1 (S. pombe)	$Mc1r^{e}/Mc1r^{e}$
AV085145	Aconitase 2, mitochondrial	$Mc1r^{e}/Mc1r^{e}$
AU017897	Heterogeneous nuclear ribonucleoprotein U-like 2	$Mc1r^{e}/Mc1r^{e}$
AA726630	Matrix metallopeptidase 15	$Mc1r^{e}/Mc1r^{e}$
BG074412	Eukaryotic translation initiation factor 4E	$Mc1r^{e}/Mc1r^{e}$
AV055176	Block of proliferation 1	$Mc1r^{e}/Mc1r^{e}$
BG064547	Phosphoglycerate mutase family member 5	$Mc1r^{e}/Mc1r^{e}$
AW538502	AW538502	Induced in +/+
BG064299	RIKEN cDNA 2610027L16 gene	Induced in +/+
BG073159	Zinc finger, DHHC domain containing 16	Induced in +/+
BG063675	Coactivator-associated arginine methyltransferase 1	Induced in +/+
AV094469	BH3 interacting domain death agonist	Induced in +/+
BG064332	Solute carrier family 38, member 4	Induced in +/+
BG063173	Actinin, alpha 1	Induced in +/+
BG069551	RIKEN cDNA 2810406C15 gene	Induced in +/+
BG069420	RIKEN cDNA 5430405G24 gene	Induced in +/+
AV093629	Prohibitin	Induced in +/+
AA119076	RNA binding motif protein 14	Induced in +/+
AV141788	Far upstream element (FUSE) binding protein 1	Induced in +/+
AW544025	CNDP dipeptidase 2 (metallopeptidase M20 family)	Induced in +/+
BG063386	Actin related protein 2/3 complex, subunit 1B	Induced in +/+
AI837011	Cytochrome c oxidase, subunit VIIc	Induced in +/+
BI076763	Transmembrane emp24-like trafficking protein 10 (yeast)	Induced in +/+
AV058611	TATA box binding protein-like 1	Induced in +/+
AV036000	AV036000	Repressed in +/+
AW552656	AW552656	Repressed in +/+
AW555131	AW555131	Repressed in +/+
BG073488	BG073488	Repressed in +/+
BG073778	BG073778	Repressed in +/+
AW549112	AW549112	Repressed in +/+
AW545825	AW545825	Repressed in +/+
AV083093	S100 calcium binding protein A10 (calpactin)	Repressed in +/+

AV094800	Stromal cell derived factor receptor 1	Repressed in +/+
AV013645	Mature T-cell proliferation 1	Repressed in +/+
BE629868	Brain protein 44	Repressed in +/+
BG072628	Sex comb on midleg homolog 1	Repressed in +/+
BG063228	Kelch domain containing 4	Repressed in +/+
BG075418	FK506 binding protein 9	Repressed in +/+
AV077941	RIKEN cDNA 1700021F05 gene	Repressed in +/+
AV103504	ATPase inhibitory factor 1	Repressed in +/+
BG064033	Signal sequence receptor, gamma	Repressed in +/+
AV009257	G protein-coupled receptor 155	Repressed in +/+
AA002910	FBJ osteosarcoma oncogene	Repressed in +/+
AA002910	FBJ osteosarcoma oncogene	Repressed in +/+
BG075124	RAB12, member RAS oncogene family	Repressed in +/+
BG075293	Muscleblind-like 1 (Drosophila)	Repressed in +/+
AW553641	DCUN1D1 DCN1, defective in cullin neddylation 1, domain containing 1 (S. cerevisiae)	Repressed in +/+
AV113584	Histone 1, H2bc	Repressed in +/+
AV036603	Placental specific protein 1	Repressed in +/+
BG072724	Transmembrane emp24 domain containing 3	Repressed in +/+
AV109006	RIKEN cDNA 2510049I19 gene	Repressed in +/+
AV005393	Hemoglobin, beta adult major chain	Repressed in +/+
AV106768	Hemoglobin, beta adult major chain	Repressed in +/+
AV095575	Heat shock protein 8	Repressed in +/+
AV065227	RIKEN cDNA 1700023B02 gene	Repressed in +/+
AV094771	Complement component 1, q subcomponent binding protein	Repressed in +/+
BG063847	Ribosomal protein L8	Repressed in +/+
AV037629	Cathepsin J	Repressed in +/+
BG072802	Ribosomal protein S16	Repressed in +/+
AV107423	Heat shock 70kD protein 5 (glucose-regulated protein)	Repressed in +/+
BG068736	Pyruvate dehydrogenase E1 alpha 1	Repressed in +/+
AW549980	Ubiquitin-conjugating enzyme E2N	Repressed in +/+
AV032302	RAB7, member RAS oncogene family	Repressed in +/+
AU041088	PDZ and LIM domain 1 (elfin)	Repressed in +/+
BG072526	Ribosomal protein L13	Repressed in +/+
BG064712	Growth factor receptor bound protein 2	Repressed in +/+
AV089802	Adenosine deaminase	Repressed in +/+
AV048197	Tripartite motif containing 68	Repressed in +/+
BG070758	Suppressor of Ty 4 homolog 1 (S. cerevisiae)	Repressed in +/+
AW554069	AW554069	Both[a]
AV061568	Chromatin accessibility complex 1	Both[a]

BG074487	Serine (or cysteine) peptidase inhibitor, clade B, member 6a	Both[a]
AV106770	RIKEN cDNA 2510027J23 gene	Both[a]
AV106041	Hemoglobin, beta adult major chain	Both[a]
AV107375	Hemoglobin, beta adult minor chain	Both[a]
BG069480	DNA segment, Chr 19, ERATO Doi 737, expressed	Both[a]
AA049547	RAS-related C3 botulinum substrate 1	Both[a]
AV066077	Defensin-related cryptdin 20	Both[a]
BG073305	Poliovirus receptor-related 1	Both[a]
BG064610	Coiled-coil-helix-coiled-coil-helix domain containing 4	Both[a]
BG063630	H2A histone family, member Z	Both[a]
AV003504	RIKEN cDNA 0610030E20 gene	Both[a]

[a]UVB-responsive in both genotypes
