# Supplementary material for: Distinct Pigmentary and Melanocortin 1 Receptor–Dependent Components of Cutaneous Defense against Ultraviolet Radiation
Source: PLoS Genet. 2007 Jan 12;3(1):e9. doi: 10.1371/journal.pgen.0030009 (PMC1774588; doi:10.1371/journal.pgen.0030009)
Supplement: Supplementary file 5 [file pgen.0030009.st005.pdf]

**Table S5.** Melanocyte-Dependent UVB-Responsive Genes in the Basal Epidermis of P2.5 Mice		
**Accession ID**	**Gene Name**	**UVB Response**
AV093152	AV093152	Induced in *Kit* $^{W-v}$/*Kit* $^{W-v}$
BG073251	RIKEN cDNA 2610005L07 gene	Induced in *Kit* $^{W-v}$/*Kit* $^{W-v}$
BG065598	RIKEN cDNA 1110061O04 gene	Induced in *Kit* $^{W-v}$/*Kit* $^{W-v}$
AU045309	RIKEN cDNA 2700038C09 gene	Induced in *Kit* $^{W-v}$/*Kit* $^{W-v}$
AV065229	Acyl-Coenzyme A binding domain containing 4	Induced in *Kit* $^{W-v}$/*Kit* $^{W-v}$
BG073757	RIKEN cDNA F730014I05 gene	Induced in *Kit* $^{W-v}$/*Kit* $^{W-v}$
BG072921	H2A histone family, member Y2	Induced in *Kit* $^{W-v}$/*Kit* $^{W-v}$
AW552541	Cullin-associated and neddylation-dissociated 2 (putative)	Induced in *Kit* $^{W-v}$/*Kit* $^{W-v}$
AV078237	Histone 2, H2aa2	Induced in *Kit* $^{W-v}$/*Kit* $^{W-v}$
AV055389	Membrane-associated ring finger (C3HC4) 5	Induced in *Kit* $^{W-v}$/*Kit* $^{W-v}$
BG069368	Regulatory factor X, 1 (influences HLA class II expression)	Induced in *Kit* $^{W-v}$/*Kit* $^{W-v}$
AV008890	DDHD domain containing 1	Repressed in *Kit* $^{W-v}$/*Kit* $^{W-v}$
AV085188	RIKEN cDNA 2310010I16 gene	Repressed in *Kit* $^{W-v}$/*Kit* $^{W-v}$
AV036826	Protein kinase C substrate 80K-H	Repressed in *Kit* $^{W-v}$/*Kit* $^{W-v}$
AV094849	RIKEN cDNA 2410003A14 gene	Repressed in *Kit* $^{W-v}$/*Kit* $^{W-v}$
AV066552	Tubulointerstitial nephritis antigen	Repressed in *Kit* $^{W-v}$/*Kit* $^{W-v}$
BG070076	Programmed cell death 8	Repressed in *Kit* $^{W-v}$/*Kit* $^{W-v}$
AV014863	Ribulose-5-phosphate-3-epimerase	Repressed in *Kit* $^{W-v}$/*Kit* $^{W-v}$
AV004327	RIKEN cDNA 0610041E09 gene	Repressed in *Kit* $^{W-v}$/*Kit* $^{W-v}$
AV059999	RER1 retention in endoplasmic reticulum 1 homolog (S. cerevisiae)	Repressed in *Kit* $^{W-v}$/*Kit* $^{W-v}$
AI840761	Phosphatase and tensin homolog	Repressed in *Kit* $^{W-v}$/*Kit* $^{W-v}$
AV036342	Transmembrane protein 37	Repressed in *Kit* $^{W-v}$/*Kit* $^{W-v}$
AV008158	Ubiquitin associated domain containing 1	Repressed in *Kit* $^{W-v}$/*Kit* $^{W-v}$
BG070007	ZNUAK family, SNF1-like kinase, 1	Repressed in *Kit* $^{W-v}$/*Kit* $^{W-v}$
AV015828	Xenotropic and polytropic murine retrovirus receptor	Repressed in *Kit* $^{W-v}$/*Kit* $^{W-v}$
AV024030	RIKEN cDNA 9130229H14 gene	Repressed in *Kit* $^{W-v}$/*Kit* $^{W-v}$
BG067057	Mannosidase, alpha, class 1A, member 2	Repressed in *Kit* $^{W-v}$/*Kit* $^{W-v}$
AV083372	Polymerase (RNA) II (DNA directed) polypeptide D	Repressed in *Kit* $^{W-v}$/*Kit* $^{W-v}$
AV083947	G protein-coupled receptor 175	Repressed in *Kit* $^{W-v}$/*Kit* $^{W-v}$
AV042937	Tubulin, alpha 3	Repressed in *Kit* $^{W-v}$/*Kit* $^{W-v}$

AV113506	Zinc finger protein 263	Repressed in *Kit* $^{W-v}$ /*Kit* $^{W-v}$
BG069611	NudC domain containing 1	Repressed in *Kit* $^{W-v}$ /*Kit* $^{W-v}$
AV056619	Hepatoma-derived growth factor	Repressed in *Kit* $^{W-v}$ /*Kit* $^{W-v}$
AV020098	Leucine rich repeat containing 42	Repressed in *Kit* $^{W-v}$ /*Kit* $^{W-v}$
AV066015	C1GALT1-specific chaperone 1	Repressed in *Kit* $^{W-v}$ /*Kit* $^{W-v}$
AV095119	RAS-homolog enriched in brain	Repressed in *Kit* $^{W-v}$ /*Kit* $^{W-v}$
AA063914	Tubulin, alpha 8	Repressed in *Kit* $^{W-v}$ /*Kit* $^{W-v}$
BG072534	Heterogeneous nuclear ribonucleoprotein A1	Repressed in *Kit* $^{W-v}$ /*Kit* $^{W-v}$
AV095153	ATP synthase, H+ transporting, mitochondrial F0 complex, subunit c (subunit 9), isoform 2	Repressed in *Kit* $^{W-v}$ /*Kit* $^{W-v}$
BG065224	Ubiquitin-conjugating enzyme E2Q (putative)	Repressed in *Kit* $^{W-v}$ /*Kit* $^{W-v}$
AV085561	Ubiquitin-conjugating enzyme E2Q (putative)	Repressed in *Kit* $^{W-v}$ /*Kit* $^{W-v}$
AV007192	MLIP1 mRNA for lipoic acid synthase	Induced in +/+
AV094993	Nucleoporin 37	Induced in +/+
BG065169	DEAD (Asp-Glu-Ala-Asp) box polypeptide 39	Induced in +/+
AV081359	Intergral membrane protein 1	Induced in +/+
BG074889	RIKEN cDNA 1110038B12 gene	Induced in +/+
C85855	ASF1 anti-silencing function 1 homolog B (S. cerevisiae)	Induced in +/+
BG075903	NADH dehydrogenase (ubiquinone) 1, subcomplex unknown, 1	Induced in +/+
AV009166	AV009166	Repressed in +/+
BG064785	Cytochrome b	Repressed in +/+
AW555035	AW555035	Repressed in +/+
BG064134	BG064134	Repressed in +/+
BG065042	BG065042	Repressed in +/+
BG064806	BG064806	Repressed in +/+
BG075440	BG075440	Repressed in +/+
AW543815	AW543815	Repressed in +/+
BG074285	Eukaryotic translation initiation factor 1	Repressed in +/+
BG072379	Fc receptor, IgG, high affinity I	Repressed in +/+
BG067009	RIKEN cDNA 2500003M10 gene	Repressed in +/+
AV082658	Interleukin 1 alpha	Repressed in +/+
AV113681	Nuclear receptor coactivator 6 interacting protein	Repressed in +/+
BG066863	DNA segment, Chr 8, ERATO Doi 325, expressed	Repressed in +/+
AI838635	Protein tyrosine phosphatase 4a2	Repressed in +/+
AV107220	Hemoglobin alpha, adult chain 1	Repressed in +/+
AV007503	AHNAK nucleoprotein (desmoyokin)	Repressed in +/+

AV059853	Cullin 4A	Repressed in +/+
AA050018	Heat shock 27kDa protein 8	Repressed in +/+
AI327046	Selenoprotein P, plasma, 1	Repressed in +/+
BG064042	3-monooxygenase/tryptophan 5-monooxygenase activation protein, gamma polypeptide	Repressed in +/+
BG064043	3-monooxygenase/tryptophan 5-monooxygenase activation protein, gamma polypeptide	Repressed in +/+
AV011032	Procollagen, type VI, alpha 1	Repressed in +/+
AW552084	SH3-domain GRB2-like B1 (endophilin)	Repressed in +/+
BG074106	Anaphase promoting complex subunit 4	Repressed in +/+
BG071389	Glutamate-rich WD repeat containing 1 (Grwd1)	Repressed in +/+
BG075168	Expressed sequence C78339	Repressed in +/+
AV052763	Peroxiredoxin 5	Repressed in +/+
AW552247	Non-POU-domain-containing, octamer binding protein	Repressed in +/+
BG069956	V-crk sarcoma virus CT10 oncogene homolog (avian)	Repressed in +/+
AA146115	Serine/threonine kinase 25 (yeast)	Repressed in +/+
AA142750	Adenylate cyclase 7	Repressed in +/+
AV086468	TBC1 domain family, member 20	Repressed in +/+
AV048883	Asparaginyl-tRNA synthetase	Repressed in +/+
AV083160	CD24a antigen	Repressed in +/+
AV093796	Surfeit gene 4	Repressed in +/+
BG074976	Ribosomal protein L8	Repressed in +/+
AV162343	Lysosomal-associated protein transmembrane 4A	Repressed in +/+
BG068617	Lysosomal-associated protein transmembrane 4A	Repressed in +/+
AV036969	Prothymosin alpha	Repressed in +/+
BG074803	Heat shock protein 8	Repressed in +/+
BG068863	Ecotropic viral integration site 5	Repressed in +/+
BG064615	RIKEN cDNA 2310011J03 gene	Repressed in +/+
AV104427	Poly A binding protein, cytoplasmic 1	Repressed in +/+
AI840050	Short coiled-coil protein	Repressed in +/+
AV036624	Glyoxalase 1	Repressed in +/+
BG064712	Growth factor receptor bound protein 2 (Grb2)	Repressed in +/+
AV089802	Adenosine deaminase	Repressed in +/+
BG065782	Cysteine-rich hydrophobic domain 1	Repressed in +/+
AV030593	Elongation of very long chain fatty acids (FEN1/Elo2, SUR4/Elo3, yeast)-like 4	Repressed in +/+

BG072989	BG072989	Both[a]
AW546889	AW546889	Both[a]
AW554432	AW554432	Both[a]
BG071729	NACHT, leucine rich repeat and PYD containing 5	Both[a]
BG075252	EGL nine homolog 1 (C. elegans)	Both[a]
AV162211	ERGIC and golgi 3	Both[a]
AU041154	RIKEN cDNA 1110002N22 gene	Both[a]
BG074377	Hexokinase 1	Both[a]
AW553730	RIKEN cDNA 1110061O04 gene	Both[a]
AA038537	Kallikrein 5	Both[a]
AV083851	Membrane-bound transcription factor peptidase, site 1	Both[a]
AW555640	Inner membrane protein, mitochondrial	Both[a]
BG063087	RIKEN cDNA 2610204K14 gene	Both[a]
BG071467	Zinc finger protein 277	Both[a]
BG071639	RIKEN cDNA 1810031K17 gene	Both[a]
BG071637	RIKEN cDNA 1110049F12 gene	Both[a]
BG074607	Adiponectin receptor 2	Both[a]
AV055667	DAZ associated protein 2	Both[a]
AA050220	RIKEN cDNA 3100004P22 gene	Both[a]
AW551596	Ankyrin repeat domain 11	Both[a]
AV105116	Prkr interacting protein 1 (IL11 inducible)	Both[a]
BG075394	Nuclear receptor binding protein 2	Both[a]
BG075256	Atrophin 1	Both[a]
BG072540	Cyclin D2	Both[a]
BG071439	EIG 180 mRNA for ethanol induced gene product	Both[a]
AA064119	Thymus cell antigen 1, theta	Both[a]
AW556296	Cancer susceptibility candidate 3	Both[a]
BG064004	Zinc finger protein 219	Both[a]
AI327441	Cytochrome P450, family 2, subfamily j, polypeptide 6	Both[a]
AV036341	AV036341	Both[a]
AW538230	AW538230	Both[a]
BG075783	RIKEN cDNA 4930548G07 gene	Both[a]

BG069993	Glutamic pyruvate transaminase (alanine aminotransferase) 2	Both[a]
BG073159	Zinc finger, DHHC domain containing 16	Both[a]
AV088624	Mitochondrial ribosomal protein L16	Both[a]
AV088629	Ring finger protein 14	Both[a]
AA170690	Peroxiredoxin 4	Both[a]
BG066866	RIKEN cDNA 1700110N18 gene	Both[a]
AI840620	Dynactin 4	Both[a]
BG064498	Isocitrate dehydrogenase 3 (NAD+) alpha	Both[a]
AV060291	Mitochondrial ribosomal protein S18A	Both[a]
AV003251	Ethylmalonic encephalopathy 1	Both[a]
BG063903	DEAD (Asp-Glu-Ala-Asp) box polypeptide 17	Both[a]
AV057617	Cyclin-dependent kinase 5	Both[a]
BG067192	Calcium and integrin binding 1 (calmyrin)	Both[a]
AV085276	Exostoses (multiple) 1	Both[a]
BG069832	Inositol polyphosphate-5-phosphatase E	Both[a]
AA124630	Platelet/endothelial cell adhesion molecule 1, transcript variant 2	Both[a]
AV004563	Defensin beta 29	Both[a]

[a]UVB-responsive in both genotypes
