# Supplementary material for: Distinct Pigmentary and Melanocortin 1 Receptor–Dependent Components of Cutaneous Defense against Ultraviolet Radiation
Source: PLoS Genet. 2007 Jan 12;3(1):e9. doi: 10.1371/journal.pgen.0030009 (PMC1774588; doi:10.1371/journal.pgen.0030009)
Supplement: Supplementary file 6 [file pgen.0030009.st006.pdf]

Table S6. Summary of Large-Scale Gene Expression Studies that have Identified UV-Responsive Genes

Species	Tissue/Cell line	UV type	Experimental Design	Platform	Array size	# UV genes	Reference
Human	Keratinocyte cell line (HaCaT)	UVB	Single dose and time point	ATLAS cDNA Expression Array (Clontech)	588	7	Catani et al. (2001)
Human	Primary keratinocyte culture	UVB 312nm	Dose response and time point	I.M.A.G.E. cDNA filters (custom)	≈50000	51	Becker et al. (2001)
Human	Primary keratinocyte culture	UVB 302nm	Single dose and timecourse	ATLAS cDNA Expression Array (Clontech)	588	64	Murakami et al. (2001)
Human	Foreskin keratinocyte culture	UVB	Single dose and timecourse	Hu6800 (Affymetrix)	≈6800	198	Li et al. (2001)
Human	Primary melanocyte culture	UVB 312nm	Single dose and time point	Spotted cDNA microarray (custom)	≈9000	198	Valéry et al. (2001)
Human	Primary melanocyte culture	UVA 365nm	Single dose and time point	ATLAS cDNA Expression Array (Clontech)	588	11	Jean et al. (2001)
Human	Foreskin keratinocyte culture	UVB 313nm	Single dose and time point	HuGeneFL (Affymetrix)	≈6800	187	Takao et al. (2002)
Human	Foreskin keratinocyte culture	UVB	Dose response and timecourse	HuGeneFL (Affymetrix)	≈6800	539	Sesto et al. (2002)
Human	HeLa cells	UVC 254nm	Single dose and timecourse	Millenium nylon membranes (Custom)	886	16	Guo et al. (2002)
Human	Pterygium epithelial cell cultures	UVB	Single dose and timecourse	ATLAS cDNA Expression Array (Clontech)	268	49	Nolan et al. (2003)
Human	Foreskin keratinocyte culture and SSC line (SCC12B2)	UVB 312nm	Single dose and timecourse	HGU95Av2 (Affymetrix)	12651	817	Dazard et al. (2003)
Human	HeLa cells and XP fibroblast cell line (XP4PA-SV)	UVC 254nm	Single dose and timecourse	Millenium nylon membranes (Custom)	886	11	Chang et al. (2003)
Human	Skin fibroblast cell line (WS1)	UVC 254nm	Dose response and timecourse	HGU95Av2 (Affymetrix)	12651	460	Gentile et al. (2003)
Fly	Retina	UVC 254nm	Single dose and time point	*Drosophila* GeneChip (Affymetrix)	≈13500	111	Jassim et al. (2003)
Human	Skin fibroblast cell line (TIG-112)	UVA 365nm	Single dose and time point	Intelligence Human Chip 1K (Takara)	≈1000	3	Abe et al. (2003)
Human	Keratinocyte cell line (HaCaT)	UVA	Dose response and timecourse	ATLAS Human Cancer 1.2 (Clontech)	≈1200	77	He et al. (2004)
Human	Colorectal cancer cell line (HT29-tsp53)	UVC 254nm	Single dose and time point	HGU95Av2 (Affymetrix)	12651	116	McKay et al. (2004)
Human	Foreskin keratinocyte culture	UVB 313nm	Single dose and time point	Spotted cDNA microarray (custom)	1718	84	Howell et al. (2004)
Human	Breast cancer cell line (MCF-7)	UVR	Single dose and timecourse	Spotted cDNA microarray (custom)	7684	795	Koch-Paiz et al. (2004)
Human	Forearm epidermal sheets	UVB 313nm	Single dose and timecourse	HGU95Av2 (Affymetrix)	12651	802	Enk et al. (2004)
Human	Lens epithelial cell line (HLE-B3)	UVA 365nm	Single dose and time point	HGU95Av2 (Affymetrix)	12651	78	Andley et al. (2004)
Human	Lymphoblastoid cell lines	UVC	Single dose and time point	HGU95Av2 (Affymetrix)	12651	3143	Rieger et al. (2004)
Human	Adult keratinocyte culture	UVB	Single dose and timecourse	Spotted oligoarray (custom)	761	34	Pisarchik et al. (2004)
Human	Keratinocyte cell line (HaCaT)	UVB 310nm	Single dose and timecourse	Spotted cDNA microarray (custom)	840	192	Lee et al. (2005)
Rat	Cardiac myocyte and fibroblast cultures	UVC	Single dose and time point	RG-U34A (Affymetrix)	≈7000	3220	Boerma et al. (2005)
Human	Keratinocyte cell line (HaCaT)	UVA	Single dose and time point	HGU95Av2 (Affymetrix)	12651	568	Wertz et al. (2005)
Human	XP fibroblast cell lines	UVC	Single dose and time point	HGU95Av2 (Affymetrix)	12651	2152	da Costa et al. (2005)
Human	Forearm epidermal sheets	UVB 313nm	Single dose and timecourse	Human Genome Focus Array (Affymetrix)	≈8500	619	Enk et al. (2006)

SSC = squamous cell carcinoma; XP = Xeroderma pigmentosum; UVR = UV radiation, encompassing all UV wavelengths (254-400 nm)
