# Supplementary material for: Distinct Pigmentary and Melanocortin 1 Receptor–Dependent Components of Cutaneous Defense against Ultraviolet Radiation
Source: PLoS Genet. 2007 Jan 12;3(1):e9. doi: 10.1371/journal.pgen.0030009 (PMC1774588; doi:10.1371/journal.pgen.0030009)
Supplement: Supplementary file 7 [file pgen.0030009.st007.pdf]

**Table S7.** Non-Overlapping UVB-Responsive Genes in the +/+ Basal Epidermis of P2.5		
**Accession ID**	**Gene Name**	**UVB Response**
AV000446	0610008C16	Repressed
AV014618	1110055I03	Repressed
AV036152	1600017F23	Repressed
AV039876	1700009P10	Repressed
1810038O03	1810038O03	Repressed
AV061704	1810074P07	Repressed
AV065884	2010104L04	Repressed
AV071317	2010323D05	Repressed
AV072536	2200004B21	Repressed
2200004N05	2200004N05	Repressed
2210003C24	2210003C24	Repressed
BB565248	2300001F12	Repressed
2300001N02	2300001N02	Repressed
AV083633	2310003J02	Repressed
AV083850	2310004F20	Repressed
AV085190	2310010I19	Repressed
AW558546	2310016C16Rik	Repressed
2310020E16	2310020E16	Repressed
2310041F23	2310041F23	Repressed
2310050O13	2310050O13	Repressed
AV140966	2810407D09	Repressed
AV171630	3300001I07	Repressed
AK034279	3-hydroxyisobutyrate dehydrogenase	Repressed
AV071929	3-oxoacid CoA transferase 1	Repressed
BG065344	A kinase (PRKA) anchor protein 8	Repressed
AV095037	Acidic ribosomal phosphoprotein P0	Repressed
BF101128	Acidic ribosomal phosphoprotein P0	Repressed
AV001502	Aconitase 1	Repressed
AK034942	Acyl-CoA synthetase long-chain family member 5	Repressed
AV135760	Acyl-Coenzyme A dehydrogenase family, member 9	Repressed
BG073880	Adiponectin receptor 1	Repressed
AV084196	Adipose differentiation related protein	Repressed
BG064935	ADP-ribosylarginine hydrolase	Repressed
AV042402	ADP-ribosylation factor 1	Repressed
AK075941	ADP-ribosylation factor-like 10C	Repressed

AV141401	ADP-ribosylation factor-like 5	Repressed
AV015499	ADP-ribosylation factor-like 6 interacting protein 2	Repressed
AI840764	AI840764	Repressed
AV061968	Alcohol dehydrogenase 6A (class V)	Repressed
AV043327	Aldolase 1, A isoform, pseudogene 1	Repressed
NM_009932	Alpha-2 type IV collagen	Repressed
AI838638	Anaplastic lymphoma kinase	Repressed
BG074892	Annexin A6	Repressed
AV084024	Armadillo repeat gene deleted in velo-cardio-facial syndrome	Repressed
AV095041	Arrestin domain containing 4	Repressed
BG067898	ATP synthase F0 subunit 6	Repressed
AA119394	ATP synthase, H+ transporting, mitochondrial F0 complex, subunit b, isoform 1	Repressed
AV031502	ATPase, H+ transporting, V1 subunit A, isoform 1	Repressed
AV055683	ATP-binding cassette, sub-family A (ABC1), member 3	Repressed
BG074307	ATP-binding cassette, sub-family B (MDR/TAP), member 7	Repressed
AU016579	AU016579	Repressed
AW539206	Autophagy 7-like (S. cerevisiae)	Repressed
AV025785	AV025785	Repressed
AV032859	AV032859	Repressed
AV043303	AV043303	Repressed
AV056563	AV056563	Repressed
AV059610	AV059610	Repressed
AV103135	AV103135	Repressed
AV106281	AV106281	Repressed
AV112179	AV112179	Repressed
AW546231	AW546231	Repressed
AW546688	AW546688	Repressed
AW546823	AW546823	Repressed
AW547555	AW547555	Repressed
AW548385	AW548385	Repressed
AW548764	AW548764	Repressed
AW548883	AW548883	Repressed
AW549514	AW549514	Repressed
AW549924	AW549924	Repressed
AW549974	AW549974	Repressed
AW549995	AW549995	Repressed
AW550140	AW550140	Repressed
AW550503	AW550503	Repressed
AW551176	AW551176	Repressed

AW552922	AW552922	Repressed
AW553165	AW553165	Repressed
AW553509	AW553509	Repressed
AW554034	AW554034	Repressed
AW554061	AW554061	Repressed
AW554113	AW554113	Repressed
AW554960	AW554960	Repressed
AW555055	AW555055	Repressed
AW555492	AW555492	Repressed
AW555660	AW555660	Repressed
AW555786	AW555786	Repressed
AW558484	AW558484	Repressed
BG069998	Beta-site APP cleaving enzyme 1	Repressed
BG072891	BG072891	Repressed
BI076857	BI076857	Repressed
AV061508	Biphenyl hydrolase-like (serine hydrolase, breast epithelial mucin-associated antigen)	Repressed
AV013738	Bone gamma carboxyglutamate protein 1	Repressed
BG073025	Brain glycogen phosphorylase	Repressed
AV025443	Brain protein 17	Repressed
AK011603	Breast carcinoma amplified sequence 3	Repressed
AW538269	C0105E04	Repressed
AW538486	C0108B10	Repressed
AW539380	C0119B07	Repressed
AW544139	C0178C03	Repressed
AW545567	C0195C06	Repressed
BI076582	C0196G08	Repressed
C77236	C77236	Repressed
AV017919	Cadherin 13	Repressed
BG073653	Calcium binding protein 39	Repressed
AV039195	Calcium/calmodulin -dependent protein kinase II gamma	Repressed
AI837783	Calmodulin 3	Repressed
BG067357	Calponin 1	Repressed
AK173059	Calsyntenin 1	Repressed
AA123885	Carnitine palmitoyltransferase 2	Repressed
BG066296	Casein kinase II, beta subunit	Repressed
AV007250	CDC42 small effector 2	Repressed
BG076340	CDNA sequence BC003324	Repressed
AV036285	CDNA sequence BC031181	Repressed
BG065615	CDNA sequence BC053393	Repressed
BG071144	Ceroid-lipofuscinosis, neuronal 8	Repressed
BG072415	CGG triplet repeat binding protein 1	Repressed

AA137292	Chemokine (C-C motif) ligand 19	Repressed
AV037518	Chemokine (C-C motif) ligand 27	Repressed
AV007140	Chondroitin sulfate proteoglycan 4	Repressed
AU021796	Churchill domain containing 1	Repressed
AV031224	Coatomer protein complex, subunit gamma	Repressed
AV083977	Coiled-coil-helix-coiled-coil-helix domain containing 3	Repressed
AV042103	Coiled-coil-helix-coiled-coil-helix domain containing 6	Repressed
BG075721	COMM domain containing 7	Repressed
BG071253	CTD (carboxy-terminal domain, RNA polymerase II, polypeptide A) small phosphatase 1	Repressed
AV043318	Cut-like 1 (Drosophila)	Repressed
BG065546	CXXC finger 5	Repressed
BG069760	Cystathionine beta-synthase	Repressed
BI076595	Cytochrome c oxidase subunit II	Repressed
BG065603	Cytochrome c oxidase subunit IV isoform 1	Repressed
AV088033	Cytochrome c oxidase subunit VIIa polypeptide 2-like	Repressed
AK009614	Cytochrome c oxidase subunit VIIa polypeptide 2-like	Repressed
AV061394	Dehydrogenase/reductase (SDR family) member 4	Repressed
BG073821	Dendritic cell protein GA17	Repressed
AA407888	Desmoplakin	Repressed
AV094650	Developmentally regulated GTP binding protein 1	Repressed
AK117104	Dihydrolipoamide dehydrogenase	Repressed
AV094441	DNA methyltransferase 2	Repressed
BG073397	DNA segment, Chr 10, ERATO Doi 214, expressed	Repressed
AV037205	DNA segment, Chr 10, ERATO Doi 516, expressed	Repressed
AV084131	DNA segment, Chr 10, ERATO Doi 641, expressed	Repressed
AV037272	DNA segment, Chr 11, KL Mohlke 35	Repressed
BG065978	DNA segment, Chr 15, ERATO Doi 50, expressed	Repressed
AI838731	DNA segment, Chr 17, Wayne State University 92, expressed	Repressed
BG074924	DNA segment, Chr 8, Wayne State University 49, expressed	Repressed
AI854079	DNA segment, Chr 9, Wayne State University 20, expressed	Repressed
AW554361	DNA segment, Chr X, Brigham & Women's Genetics 1396 expressed	Repressed
AA153173	Dystroglycan 1	Repressed
BG068850	E1A binding protein p400	Repressed
AK028918	Electron transferring flavoprotein, alpha polypeptide	Repressed
BG072695	Enhancer of rudimentary homolog (Drosophila)	Repressed
AV094590	Eph receptor A5	Repressed
AI838603	Ephrin A5	Repressed

AV091639	Erythrocyte protein band 4.1	Repressed
BG076357	Erythroid differentiation regulator 1	Repressed
AK076696	Eukaryotic translation elongation factor 1 alpha 1	Repressed
AW557711	Eukaryotic translation elongation factor 1 alpha 1	Repressed
AU020246	Eukaryotic translation elongation factor 1 alpha 1	Repressed
AK076696	Eukaryotic translation elongation factor 1 alpha 1	Repressed
AK011951	Eukaryotic translation elongation factor 1 gamma	Repressed
BG063990	Eukaryotic translation elongation factor 1 gamma	Repressed
BG064570	Eukaryotic translation initiation factor 3, subunit 12	Repressed
BG076103	Eukaryotic translation initiation factor 3, subunit 3 (gamma)	Repressed
DV071818	Eukaryotic translation initiation factor 3, subunit 6	Repressed
BC036293	Eukaryotic translation initiation factor 4B	Repressed
AV162437	Expressed sequence AU019823	Repressed
BG069075	expressed sequence AU024582	Repressed
BG073457	Expressed sequence AW549877	Repressed
AV085319	Expressed sequence AW552001	Repressed
AV033293	Expressed sequence AW742319	Repressed
AV083871	F11 receptor	Repressed
AV067628	Fatty acid binding protein 2, intestinal	Repressed
AV017252	F-box and leucine-rich repeat protein 17	Repressed
BG071684	F-box only protein 3	Repressed
AV082197	Ferritin heavy chain 1	Repressed
BG064474	Ferritin light chain 1	Repressed
AV009567	Ferrochelatase	Repressed
AW553234	FGFR1 oncogene partner 2	Repressed
BG065535	Fibronectin type 3 and SPRY domain-containing protein	Repressed
AV004594	Folate hydrolase	Repressed
AV013520	Forkhead box O3a	Repressed
AV000213	Fumarylacetoacetate hydrolase domain containing 2A	Repressed
AV104104	G protein pathway suppressor 1	Repressed
AV016436	G protein-coupled receptor 133	Repressed
AK053238	Gap junction membrane channel protein alpha 1	Repressed
BG064678	GLE1 RNA export mediator-like (yeast	Repressed
AV090250	Glucocorticoid induced transcript 1	Repressed
AV083198	Glucosidase, beta, acid	Repressed
AV084290	Glutamate dehydrogenase 1	Repressed
W15737	Glutathione S-transferase, mu 1	Repressed
BG071068	Guanine nucleotide binding protein, beta 1	Repressed
NM_008197	H1 histone family, member 0	Repressed
AK145379	H19 fetal liver mRNA	Repressed
AK088075	H3 histone, family 3B	Repressed
BG063840	H3011D05	Repressed

BG064868	H3024G04	Repressed
BG067648	H3056F09	Repressed
BG068899	H3070E06	Repressed
BG073317	H3121C03	Repressed
BG073659	H3126E11	Repressed
BG073995	H3129F06	Repressed
BG074801	H3139D11	Repressed
BG076402	H3159D09	Repressed
AK088844	Heat shock protein 1 (chaperonin)	Repressed
AV104309	Heat shock protein 8	Repressed
BG065740	Heterogeneous nuclear ribonucleoprotein A1	Repressed
AK135297	High mobility group box 2	Repressed
AV037365	Histocompatibility 2, D region locus 1	Repressed
BG071219	Histocompatibility 2, D region locus 1	Repressed
AV081997	Histocompatibility 2, D region locus 1	Repressed
AU041598	Histocompatibility 2, K1, K region	Repressed
AV041015	Histone deacetylase 11	Repressed
AV009996	HLA-B-associated transcript 3	Repressed
AI840878	Homeobox only domain	Repressed
AV094789	Homer homolog 2 (Drosophila)	Repressed
AV094520	HpaII tiny fragments locus 9c	Repressed
AW559034	Huntingtin interacting protein 2	Repressed
AV010091	Iduronidase, alpha-L-	Repressed
AV086425	IlvB (bacterial acetolactate synthase)-like	Repressed
AV015004	Immediate early response 3 interacting protein 1	Repressed
AV018921	Inhibitor of growth family, member 5	Repressed
AV083325	Inositol hexaphosphate kinase 1	Repressed
AI838414	Karyopherin (importin) alpha 1	Repressed
BG073156	Kelch-like 17 (Drosophila)	Repressed
AK051462	Kelch-like 7 (Drosophila)	Repressed
BY356335	Laminin receptor 1 (ribosomal protein SA)	Repressed
BG071088	Lemur tyrosine kinase 2	Repressed
AV103984	Leptin receptor overlapping transcript	Repressed
BG075528	Leucine-rich repeats and immunoglobulin-like domains 3	Repressed
BG065405	Leukocyte receptor cluster (LRC) member 4	Repressed
BG065703	Lin 10 homolog (C. elegans)	Repressed
AV095097	LOC433110	Repressed
BG075361	Low density lipoprotein receptor-related protein 1	Repressed
BG064652	Macrophage erythroblast attacher	Repressed
BG072486	MAF1 homolog (yeast)	Repressed
AV031462	Malignant T cell amplified sequence 1	Repressed

BG073016	Membrane associated guanylate kinase, WW and PDZ domain containing 2	Repressed
AA034768	Mid1 interacting protein 1 (gastrulation specific G12-like (zebrafish))	Repressed
AV073432	Mitochondrial ribosomal protein L30	Repressed
AV032283	Mitogen activated protein kinase 10	Repressed
BG066357	Mitogen activated protein kinase kinase kinase 12	Repressed
AV089761	Morf4 family associated protein 1	Repressed
BC010209	Morf4 family associated protein 1	Repressed
AV112997	Mortality factor 4 like 1	Repressed
AK140931	Mortality factor 4 like 2	Repressed
AV055041	Myeloid/lymphoid or mixed-lineage leukemia 5	Repressed
BG074849	Myotrophin	Repressed
BC022734	N-acetylneuraminate pyruvate lyase	Repressed
AV033765	NADH dehydrogenase (ubiquinone) 1 alpha subcomplex 10	Repressed
BG068193	Nedd4 family interacting protein 1	Repressed
BG071218	N-myc downstream regulated gene 2	Repressed
AK172452	Nonagouti	Repressed
W53283	Notch gene homolog 3 (Drosophila)	Repressed
AV058809	N-terminal Asn amidase	Repressed
BE685736	Nuclear factor of activated T-cells, cytoplasmic, calcineurin-dependent 3	Repressed
BG065226	Nuclear receptor coactivator 4	Repressed
AV036516	Nucleophosmin 1	Repressed
BG071420	Nucleoredoxin	Repressed
AV065416	Nucleosome assembly protein 1-like 1	Repressed
AV026104	NudC domain containing 3	Repressed
BG069614	Nudix (nucleoside diphosphate linked moiety X)-type motif 7	Repressed
BC006937	OCIA domain containing 1	Repressed
AV073472	Olfactomedin 1	Repressed
BG073162	Oxysterol binding protein-like 1A	Repressed
BB667069	PDZ and LIM domain 2	Repressed
AV072432	Peptidylprolyl isomerase A	Repressed
BG067497	Peroxiredoxin 1	Repressed
AV103718	Phenylalanine-tRNA synthetase 2 (mitochondrial)	Repressed
BG074363	Phosphatidylinositol glycan, class Q	Repressed
BG064745	Phosphoglycerate kinase 1	Repressed
AW556756	Phosphotriesterase related	Repressed
AW556262	Piwi like homolog 2 (Drosophila)	Repressed
AV022767	Pleiotrophin	Repressed

AV065300	Polyadenylate-binding protein-interacting protein 2	Repressed
AV162289	Polymerase (DNA-directed), delta interacting protein 2	Repressed
AV094752	Polymerase (RNA) II (DNA directed) polypeptide E	Repressed
BG066638	POU domain, class 6, transcription factor 1	Repressed
AV032030	Praja1, RING-H2 motif containing	Repressed
AV015754	Pre B-cell leukemia transcription factor 1	Repressed
BG066376	Progressive ankylosis	Repressed
AK034723	Proliferating cell nuclear antigen	Repressed
BG067626	Prostaglandin F2 receptor negative regulator	Repressed
AV074598	Protein kinase C and casein kinase substrate in neurons 3	Repressed
W17530	Protein kinase, cAMP dependent, catalytic, alpha	Repressed
AK157914	Protein phosphatase 1, catalytic subunit, beta isoform	Repressed
AV037994	Protein phosphatase 2 (formerly 2A), regulatory subunit B, alpha	Repressed
AV085826	Purine rich element binding protein B	Repressed
AK034242	RAB18, member RAS oncogene family	Repressed
AV036483	RAB9, member RAS oncogene family	Repressed
AV085507	Receptor (calcitonin) activity modifying protein 2	Repressed
AV134917	REST co-repressor 2	Repressed
AU040633	Rho guanine nucleotide exchange factor (GEF) 11	Repressed
BG066529	Ribonuclease T2	Repressed
AV059437	Ribonuclease, RNase A family, 1 (pancreatic)	Repressed
AK168807	Ribosomal protein L15	Repressed
AV095469	Ribosomal protein L19	Repressed
BG072625	Ribosomal protein L19	Repressed
AI322261	Ribosomal protein L19	Repressed
AV093793	Ribosomal protein L24	Repressed
BG073775	Ribosomal protein L26	Repressed
BC098497	Ribosomal protein L3	Repressed
BG076117	Ribosomal protein L3	Repressed
BG065397	Ribosomal protein L30	Repressed
BC037217	Ribosomal protein L30	Repressed
AV106965	Ribosomal protein L32	Repressed
BG072856	Ribosomal protein L35	Repressed
BG063719	Ribosomal protein L4	Repressed
BU511604	Ribosomal protein L5	Repressed
BG075206	Ribosomal protein L6	Repressed
BG075484	Ribosomal protein L7	Repressed
BF121988	Ribosomal protein L7	Repressed
AV095054	Ribosomal protein L9	Repressed
AV103708	Ribosomal protein L9	Repressed
AV093721	Ribosomal protein S11	Repressed

BG073105	Ribosomal protein S17	Repressed
BG073219	Ribosomal protein S26	Repressed
AK143603	Ribosomal protein S3	Repressed
BG073705	Ribosomal protein S3	Repressed
BG069771	Ribosomal protein S3a	Repressed
BU515379	Ribosomal protein S4, X-linked	Repressed
BF581166	Ribosomal protein S6	Repressed
AV094961	Ribosomal protein S6	Repressed
CA461814	Ribosomal protein S8	Repressed
BG074831	RIKEN cDNA 0610031J06 gene	Repressed
BG066600	RIKEN cDNA 0610039N19 gene	Repressed
BI688229	RIKEN cDNA 0610040D20 gene	Repressed
AV078318	RIKEN cDNA 1100001I22 gene	Repressed
BG068553	RIKEN cDNA 1110003E01 gene	Repressed
AV094539	RIKEN cDNA 1110004B13 gene	Repressed
AV068654	RIKEN cDNA 1110005A23 gene	Repressed
BC034085	RIKEN cDNA 1110007A06 gene	Repressed
BG067962	RIKEN cDNA 1110020C13 gene	Repressed
BG069789	RIKEN cDNA 1110021H02 gene	Repressed
AV103649	RIKEN cDNA 1110030J09 gene	Repressed
BG075217	RIKEN cDNA 1200006O19 gene	Repressed
AV078181	RIKEN cDNA 1200009B18 gene	Repressed
BG072201	RIKEN cDNA 1200015A19 gene	Repressed
BG064450	RIKEN cDNA 1200015F23 gene	Repressed
AV078454	RIKEN cDNA 1300007B12 gene	Repressed
AV033648	RIKEN cDNA 1500010J02 gene	Repressed
AV035241	RIKEN cDNA 1600014C10 gene	Repressed
BG076352	RIKEN cDNA 1700020I14 gene	Repressed
BG076310	RIKEN cDNA 1810014L12 gene	Repressed
AY151252	RIKEN cDNA 1810044A24 gene	Repressed
AV038329	RIKEN cDNA 1810045K07 gene	Repressed
BG072328	RIKEN cDNA 1810046J19 gene	Repressed
AV050158	RIKEN cDNA 1810047C23 gene	Repressed
AV018850	RIKEN cDNA 1810058I24 gene	Repressed
AV061197	RIKEN cDNA 1810073G14 gene	Repressed
AV066125	RIKEN cDNA 2010106G01 gene	Repressed
AV094522	RIKEN cDNA 2210016L21 gene	Repressed
AV085439	RIKEN cDNA 2310002J15 gene	Repressed
AV084769	RIKEN cDNA 2310003P10 gene	Repressed
AV083988	RIKEN cDNA 2310004K06 gene	Repressed
AV140878	RIKEN cDNA 2310008M10 gene	Repressed
AV088072	RIKEN cDNA 2310034L04 gene	Repressed

AI840046	RIKEN cDNA 2310047D13 gene	Repressed
BG067161	RIKEN cDNA 2310067E08 gene	Repressed
AV084923	RIKEN cDNA 2310076L09 gene	Repressed
AV093026	RIKEN cDNA 2310076O14 gene	Repressed
AV085399	RIKEN cDNA 2410005O16 gene	Repressed
BG076336	RIKEN cDNA 2410042D21 gene	Repressed
BG071504	RIKEN cDNA 2500002L14 gene	Repressed
AV104173	RIKEN cDNA 2510003E04 gene	Repressed
AV170924	RIKEN cDNA 2510048O06 gene	Repressed
AV041527	RIKEN cDNA 2600001J17 gene	Repressed
AK138718	RIKEN cDNA 2600011C06 gene	Repressed
BG070614	RIKEN cDNA 2610009E16 gene	Repressed
AV112669	RIKEN cDNA 2610014I16 gene	Repressed
BG068342	RIKEN cDNA 2610044O15 gene	Repressed
BG072271	RIKEN cDNA 2610510H01 gene	Repressed
BG065216	RIKEN cDNA 2700060E02 gene	Repressed
AV134975	RIKEN cDNA 2810012H18 gene	Repressed
BG076192	RIKEN cDNA 2810021B07 gene	Repressed
BG064609	RIKEN cDNA 2810409H07 gene	Repressed
BG065981	RIKEN cDNA 2900064A13 gene	Repressed
BG073867	RIKEN cDNA 2900097C17 gene	Repressed
AV162274	RIKEN cDNA 3110001I20 gene	Repressed
BG071566	RIKEN cDNA 3110006P09 gene	Repressed
AV050135	RIKEN cDNA 4833420G17 gene	Repressed
BG068883	RIKEN cDNA 4833439L19 gene	Repressed
AW909347	RIKEN cDNA 4921504P13 gene	Repressed
AK037008	RIKEN cDNA 4921506J03 gene	Repressed
AA410046	RIKEN cDNA 4921517N04 gene	Repressed
AV016060	RIKEN cDNA 4930469P12 gene	Repressed
BG064832	RIKEN cDNA 4930542G03 gene	Repressed
BG065604	RIKEN cDNA 4933439C20 gene	Repressed
BG074875	RIKEN cDNA 5031439G07 gene	Repressed
AV060544	RIKEN cDNA 5730589K01 gene	Repressed
BG075899	RIKEN cDNA 5830434P21 gene	Repressed
BG075904	RIKEN cDNA 6330578E17 gene	Repressed
BG075587	RIKEN cDNA 7530414M10 gene	Repressed
AV086198	RIKEN cDNA 9130404D14 gene	Repressed
AA221782	RIKEN cDNA 9330164J24 gene	Repressed
AV015921	RIKEN cDNA 9630058J23 gene	Repressed
BG067375	RIKEN cDNA A130038L21 gene	Repressed
BG073049	RIKEN cDNA C030011O14 gene	Repressed
BG073325	RIKEN cDNA F830004M19 gene	Repressed

BG073275	Ring finger protein 10	Repressed
XM_135065	Ring finger protein 7	Repressed
BG067793	RNA binding motif protein 18	Repressed
AV061211	RNA binding motif protein 3	Repressed
AV086400	RNA binding motif protein 3	Repressed
AV106468	RNA terminal phosphate cyclase-like 1	Repressed
BG063216	RNA, U64 small nucleolar	Repressed
AV056620	Ropporin 1-like	Repressed
AV054009	Scleraxis	Repressed
BG074808	SEC31-like 1 (S. cerevisiae)	Repressed
DV038944	Selenoprotein K	Repressed
BG074727	Sema domain, immunoglobulin domain (Ig), transmembrane domain (TM) and short cytoplasmic domain, (semaphorin) 4G	Repressed
AV088659	Serine (or cysteine) proteinase inhibitor, clade B (ovalbumin), member 12	Repressed
AV141694	Signal recognition particle 54	Repressed
AI837820	Signal recognition particle 68	Repressed
BG071789	Signaling lymphocytic activation molecule family member 1	Repressed
BG076120	SMAD specific E3 ubiquitin protein ligase 1	Repressed
AK132746	Small EDRK-rich factor 2	Repressed
AV013432	Small proline rich-like 5	Repressed
AI838562	SMC6 structural maintenance of chromosomes 6-like 1 (yeast)	Repressed
AV061426	SMT3 suppressor of mif two 3 homolog 1 (yeast)	Repressed
BG075546	Solute carrier family 14 (urea transporter), member 1	Repressed
AV030530	Solute carrier family 25 (mitochondrial carrier, palmitoylcarnitine transporter), member 29	Repressed
AV011553	Solute carrier family 30 (zinc transporter), member 4	Repressed
BG072644	Solute carrier family 38, member 2	Repressed
AV040590	Sorbitol dehydrogenase 1	Repressed
BG071783	Spastic paraplegia 21 homolog (human)	Repressed
AV093840	Spermatogenesis associated, serine-rich 1	Repressed
BG072707	Spermidine/spermine N1-acetyl transferase 1	Repressed
AV010913	SPFH domain family, member 2	Repressed
BG070131	Spindlin	Repressed
AV104548	Splicing factor 3b, subunit 1	Repressed
BG075142	Splicing factor, arginine/serine-rich 2 (SC-35)	Repressed
BG064951	Splicing factor, arginine/serine-rich 3 (SRp20)	Repressed
AV053031	Stearoyl-Coenzyme A desaturase 1	Repressed
BG074964	Steroid receptor RNA activator 1	Repressed
AK087232	Sterol carrier protein 2, liver	Repressed

BC068152	Stromal cell derived factor 4	Repressed
AV021056	Supervillin	Repressed
BG067098	Suppressor of cytokine signaling 7	Repressed
BG073955	Swi/SNF related matrix associated, actin dependent regulator of chromatin, subfamily a-like 1	Repressed
AK147685	SWI/SNF related, matrix associated, actin dependent regulator of chromatin, subfamily b, member 1	Repressed
AI841363	Synaptotagmin 11	Repressed
AK081173	Syntaxin 5A	Repressed
BG073933	TAF13 RNA polymerase II, TATA box binding protein (TBP)-associated factor	Repressed
AU017673	TatD DNase domain containing 2	Repressed
BG067445	Taxilin	Repressed
AV141372	T-cell leukemia, homeobox 2	Repressed
AV015100	T-cell receptor beta, variable 13	Repressed
AW552118	Terf1 (TRF1)-interacting nuclear factor 2	Repressed
AV090034	Testis expressed gene 2	Repressed
BG075966	Tetraspanin 9	Repressed
BG068537	Tetratricopeptide repeat domain 12	Repressed
AV088458	Thimet oligopeptidase 1	Repressed
BG065641	Thioredoxin domain containing 5	Repressed
AK165598	Translin	Repressed
BG066092	Translocase of outer mitochondrial membrane 20 homolog (yeast)	Repressed
AV071376	Transmembrane protein 14C	Repressed
AI840745	Trans-prenyltransferase	Repressed
AW550308	Tripartite motif-containing 44	Repressed
C87464	Tumor protein D52	Repressed
BG072696	Tumor protein, translationally-controlled 1	Repressed
AV095043	Tumor protein, translationally-controlled 1	Repressed
AV053280	Twisted gastrulation homolog 1 (Drosophila)	Repressed
AU023376	Tyrosinase	Repressed
AV094502	Tyrosine 3-monooxygenase/tryptophan 5-monooxygenase activation protein, theta polypeptide	Repressed
AK162683	Ubiquitin specific protease 16	Repressed
BG066549	Ubiquitin-conjugating enzyme E2L 3	Repressed
BG071748	Ubiquitin-conjugating enzyme E2S	Repressed
AV003213	UDP glucuronosyltransferase 1 family, polypeptide A6A	Repressed
AI840523	UI-M-AM0-adp-b-05-0-UI	Repressed
AI840610	UI-M-AM0-adq-d-06-0-UI	Repressed
BG066564	Vacuolar protein sorting 24 (yeast)	Repressed
AK028264	Valosin containing protein	Repressed

AV086153	Von Hippel-Lindau binding protein 1	Repressed
AV073394	V-set and immunoglobulin domain containing 2	Repressed
AV088602	WD repeat domain 23	Repressed
AV135866	WD repeat domain 33	Repressed
BG076278	Yippee-like 3 (Drosophila)	Repressed
BG075353	Zinc finger, FYVE domain containing 21	Repressed
BG067835	Zinc finger, matrin type 2	Repressed
BG074596	Zona pellucida glycoprotein 2	Repressed
AV050667	1810014K16	Induced
AV061715	1810074P22	Induced
AV065496	2010016E13	Induced
AV067190	2010203M03	Induced
AV085715	2310015A10	Induced
AV090113	2310047E02	Induced
AV113932	2610025P03	Induced
AV114430	2610034B18	Induced
AV114610	2610035D17	Induced
AV135009	2810012L14	Induced
BG070894	5',3'-nucleotidase, cytosolic	Induced
BG073466	A630082K20Rik	Induced
AA163512	AA163512	Induced
BG063206	AarF domain containing kinase 2	Induced
BG074852	AarF domain containing kinase 5	Induced
BG067124	Abhydrolase domain containing 4	Induced
AV094930	Achalasia, adrenocortical insufficiency, alacrimia	Induced
AV043702	Actin-like 6A	Induced
BG064927	AE binding protein 2	Induced
BG071714	Apoptotic chromatin condensation inducer 1	Induced
AK009243	Arginine vasopressin-induced 1	Induced
BG071486	Arginyl-tRNA synthetase-like	Induced
BG072792	Arrestin domain containing 1	Induced
BG075470	Ash2 (absent, small, or homeotic)-like (Drosophila)	Induced
AV051025	ATPase type 13A2	Induced
AV055189	AV055189	Induced
BG063473	BG063473	Induced
BG064541	BG064541	Induced
BG071259	BG071259	Induced
AV103873	BolA-like 1 (E. coli)	Induced
AK140279	bolA-like 2 (E. coli)	Induced
AA408701	C0031G05	Induced
AW538759	C0111E01	Induced
AW543765	C0173H02	Induced

BI076559	C0181G01	Induced
AW544771	C0185G01	Induced
AV035186	Calcium regulated heat stable protein 1	Induced
BG071245	Carbonyl reductase 2	Induced
AK054334	Catechol-O-methyltransferase domain containing 1	Induced
AV094547	CD68 antigen	Induced
AU051332	CDC28 protein kinase 1b	Induced
BG064971	CDNA sequence BC011248	Induced
BG073680	Cell division cycle 34 homolog (S. cerevisiae)	Induced
AV095264	Cell division cycle associated 3	Induced
BG068799	Cell division cycle associated 5	Induced
BG068213	Chromodomain helicase DNA binding protein 1-like	Induced
BG067086	Claspin homolog (Xenopus laevis)	Induced
BG063980	Coiled-coil-helix-coiled-coil-helix domain containing 2	Induced
AV095113	COX10 homolog, cytochrome c oxidase assembly protein, heme A: farnesyltransferase (yeast)	Induced
BG063423	CTF18, chromosome transmission fidelity factor 18 homolog (S. cerevisiae)	Induced
BG067302	Cytochrome c oxidase subunit VIb polypeptide 2	Induced
BG068047	Cytokine induced apoptosis inhibitor 1	Induced
BG068906	Cytoplasmic polyadenylation element binding protein 1	Induced
BG070608	DEAD (Asp-Glu-Ala-Asp) box polypeptide 18	Induced
AV055700	DEAD (Asp-Glu-Ala-Asp) box polypeptide 55	Induced
AV094905	Differentially expressed in FDCP 6	Induced
AI854081	DNA segment, Chr 10, Wayne State University 102, expressed	Induced
AA408470	DNA segment, Chr 15, Wayne State University 169, expressed	Induced
AV042920	DNA segment, Chr 2, ERATO Doi 750, expressed	Induced
BG064577	DNA segment, Chr 2, Wayne State University 81, expressed	Induced
AV084455	Dolichyl-phosphate mannosyltransferase polypeptide 3	Induced
BG074275	DOT1-like, histone H3 methyltransferase (S. cerevisiae)	Induced
BG069569	Era (G-protein)-like 1 (E. coli)	Induced
AV030670	Exosome component 1	Induced
AV043571	Exosome component 5	Induced
BG074217	Expressed sequence AA409316	Induced
BG071781	Expressed sequence AU044856	Induced
BG064150	Expressed sequence AW544865	Induced
BG074780	Fanconi anemia, complementation group L	Induced
BG074856	Fas-activated serine/threonine kinase	Induced
BG063110	Fibronectin type III domain containing 3a	Induced

BG064068	G patch domain containing 4	Induced
BG063112	Gem (nuclear organelle) associated protein 5	Induced
AV085862	Gene trap locus F3a	Induced
AV103331	Gene trap ROSA b-geo 22	Induced
BG071448	General transcription factor IIIC, polypeptide 5	Induced
AA080218	GLI-Kruppel family member HKR3	Induced
AV002974	Glutathione peroxidase 6	Induced
BG063959	H3013G12	Induced
BG065649	H3033F08	Induced
BG066288	H3041B10	Induced
BG067980	H3060D07	Induced
BG068714	H3068E09	Induced
BG071755	H3102H02	Induced
BG073624	H3126B09	Induced
BG075638	H3149F07	Induced
AV085682	HIV-1 tat interactive protein, homolog (human)	Induced
AA245472	Homeo box C4	Induced
AV090011	Hypoxia-inducible factor 1, alpha subunit inhibitor	Induced
AA137960	IL2-inducible T-cell kinase	Induced
AU043663	Importin 4	Induced
AV067418	Interleukin 18 binding protein	Induced
AV054283	JTV1 gene	Induced
K02108	Keratin (epidermal) intermediate filament subunit II, mRNA	Induced
BG074443	Lectin, galactose binding, soluble 7	Induced
BG074816	Leucine zipper protein 5	Induced
BG071768	LOC545341	Induced
BG064523	Longevity assurance homolog 5 (S. cerevisiae)	Induced
AV037696	LSM4 homolog, U6 small nuclear RNA associated (S. cerevisiae)	Induced
BF118616	Lymphocyte antigen 6 complex, locus A	Induced
BG068773	MAM domain containing 1	Induced
AV065609	Mitochondrial ribosomal protein L43	Induced
AV026345	Mitogen activated protein kinase kinase 7	Induced
AU045159	Mki67 (FHA domain) interacting nucleolar phosphoprotein	Induced
BG064611	MUS81 endonuclease homolog (yeast)	Induced
D50311	Myocyte enhancer factor 2B	Induced
AV103724	N-acetyl galactosaminidase, alpha	Induced
BG070586	NHP2 non-histone chromosome protein 2-like 1 (S. cerevisiae)	Induced
AI322376	Notchless homolog 1 (Drosophila)	Induced
BG074098	Nucleoplasmin 3	Induced

AV037674	Nudix (nucleoside diphosphate linked moiety X)-type motif 14	Induced
BG068580	PET112-like (yeast)	Induced
BG068556	Phosphatidylinositol glycan, class F	Induced
AV114515	Phytoceramidase, alkaline	Induced
AV007317	Polymerase (DNA directed), lambda	Induced
AV036690	Prolactin-like protein F	Induced
AI841376	Protein phosphatase 5, catalytic subunit	Induced
AV008153	Protein tyrosine phosphatase, receptor type, f polypeptide (PTPRF), interacting protein (liprin), alpha 4	Induced
AV054713	Ral GEF with PH domain and SH3 binding motif 2	Induced
BG063278	RAP2B, member of RAS oncogene family	Induced
AV073480	Retinol dehydrogenase 12	Induced
AV170878	Rhotekin	Induced
AV049214	RIKEN cDNA 0610006I08 gene	Induced
BG063690	RIKEN cDNA 0610009O03 gene	Induced
BG070781	RIKEN cDNA 1110001A12 gene	Induced
AV006844	RIKEN cDNA 1110001M19 gene	Induced
AV011145	RIKEN cDNA 1110002E23 gene	Induced
BG074224	RIKEN cDNA 1110002M09 gene	Induced
BC018244	RIKEN cDNA 1110007C05 gene	Induced
BG064822	RIKEN cDNA 1110008B24 gene	Induced
AV010817	RIKEN cDNA 1110029E03 gene	Induced
BG064119	RIKEN cDNA 1110056N09 gene	Induced
BG063492	RIKEN cDNA 1110064N10 gene	Induced
BG074000	RIKEN cDNA 1200013B22 gene	Induced
AV055765	RIKEN cDNA 1300018I05 gene	Induced
BG066812	RIKEN cDNA 1600027N09 gene	Induced
AV056014	RIKEN cDNA 1700121I08 gene	Induced
BG070542	RIKEN cDNA 1810006K21 gene	Induced
AV048928	RIKEN cDNA 1810008A18 gene	Induced
AV057471	RIKEN cDNA 1810009K13 gene	Induced
AV020251	RIKEN cDNA 1810061M12 gene	Induced
AV060457	RIKEN cDNA 1810063B05 gene	Induced
BG075840	RIKEN cDNA 2010100O12 gene	Induced
AV088623	RIKEN cDNA 2010100O12 gene	Induced
BG075773	RIKEN cDNA 2010109N14 gene	Induced
BG064696	RIKEN cDNA 2310009B15 gene	Induced
BG063171	RIKEN cDNA 2310014H01 gene	Induced
BG072006	RIKEN cDNA 2310039H08 gene	Induced
BG068304	RIKEN cDNA 2310061C15 gene	Induced
BG072013	RIKEN cDNA 2410003K15 gene	Induced

AV085671	RIKEN cDNA 2410012H22 gene	Induced
AV094758	RIKEN cDNA 2410015M20 gene	Induced
BG063089	RIKEN cDNA 2510002D24 gene	Induced
AV023921	RIKEN cDNA 2510039O18 gene	Induced
BG067547	RIKEN cDNA 2600010E01 gene	Induced
AV103928	RIKEN cDNA 2610019I03 gene	Induced
BG071030	RIKEN cDNA 2610304G08 gene	Induced
BY711361	RIKEN cDNA 2610528J11 gene	Induced
AV140980	RIKEN cDNA 2700062C07 gene	Induced
AV141800	RIKEN cDNA 2700078E11 gene	Induced
BG073790	RIKEN cDNA 2700094K13 gene	Induced
BG073345	RIKEN cDNA 2810028N01 gene	Induced
BG064731	RIKEN cDNA 2810475A17 gene	Induced
AV043034	RIKEN cDNA 2900016G09 gene	Induced
BG071176	RIKEN cDNA 2900017G11 gene	Induced
AV162171	RIKEN cDNA 3110001D03 gene	Induced
BG068253	RIKEN cDNA 3110031B13 gene	Induced
AV171702	RIKEN cDNA 3300001M20 gene	Induced
BG069307	RIKEN cDNA 4631424J17 gene	Induced
AV081325	RIKEN cDNA 4632409L19 gene	Induced
AA408693	RIKEN cDNA 4633402N23 gene	Induced
BG071969	RIKEN cDNA 4732473B16 gene	Induced
AV085647	RIKEN cDNA 9130227C08 gene	Induced
BG073755	RIKEN cDNA 9330182L06 gene	Induced
AV061212	RIKEN cDNA 9430008C03 gene	Induced
BG075690	RIKEN cDNA B430218L07 gene	Induced
BG064513	RIKEN cDNA C130032J12 gene	Induced
BG067439	RIKEN cDNA E430024C06 gene	Induced
BG070490	RIKEN cDNA E430025E21 gene	Induced
BG063889	Ring finger protein 41	Induced
AV094723	RNA binding motif protein 17	Induced
AV054939	RNA polymerase 1-3	Induced
AV051644	RNA, U17d small nucleolar	Induced
BG064942	SCY1-like 2 (S. cerevisiae)	Induced
BG063172	Sec61, alpha subunit 2 (S. cerevisiae)	Induced
BG065606	Serine (or cysteine) proteinase inhibitor, clade B, member 6c	Induced
AV019805	SH2-B PH domain containing signaling mediator 1	Induced
BG074318	Small nuclear ribonucleoprotein D2	Induced
AV088345	Small proline rich-like 1	Induced
NM_009265	Small proline-rich protein 1B	Induced
BG064948	Spindle pole body component 24 homolog (S. cerevisiae)	Induced

AV002587	Spindle pole body component 25 homolog (S. cerevisiae)	Induced
BG069886	Splicing factor 3b, subunit 4	Induced
BG074410	Splicing factor 3b, subunit 5	Induced
BG062975	SRY-box containing gene 13	Induced
BG072617	Stem-loop binding protein	Induced
AV095249	Surfeit gene 6	Induced
BG070492	Synaptoporin	Induced
BG067535	Tetratricopeptide repeat domain 4	Induced
AV009795	Thioesterase, adipose associated	Induced
BC034843	Thrombospondin, type I domain containing 6	Induced
AA125268	Tnf receptor-associated factor 7	Induced
AW553200	Transcriptional regulator, SIN3A (yeast)	Induced
AV162425	Translocase of inner mitochondrial membrane 23 homolog (yeast)	Induced
AV083948	Translocase of inner mitochondrial membrane 9 homolog (yeast)	Induced
AV006659	Translocator of inner mitochondrial membrane 17b	Induced
AV025038	Transmembrane protein 43	Induced
AU021079	Transmembrane protein induced by tumor necrosis factor alpha	Induced
AA162497	Tripartite motif protein 27	Induced
BG071659	Tuftelin 1	Induced
AV083936	Tumor differentially expressed 2-like	Induced
AW558029	Ubiquinol-cytochrome c reductase (6.4kD) subunit	Induced
AV018519	Ubiquitin-associated protein 2	Induced
AV015860	Ubiquitin-like, containing PHD and RING finger domains, 1	Induced
BG064298	UDP-Gal:betaGlcNAc beta 1,4-galactosyltransferase, polypeptide 3	Induced
AI893759	Vang, van gogh-like 1 (Drosophila)	Induced
BG066921	WD repeat domain 50	Induced
AV088360	Williams Beuren syndrome chromosome region 21	Induced
BG063774	Zinc finger, CCHC domain containing 9	Induced
BG072903	Zinc finger, RAN-binding domain containing 1	Induced
